# UPLC-MS^E^ Profiling of Phytoplankton Metabolites: Application to the Identification of Pigments and Structural Analysis of Metabolites in *Porphyridium purpureum*

**DOI:** 10.3390/md13042541

**Published:** 2015-04-22

**Authors:** Camille Juin, Antoine Bonnet, Elodie Nicolau, Jean-Baptiste Bérard, Romain Devillers, Valérie Thiéry, Jean-Paul Cadoret, Laurent Picot

**Affiliations:** 1 University of La Rochelle, UMRi CNRS 7266 LIENSs, 17042 La Rochelle, France; E-Mails: camille.juin@univ-lr.fr (C.J.); romain.devillers@etudiant.univ-lr.fr (R.D.); valerie.thiery@univ-lr.fr (V.T.); 2 Platform for the High Resolution Analysis of Biomolecules, University of La Rochelle, UMRi CNRS 7266 LIENSs, 17042 La Rochelle, France; E-Mail: antoine.bonnet@univ-lr.fr; 3 IFREMER, Laboratory BRM/PBA, Rue de l’Ile d’Yeu, 44311 Nantes, France; E-Mails: elodie.nicolau@ifremer.fr (E.N.); jean.baptiste.berard@ifremer.fr (J.-B.B.); jean.paul.cadoret@ifremer.fr (J.-P.C.)

**Keywords:** carotenoid, chlorophyll, dereplication, divinyl chlorophyll *a*, galactosyldiacylglycerol, gracilamide, mass spectrometry, MS^E^, phytoplankton, pigment, *Porphyridium purpureum*, UPLC

## Abstract

A fast and high-resolution UPLC-MS^E^ analysis was used to identify phytoplankton pigments in an ethanol extract of *Porphyridium purpureum* (*Pp*) devoid of phycobiliproteins. In a first step, 22 standard pigments were analyzed by UPLC-MS^E^ to build a database including retention time and accurate masses of parent and fragment ions. Using this database, seven pigments or derivatives previously reported in *Pp* were unequivocally identified: β,β-carotene, chlorophyll *a*, zeaxanthin, chlorophyllide *a*, pheophorbide *a*, pheophytin *a*, and cryptoxanthin. Minor amounts of Divinyl chlorophyll *a*, a chemotaxonomic pigment marker for prochlorophytes, were also unequivocally identified using the database. Additional analysis of ionization and fragmentation patterns indicated the presence of ions that could correspond to hydroxylated derivatives of chlorophyll *a* and pheophytin *a*, produced during the ethanolic extraction, as well as previously described galactosyldiacylglycerols, the thylakoid coenzyme plastoquinone, and gracilamide B, a molecule previously reported in the red seaweed *Gracillaria asiatica*. These data point to UPLC-MS^E^ as an efficient technique to identify phytoplankton pigments for which standards are available, and demonstrate its major interest as a complementary method for the structural elucidation of ionizable marine molecules.

## 1. Introduction

Phytoplankton species present a high genetic and metabolic diversity, and evolved a wide range of photoprotective and photosynthetic pigments capable of collectively harvesting most of the wavelengths of visible light available in underwater marine habitats [1,2,3,4]. In spite of their lability, complex taxonomic distribution, and variable expression, phytoplankton pigments have a great interest as chemotaxonomic markers to identify species or taxa, and assess their abundance, productivity, and biodiversity in seawater samples [1,5,6]. The identification and dosage of phytoplankton pigments and derivatives in sediments also have potential for assessing ocean productivity, modeling the spatial and seasonal sedimentation and hydrodynamic processes, and demonstrating local or global marine ecosystem changes [7,8]. In addition, many phytoplankton pigments exhibit physico-chemical, biological, and pharmacological activities that allow us to consider their possible use for biotechnological or health applications [9,10,11,12,13,14,15,16].

In the last decades, HPLC has emerged as the gold standard analytical tool for qualitative and quantitative analysis of phytoplankton pigments in seawater and culture samples because of its easiness, rapidity, sensitivity, and resolution [2,11,17,18,19,20,21]. Optimization of HPLC performance demonstrated that in addition to the major pigments easily identified by their absorption spectrum, band ratio, and polarity, several minor unidentified chlorophyll and carotenoid derivatives are usually present in extracts from environmental samples or cultivated species (e.g., unknown carotenoids detected in [22]). The UV absorption characteristics of these pigments allow us to classify them as chlorophyll or carotenoid derivatives, but the determination of their structure requires high-resolution MS analysis and additional purification for NMR analysis. As a consequence, because of their very low abundance, these minor pigments usually remain unidentified, in spite of their possible interest as chemotaxonomic markers or for biotechnological or biomedical applications. They can correspond to molecules effectively present in living algal cells, to biosynthetic precursors and intermediates, or to artifact or natural derivatives produced by the alteration of chlorophylls or carotenoids in environmental conditions or during extraction and/or purification [12,19,23]. Analysis of such minor pigments has benefited from the recent developments of UPLC, which offer significant advantages compared to HPLC, in term of resolution, sensitivity, and rapidity. As HPLC, UPLC can be coupled to UV-vis detectors and MS analyzers [24,25,26,27,28,29] and the development of new MS methods and devices, such as MS^E^, also offers new possibilities for the analysis of complex samples. In MS^E^, the metabolites reaching the mass source after HPLC or UPLC are subjected to ionization in low- and high-energy collision modes alternating at medium frequency (about 30 Hz). All molecules ionizing at low collision energy are detected and identified as parent ions. A few milliseconds afterward, switching to the high-energy collision mode induces the fragmentation of parent ions, and the quasi-simultaneous detection of fragment ions. As a consequence, a complex mix of metabolites can be analyzed in a single run, no precursor ion is selected for individual fragmentation, and fragment ions can be related to their precursors using a mass fragmentation software and high-resolution mass databanks. This technique, coupled with UV analysis, was already applied with success to the identification and quantification of major carotenoids and chlorophylls in *Dunaliella salina* [30].

In the present study, we intended to develop a simple and fast UPLC-MS^E^ method for the identification of major phytoplankton pigments and the rapid discrimination of peaks corresponding to minor unidentified metabolites that could correspond to pigments or derivatives. We first established a UPLC-MS^E^ database with 22 standard pigments, representative of major phytoplankton taxa, to record their retention time and the accurate masses of parent and most intense fragment ions. This database was used to confirm the presence or absence of these pigments in a *Pp* ethanol extract. Ions whose accurate masses and fragmentation patterns did not match with the database were compared to mass data in the literature, to determine if they could correspond to previously reported metabolites, including pigments and pigment derivatives. 

## 2. Results and Discussion

### 2.1. UPLC-MS^E^ of Standard Pigments

Table 1 presents the retention times (Rt) and the theoretical and experimental high-resolution masses of parent and most intense fragment ions for the 22 pigment standards. The MS spectra of parent and fragment ions are presented as Supplementary Figure S1 to this paper. The mass errors between experimental and theoretical values were lower than 5 ppm for all pigments, demonstrating the reliability of the identification. The selected UPLC conditions allowed for a separation of all pigments except for chlorophyll *b* and DV-chlorophyll *b*, which exhibited the same Rt (4.71 min, Table 1) but could be easily discriminated by their accurate masses and fragmentation patterns (Table 1 and Supplementary Figure S1). Injection of the chl *a*, pheo *a*, and zea standards gave two peaks at 5.28 and 5.48, 7.43 and 7.88, and 4.04 and 4.47 min, respectively; these were interpreted as the presence of a mix of isomers/allomers in the standards. The absence of lutein in the zeaxanthin standard was confirmed by the absence of detection of the major fragment of lutein at *m/z* = 551.4253 [27,28,29]. UV-vis spectral analysis of the zea standard in acetone indicated maximal absorption wavelengths at 454.8 and 481.6 nm, with a % III:II band ratio of 32%. No peaks were detected at 450 and 474 nm, the maximal absorption wavelengths of (9-*cis*)-zea, or 446 and 472 nm, the maximal absorption wavelengths of (13-*cis*)-zea (as measured in an HPLC elution solvent containing hexane, dichloromethane, methanol, and *N,N*-diisopropylethylamine [31]). We thus concluded that the two peaks corresponded to the *all*-trans isomers of zea [31], namely (*all*-trans,3*R*,3′*R*)-zea and (*all*-trans,3*R*,3′*S*,meso)-zea [31].

**Table 1:** UPLC-MS^E^ of commercially available standard pigments. The theoretical and experimental masses of parent and major fragment ions, obtained in low and high collision modes, respectively, are presented. The values in brackets indicate the mass error (ppm) between experimental and theoretical values. * Na adduct, ** H adduct.

Standard pigment	Formula	Rt (min)	Function 1 (Low Energy)						Function 2 (High Energy)					
			Theoretical *m/z*			Experimental *m/z*			Theoretical *m/z*			Experimental *m/z*		
			M^●+^	[M + H]^+^	[M + Na]^+^	M^●+^	[M + H]^+^	[M + Na]^+^	fragments			fragments		
19-Butanoyl-fucoxanthin	C_46_H_64_O_8_	3.41	-	-	767.4499	-	-	767.4491 **(1.04 ppm)**	679.3999	-	-	679.3990 ^*^ **(1.32 ppm)**	-	-
19-Hexanoyl-fucoxanthin	C_48_H_68_O_8_	3.61	-	773.4992	795.4812	-	773.4973 **(2.46 ppm)**	795.4819 **(0.88 ppm)**	685.4468	679.3999	-	685.4451 ^*^ **(2.48 ppm)**	679.3978 ^*^ **(3.09 ppm)**	-
Alloxanthin	C_40_H_52_O_2_	3.95	564.3967	-	-	564.3965 **(0.35 ppm)**	-	-	549.3733	-	-	549.3713 **(3.64 ppm)**	-	-
Astaxanthin	C_40_H_52_O_4_	3.75	596.3866	-	619.3763	596.3862 **(0.67 ppm)**	-	619.3762 **(0.16 ppm)**	-	-	-	-	-	-
β-Carotene	C_40_H_56_	7.23	536.4382	-	-	536.4389 **(1.30 ppm)**	-	-	444.3756	-	-	444.3755 **(0.23 ppm)**	-	-
Cryptoxanthin	C_40_H_56_O	5.16	552.4331	-	-	552.4329 **(0.36 ppm)**	-	-	460.3705	-	-	460.3702 **(0.65 ppm)**	-	-
Chlorophyll *a*	C_55_H_72_O_5_N_4_Mg	5.28	892.5353	-	915.5251	892.5354 **(0.11 ppm)**	-	915.5250 **(0.11 ppm)**	614.2380	481.1879	-	614.2387 **(1.14 ppm)**	481.1883 **(0.83 ppm)**	-
Chlorophyll *a*	C_55_H_72_O_5_N_4_Mg	5.48	892.5353	-	-	892.5349 **(0.45 ppm)**	-	-	614.2380	481.1879	-	614.2380 **(0 ppm)**	481.1901 **(4.57 ppm)**	-
Chlorophyll *b*	C_55_H_70_O_6_N_4_Mg	4.71	906.5146	-	929.5043	906.5149 **(0.33 ppm)**	-	929.5046 **(0.32 ppm)**	628.2172	495.1671	-	628.2184 **(1.91 ppm)**	495.1682 **(2.22 ppm)**	-
Chlorophyll *c*2	C_35_H_28_O_5_N_4_Mg	3.62	-	609.1988	631.1808	-	609.1984 **(0.66 ppm)**	631.1812 **(0.63 ppm)**	549.1777	-	-	549.1765^**^ **(2.19 ppm)**	-	-
Chlorophyllide *a*	C_35_H_34_O_5_N_4_Mg	3.19	614.2380	-	-	614.2378 **(0.33 ppm)**	-	-	582.2117	481.1879	-	582.2095 **(3.78 ppm)**	481.1878 **(0.21 ppm)**	-
Diadinoxanthin	C_40_H_54_O_3_	3.80	582.4073	-	605.3971	582.4077 **(0.69 ppm)**	-	605.3985 **(2.31 ppm)**	-	-	-	-	-	-
Diatoxanthin	C_40_H_54_O_2_	4,00	566.4124	-	-	566.4132 **(1.41 ppm)**	-	-	119.0861	-	-	119.0862 **(0.84 ppm)**	-	-
DV chlorophyll *a*	C_55_H_70_O_5_N_4_Mg	5.25	890.5197	-	-	890.5191 **(0.67 ppm)**	-	-	612.2223	-	-	612.2227 **(0.65 ppm)**	-	-
DV chlorophyll *b*	C_55_H_68_O_6_N_4_Mg	4.71	904.4989	-	-	904.4988 **(0.11 ppm)**	-	-	626.2025	-	-	626.2015 **(1.60 ppm)**	-	-
Fucoxanthin	C_42_H_58_O_6_	3.48	-	-	681.4131	-	-	681.4130 **(0.15 ppm)**	527.3161	-	-	527.3148 **(2.47 ppm)**	-	-
Peridinin	C_39_H_50_O_7_	3.15	630.3557	-	653.3454	630.3540 **(2.7 ppm)**	-	653.3458 **(0.61 ppm)**	635.3349	593.3243	575.3137	635.3348 ^*^ **(0.16 ppm)**	593.3247 ^*^ **(0.67 ppm)**	575.3130 ^*^ **(1.22 ppm)**
Pheophorbide *a*	C_35_H_36_O_5_N_4_	3.98	-	593.2764	-	-	593.2769 **(0.84 ppm)**	-	533.2553	-	-	533.2557 ^**^ **(0.75 ppm)**	-	-
Pheophytin *a*	C_55_H_74_O_5_N_4_	7.43	-	871.5737	893.5557	-	871.5734 **(0.34 ppm)**	893.5560 **(0.34 ppm)**	593.2764	533.2553	-	593.2768 ^**^ **(0.67 ppm)**	533.2554 ^**^ **(0.19 ppm)**	-
Pheophytin *a*	C_55_H_74_O_5_N_4_	7.88	-	871.5737	893.5557	-	871.5739 **(0.23 ppm)**	893.5555 **(0.22 ppm)**	593.2764	-	-	593.2768 ^**^ **(0.67 ppm)**	-	-
Prasinoxanthin	C_40_H_56_O_4_	3.65	600.4179	-	623.4076	600.4178 **(0.17 ppm)**	-	623.4077 **(0.16 ppm)**	469.3107	-	-	469.3098 **(1.92 ppm)**	-	-
Pyropheophorbide *a*	C_33_H_34_O_3_N_4_	4.34	-	535.2709	-	-	535.2714 **(0.93 ppm)**	-	461.2341	447.2185	-	461.2341 ^**^ **(0 ppm)**	447.2191 ^**^ **(1.34 ppm)**	-
Violaxanthin	C_40_H_56_O_4_	3.64	600.4179	-	623.4076	600.4173 **(1.00 ppm)**	-	623.4077 **(0.16 ppm)**	469.3083	221.1542	-	469.3082 ^*^ **(0.21 ppm)**	221.1544 **(0.90 ppm)**	-
Zeaxanthin	C_40_H_56_O_2_	4.04	568.4280	-	591.4178	568.4273 **(1.23 ppm)**	-	591.4186 **(1.35 ppm)**	476.3654	-	-	476.3651 **(0.63 ppm)**	-	-
Zeaxanthin	C_40_H_56_O_3_	4.47	568.4280	-	-	568.4293 **(2.29 ppm)**	-	-	476.3654	-	-	476.3664 **(2.10 ppm)**	-	-

### 2.2. Identification of Pp Pigments Using the Standard Pigments Database

**Figure 1:**
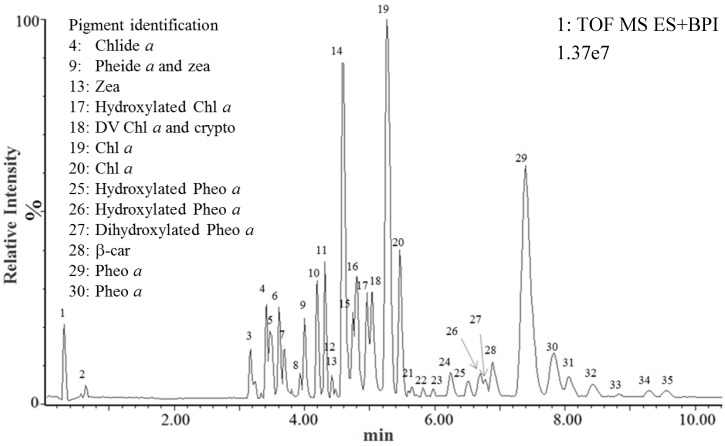
UPLC-MS^E^ chromatogram of *Pp* ethanol extract. Thirty-five peaks were discriminated and annotated according to their retention times. Each peak corresponded to one or several ions formed in the MS source and detected by the MS detector in function 1 (parent ions).

The UPLC-MS^E^ analysis of *Pp* ethanol extract was achieved in a single 13-min run, and an UPLC-MS^E^ chromatogram in which 35 peaks were discriminated was obtained (Figure 1 and Table 2). 

**Figure 2:**
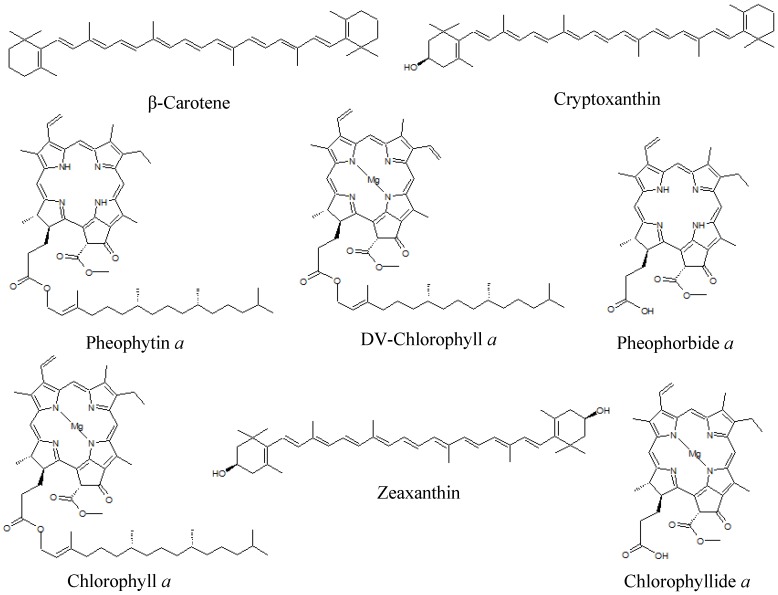
Chemical structures of pigments identified in the *Pp* ethanol extract.

Comparison of the Rt and mass data with the standard pigments databank allowed the unequivocal identification of eight pigments or derivatives (Table 2 and Figure 2): β-car, chl *a*, chlide *a*, crypto, zea, pheide *a*, phein *a*, and DV chl *a*. With the exception of DV chl *a*, all the detected pigments had previously been reported in *Pp* [32,33]. The MS^E^ analysis also excluded the presence of 14 pigments in the *Pp* ethanol extract (19′ But-fuco, 19′ hexan-fuco, allo, asta, chl *b*, chl *c*2, diadino, diato, DV-chl *b*, fuco, perid, prasino, pyropheide *a*, and viola). Chl *a* and β-car are ubiquitous pigments present in all phytoplankton species. Crypto is the immediate precursor of zea, which is a major carotenoid in rhodophytes. Chlide *a* is the immediate biosynthetic precursor of chl *a*, and may also correspond to a degradation intermediate of chl *a*. Pheide *a* and phein *a* are chlorophyll *a* transformation products, absent from *Pp* living cells and reflecting chl *a* degradation during the pigment extraction process [12].

**Table 2:** Pigments identified in the *Pp* ethanol extract after UPLC-MS^E^ and comparison with the standard pigments database using the Chromalynx software. ** H adduct.

Peak	Pigment identification	Formula	Rt (min)	Function 1 (Low energy)						Function 2 (High energy)			
				Theoretical *m/z*			Experimental *m/z*			Theoretical *m/z*		Experimental *m/z*	
				M^●+^	[M + H]^+^	[M + Na]^+^	M^●+^	[M + H]^+^	[M + Na]^+^	fragments		fragments	
4	Chlorophyllide *a*	C_35_H_34_O_5_N_4_Mg	3.40	614.2380	-	-	614.2380 **(0 ppm)**	-	-	481.1879	-	481.1880 **(0.21 ppm)**	-
9	Pheophorbide *a*	C_35_H_36_N_4_O_5_	4.00	-	593.2764	615.2583	-	593.2783 **(3.2 ppm)**	615.2594 **(1.79 ppm)**	533.2553	-	533.2561^**^ **(1.50 ppm)**	-
9	Zeaxanthin	C_40_H_56_O_2_	4.06	568.4280	-	-	568.4275 **(0.88 ppm)**	-	-	476.3654	-	476.3635 **(3.99 ppm)**	-
13	Zeaxanthin	C_40_H_56_O_2_	4.47	568.4280	-	-	568.4282 **(0.35 ppm)**	-	-	476.3654	-	476.3652 **(1.89 ppm)**	-
18	DV-Chlorphyll *a*	C_55_H_70_O_5_N_4_Mg	5.09	890.5197	-	-	890.5206 **(1.01 ppm)**	-	-	612.2223	-	612.2216 **(1.01 ppm)**	-
18	Cryptoxanthin	C_40_H_56_O	5.17	552.4331	-	-	552.4346 **(2.72 ppm)**	-	-	460.3705	-	460.3695 **(2.17 ppm)**	-
19	Chlorophyll *a*	C_55_H_72_O_5_N_4_Mg	5.26	892.5353	-	-	892.5368 **(1.68 ppm)**	-	-	614.2380	481.1879	614.2390 **(1.63 ppm)**	481.1879 **(0 ppm)**
20	Chlorophyll *a*	C_55_H_72_O_5_N_4_Mg	5.46	892.5353	-	-	892.5389 **(4.03 ppm)**	-	-	614.2380	481.1879	614.2406 **(4.23 ppm)**	481.1891 **(2.49 ppm)**
28	β-Carotene	C_40_H_56_	7.18	536.4382	-	-	536.4384 **(0.37 ppm)**	-	-	444.3756	-	444.3752 **(0.9 ppm)**	-
29	Pheophytin *a*	C_55_H_74_O_5_N_4_	7.40	-	871.5737	893.5557	-	871.5723 **(1.61 ppm)**	893.5549 **(0.9 ppm)**	593.2768	533.2554	593.2755 ^**^ **(2.19 ppm)**	533.2550^**^ **(0.75 ppm)**
30	Pheophytin *a*	C_55_H_74_O_5_N_4_	7.83	-	871.5737	893.5557	-	871.5741 **(0.46 ppm)**	893.5568 **(1.23 ppm)**	593.2768	533.2554	593.2770 ^**^ **(0.34 ppm)**	533.2556^**^ **(0.38 ppm)**

**Figure 3:**
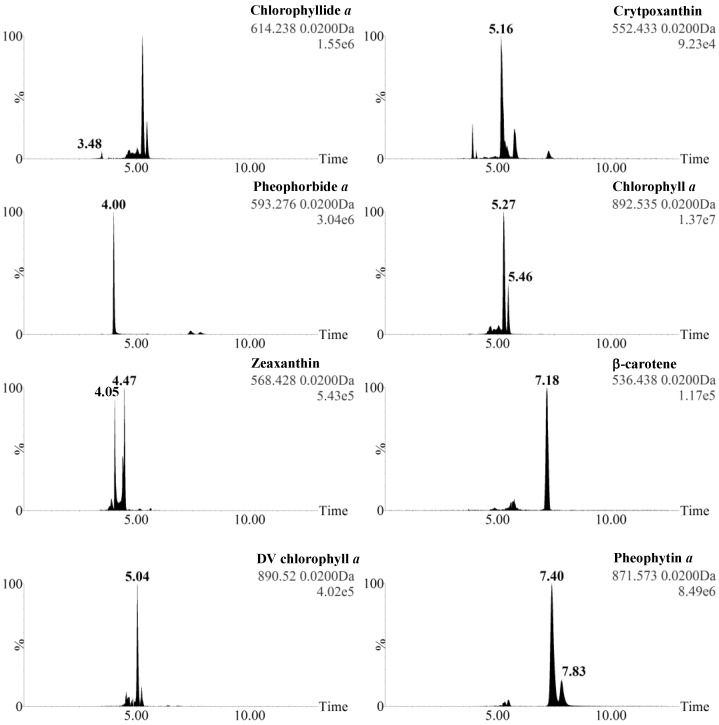
Extracted masses of the eight pigments isolated from the UPLC-MS^E^ chromatogram of *Pp* ethanol extract in function 1 (low collision energy mode, parent ions).

In the case of Zea, Chl *a*, and Phein *a*, the detection of two ions was explained by the mix of isomers/allomers with different UPLC retention times in the standard. Some ions were detected at several retention times because they simultaneously corresponded to parent ions (ionized unfragmented molecules) and fragment ions released from other parent ions (e.g., Chlide *a* is detected at 3.48 min (parent ion of Chlide *a*) and 5.27 and 5.46 min (fragment ion of Chl *a* isomers) (Figure 3).

Surprisingly, the MS^E^ analysis unequivocally identified DV chl *a* in *Pp* (peak 18). The DV chl *a* parent ion peak surface was minority compared to chl *a* (2.50% ± 0.19%, Figure 3), but this ion was systematically detected in iterative extractions. Until now, DV chl *a* had only been described in prochlorophytes (e.g., *Prochlorococcus*), for which it is used as a chemotaxonomic tracer [34,35,36]. We considered the possibility that its presence in *Pp* may be explained by the transformation of chl *a* in the mass spectrometer source, but excluded this possibility as no ion corresponding to DV chl *a* was detected when standard chl *a* was subjected to UPLC-MS^E^ (Table 1). We also considered the possibility that DV chl *a* could be formed by the thermal or chemical transformation of chl *a* in ethanol. To test this hypothesis, standard chl *a* was subjected to the ethanolic extraction process and the resulting extract was analyzed by UPLC-MS^E^. No ion corresponding to DV chl *a* was detected, demonstrating that DV chl *a* was not produced by the contact of chl *a* with ethanol during the extraction process. We thus concluded that DV chl *a* was produced by the contact of a *Pp* pigment with enzymes or metabolites released during the extraction process, or much less probably that it was effectively present in living *Pp* cells. A culture contamination by prochlorophytes was excluded because no ions corresponding to DV-chl *b* were detected. According to the tiny amounts detected in *Pp*, DV chl *a* can still be considered as a relevant chemotaxonomic marker for prochlorophytes, as its high concentration in pigment extracts cannot signify the presence of *Pp*. Our MS^E^ analysis, however, demonstrates that its minor presence in extracts from other phytoplankton taxa should not be excluded.

### 2.3. Identification of Pp Pigments Derivatives Using the Metabolynx Software

The Metabolynx software was used to determine if unidentified peaks in the UPLC-MS^E^ chromatogram exhibited accurate masses corresponding to parent ions of pigments metabolites. The masses of the major parent ions detected as peaks 17, 25, 26, and 27 exactly matched with that of hydroxylated chl *a* and phein *a* (Table 3). Analysis of fragment ions detected at the same Rt confirmed this identification (Table 3). The presence of hydroxylated derivatives of chl *a* and phein *a* in the *Pp* ethanol extract suggested a possible hydroxylation of both pigments during the ethanolic extraction, or less probably the presence of both hydroxylated pigments in living *Pp* cells. Chl *a* and phein *a* standards were subjected to the ethanolic extraction and their respective hydroxylated derivatives were detected using the UPLC-MS^E^ analysis (data not shown), confirming that the hydroxylation of both pigments occurred during the extraction.

**Table 3:** UPLC-MS^E^ identification of *Pp* pigments metabolites using the Metabolynx software. ** H adduct.

Peak	Pigment identification	Formula	Rt (min)	Function 1 (Low energy)						Function 2 (High energy)			
				Theoretical *m/z*			Experimental *m/z*			Theoretical *m/z*		Experimental *m/z*	
				M^●+^	[M + H]^+^	[M + Na]^+^	M^●+^	[M + H]^+^	[M + Na]^+^	fragments		fragments	
17	Hydroxylated chlorophyll *a*	C_55_H_72_O_6_N_4_Mg	4.97	908.5302	-	-	908.5333 **(3.41 ppm)**	-	-	630.2329	-	614.2340 **(1.75 ppm)**	-
25	Hydroxylated pheophytin *a*	C_55_H_74_O_6_N_4_	6.55	-	887.5687	909.5506	-	887.5695 **(0.9 ppm)**	909.5506 **(0 ppm)**	609.2713	549.2502	609.2710 ^**^ **(0.49 ppm)**	549.2491 ^**^ **(2.0 ppm)**
26	Hydroxylated pheophytin *a*	C_55_H_74_O_6_N_4_	6.70	-	887.5687	909.5506	-	887.5676 **(1.24 ppm)**	909.5495 **(1.21 ppm)**	609.2713	549.2502	609.2705 ^**^ **(1.31 ppm)**	549.248 ^**^ **(4.01 ppm)**
27	Dihydroxylated pheophytin *a*	C_55_H_74_O_7_N_4_	6.80	-	903.5636	-	-	903.5627 **(1.00 ppm)**	-	625.2662	565.2451	625.2655 ^**^ **(1.12 ppm)**	565.2436 ^**^ **(2.65 ppm)**

### 2.4. Tentative Identification of Other Metabolites in the Pp Ethanol Extract

Because of the high number of ionizable metabolites extracted by ethanol, numerous ions that did not correspond to pigments or derivatives were detected in the UPLC-MS^E^ chromatogram, some of them co-eluting with parent ions corresponding to pigments. For each unidentified parent ion, the most probable molecular formula was determined using the Masslynx software. A bibliographic analysis was then performed to determine if these structures, their masses and fragmentation pattern had previously been reported. The result of this study indicated that the detected metabolites most probably corresponded to lipids, glycolipids, plastoquinone, gracilamide B, erucamide, and phthalates (Table 4, Supplementary Figure S2, [37,38,39,40,41]). The detection of ions that may correspond to plastoquinone and various galactolipids was in agreement with the hypothesis that in addition to pigments, ethanol solubilized various molecules present in the thylakoid membrane of *Pp.* The digalactosyldiacylglycerols detected using our UPLC-MS^E^ analysis corresponded to glycolipids previously reported in microalgae [37,38,39,40], corroborating their possible presence in *Pp* extract. Gracillamide B was previously reported in the red seaweed *Gracillaria asiatica* [41], but to our knowledge this is the first report of this molecule in a red phytoplankton species. Its presence in *Pp*, if it is confirmed by additional structural analysis, could demonstrate the common share of its biosynthetic pathways between red macro and microalgae. Erucamide and phtalates are described as plastic vial and UPLC contaminants and their traces were easily detected by the MS^E^ analysis. 

## 3. Experimental Section 

### 3.1. Chemicals

Standard pigment solutions were purchased as ethanol or acetone solutions. Diadino, diato and viola standard solutions (0.6 to 1.5 mg·L^−1^, unknown purity) were obtained from DHI Lab Denmark. 19′ but-fuco, 19′ hexan-fuco, allo, asta, chlide *a*, chl *c*2, DV chl *a*, DV chl *b*, fuco, perid, phein *a*, and prasino were obtained as a mix solution from DHI Lab Denmark (DHI mix, containing the pigments obtained from three phytoplankton species representative of rhodophytes, chlorophytes, and haptophytes). The species used to prepare the DHI mix are confidential, and the concentration and purity of individual pigments in the DHI mix are unknown. Standard chl *a* (purity 95%), chl *b* (purity 95%), zea (purity 95%), β-car (purity 97%), and crypto (purity 97%) were purchased from Sigma-Aldrich, France. Pyropheide *a* (purity 95%) and pheide *a* (purity 95%) were purchased from Frontier Scientific, Frankfurt am Main, Germany. Ultra-pure water was obtained using a Milli-Q system (Millipore, Molsheim, France). Reagents were of HPLC grade for extraction and ULC-MS grade (Biosolve, Valkenswaard, Netherlands) for the MS^E^ analysis. 

**Table 4:** Putative nature of non-pigment metabolites present in the *Pp* ethanol extract according to the comparison of MS^E^ data, mass data from the literature [37,38,39,40,41], and most probable formula proposed by the Mass Fragment software.

Peak	Rt (min)	Major ions ( *m/z*)		Empirical formula		Tentative identification		Mass error (ppm)	
		1	2	1	2	1	2	1	2
1	0.32	277.0908	-	C_9_H_18_O_8_Na	-	C_9_H_18_O_8_Na [M + Na]^+^	-	3.25	-
2	0.64	376.2599	398.2419	C_21_H_34_N_3_O_3_	C_21_H_33_N_3_O_3_Na	C_21_H_34_N_3_O_3_ [M + H]^+^	C_21_H_34_N_3_O_3_Na [M + Na]^+^	0.27	0.25
3	3.17	377.2693	399.2515	C_23_H_37_O_4_	C_23_H_36_O_4_Na	Butylundecylphthalate [M + H]^+^	Butylundecylphthalate [M + Na]^+^	0.27	1.00
4	3.42	379.2845	401.2672	C_23_H_39_O_4_	C_23_H_38_O_4_Na	2-Arachidonoylglycerol [M + H]^+a^	2-Arachidonoylglycerol [M + Na]^+a^	0.79	1.00
5	3.46	303.2329	325.2136	C_20_H_31_O_2_	C_20_H_30_O_2_Na	Eicosapentenoic acid [M + H]^+a^	Eicosapentenoic acid [M + Na]^+a^	1.65	2.15
6	3.61	353.2668	-	C_17_H_33_N_6_O_2_	-	C_17_H_32_N_6_O_2_ [M + H]^+^	-	0.85	-
7	3.68	305.2482	327.2288	C_20_H_33_O_2_	C_20_H_32_O_2_Na	Arachidonoic acid [M + H]^+^^a^	Arachidonoic acid [M + Na]^+a^	0.33	3.67
8	3.80	413.2671	-	C_24_H_38_O_4_Na	-	Diisooctyl phthalate [M + H]^+^^b^	-	2.18	-
10	4.20	338.3425	360.3244	C_22_H_44_NO	C_22_H_43_NONa	Erucamide [M + H]^+^^b^	Erucamide [M+Na]^+b^	0.59	0.56
11	4.31	1007.5737	-	C_55_H_84_O_15_Na	-	Digalactosyldiacylglycerol [M + Na]^+^ (40:5) [37,38,39,40]	-	2.88	-
12	4.42	1009.5861	-	C_55_H_86_O_15_Na	-	Digalactosyldiacylglycerol [M + Na]^+^ (40:9) [37,38,39,40]	-	0.30	-
13	4.47	947.5701	-	C_50_H_84_O_15_Na	-	Digalactosyldiacylglycerol [M + Na]^+^ (35:5) [37,38,39,40]	-	0.74	-
14	4.61	961.5868	-	C_51_H_86_O_15_Na	-	Digalactosyldiacylglycerol [M + Na]^+^ (36:5) [37,38,39,40]	-	0.42	-
15	4.74	963.6005	-	C_51_H_88_O_15_Na	-	Digalactosyldiacylglycerol [M + Na]^+^ (36:4) [37,38,39,40]	-	1.66	-
16	4.80	685.4784	939.5999	C_39_H_62_N_6_O_3_Na	C_49_H_88_O_15_Na	C_39_H_62_N_6_O_3_Na [M + Na]^+^	Digalactosyldiacylglycerol [M + Na]^+^ (34:3) [37,38,39,40]	0.44	2.34
17	4.97	687.4990	-	C_43_H_68_O_5_Na	-	Diacylglycerol [M + Na]^+^ (40:8)^a^ [38,39]	-	3.78	-
18	5.03	637.4825	-	C_39_H_66_O_5_Na	-	Diacylglycerol [M + Na]^+^ (40:8)^a^ [38,39]	-	2.67	-
21	5.64	591.4996	-	C_37_H_67_O_5_	-	Diacylglycerol [M + H]^+^ (34:3)^a^ [38,39]	-	1.18	-
22	5.82	726.6608	748.6443	C_44_H_88_NO_6_	C_44_H_87_NO_6_Na	Gracilamide B (+ OH -H + CH_2_) [M + H]^+^ [41]	Gracilamide B (+ OH -H + CH_2_) [M+Na]^+^ [41]	0.69	1.60
23	5.98	696.6515	718.6335	C_43_H_86_NO_5_	C_43_H_85_NO_5_Na	Gracilamide B [M + H]^+^ [41]	Gracilamide B [M+Na]^+^ [41]	1.29	1.39
24	6.24	710.6680	732.6506	C_44_H_88_NO_5_	C_44_H_87_NO_5_Na	Gracilamide B (+ CH_2_) [M + H]^+^ [41]	Gracilamide B (+ CH2) [M + Na]^+^ [41]	2.53	3.28
28	6.87	967.6772	-	C_63_H_92_O_6_Na	-	Triacylglycerol [M + Na]^+^ (60:15)^a^ [39]	-	2.07	-
31	8.04	971.7145	-	C_63_H_96_O_6_Na	-	Triacylglycerol [M + Na]^+^ (60:13)^a^ [39]	-	4.12	-
32	8.44	921.6916	-	C_59_H_94_O_6_Na	-	Triacylglycerol [M + Na]^+^ (56:10) ^a^ [39]	-	3.47	-
33	8.81	973.7232	-	C_63_H_98_O_6_Na	-	Triacylglycerol [M + Na]^+^ (60:12) ^a^ [39]	-	2.98	-
34	9.29	923.7145	-	C_59_H_96_O_6_Na	-	Triacylglycerol [M + Na]^+^ (56:9) ^a^ [39]	-	4.33	-
35	9.52	771.6071	-	C_53_H_80_O_2_Na	-	Plastoquinone [M + Na]^+^	-	1.94	-

^a^ Lipid Data Bank; ^b^ Background ion list Waters.

### 3.2. Microalgae

*Porphyridium purpureum* (*Pp*) CCAP 1380.3 (Rhodophyte, Bangiophyceae), was grown at 120 μmol·m^−2^·s^−1^ irradiance in four units of 50-L column photobioreactors (designed by the PBA Ifremer Laboratory, Nantes, France) with 35% salinity seawater enriched by Walne medium [42]. Batch cultures were maintained at 20 °C under continuous light provided by fluorescent lamps (Philips TLD 58W 865) and bubbled with 0.22 μm filtered air containing 3% (*v/v*) CO_2_. Microalgae were harvested after 12–16 days of growth and separated from the culture medium by a two-step process. The first step used a clarifier separator (Clara 20, Alfa Laval Corporate AB, Lund, Sweden) at 100 L·h^−1^, 9000× *g*, room temperature. Step two used a soft centrifugation at 4000× *g*, 20 min, 4 °C to separate the slurry. Algal paste was freeze-dried at −55 °C and *P* < 1 hPa, on a freeze-dryer equipped with a HetoLyoPro 3000 condenser and a Heto cooling trap (Thermo, Villebon sur Yvette, France).

### 3.3. Pigment Extraction

Phycobiliproteins were removed by maceration of freeze-dried microalgae in water (1:100 *w/v*, 4 h, magnetic stirring). The extract was centrifuged (11,000× *g*, 10 min) and the supernatant was discarded. Chlorophylls and carotenoids present in the pellet were extracted by a 4-h maceration at room temperature in ethanol (1:100 *w/v*, magnetic stirring). The extract was centrifuged (11,000× *g*, 10 min) and the supernatant was dried using a Büchi rotavapor (Büchi, France). The remaining extract was solubilized in 2 mL ethanol. Triplicate independent extracts were prepared and each one was analyzed three times by UPLC-MS^E^.

### 3.4. UPLC-MS^E^

#### 3.4.1. Equipment and Analytical Conditions

UPLC-MS^E^ analyses were performed using an Acquity UPLC H-Class (Waters, Milford, MA, USA) coupled to a Xevo G2 S Q-TOF (Waters, Manchester, United Kingdom) mass spectrometer equipped with an electrospray ionization (ESI) source (Waters, Manchester, United Kingdom). The chromatographic system consisted of a quaternary pump (Quaternary Solvent Manager, Waters) and an autosampler (Sample Manager-FTN, Waters) equipped with a 10 µL sample loop. The pigment standard solutions and ethanol extracts were diluted in methanol before injection for UPLC-MS^E^ (hundredth for standard pigment solutions containing a single pigment and *Pp* ethanol extracts, tenth for the DHI mix). Five microliters of methanolic solutions were injected into a Waters Acquity UPLC BEH C18 column (2.1 × 50 mm, 1.7 µm). The system was operated under the following gradient elution program: solution A (0.01% formic acid in H_2_O) in solution B (0.01% formic acid in MeOH) at a flow rate of 400 µL·min^−1^ as follows: 0–0.5 min, 70% B; 0.5–3.00 min, 70%–100% B; 3.00–11.00 min, 100% B; the eluent was adjusted to its initial composition in 2 min. The column and autosampler were maintained at 25 °C and 4 °C, respectively, and the column back pressure was 13,000 psi. Final ESI conditions were: source temperature 120 °C, desolvation temperature 500 °C, cone gas flow 50 L·h^−1^, desolvation gas flow 1000 L·h^−1^, capillary voltage 2.5 kV, sampling cone voltage 35 V, and source offset 80 V. The instrument was set to acquire over the *m/z* range 50–1200 with a scan time equal to 0.5 s. Data were collected in the positive (ESI^+^) electrospray ionization modes using the MS^E^ function in centroid mode, with a 6 V collision energy in function 1 (parent ions experiment) and a collision energy ramp of 20–40 V in function 2 (fragment ions experiment) (frequency of low to high collision switch = 30 Hz). Leucine Enkephalin (MW = 555.62 Da) (1 ng·µL^−1^) was used as the lock mass for mass shift correction. The mass spectrometer was calibrated before analyses using 0.5 mM sodium formate solution. The mass error between experimental and theoretical parent and fragment ions was calculated as (**|**experimental *m/z* − theoretical *m/z***|**/ theoretical *m/z*) × 10^6^ (ppm).

#### 3.4.2. Software

MS^E^ data were recorded in a centroid mode and analyzed using the MassLynx software. In a first step, standard pigments were subjected to MS^E^ analysis and a personal database including the empirical formula of observed ions, Rt, and most diagnostic fragments in function 2 was created using the Notepad text editor. The MassFragment software was used to identify and propose a structure for each pigment ion fragment. The *Pp* ethanol extract was then subjected to MS^E^ and data obtained (Rt, accurate masses of parent and fragment ions) were compared to the values recorded in the standard pigments database by the Chromalynx software, to confirm the presence or absence of pigments and metabolic derivatives. An additional manual check was performed for each compound identified by Chromalynx, using the Masslynx software. The Metabolynx XS software was used to define if some ions could sign the presence of pigment metabolites according to the possible phase 1 (oxidation, reduction, hydrolysis) and/or phase 2 (conjugation) biotransformations. 

## 4. Conclusions 

The UPLC-MS^E^ method allowed the rapid and unambiguous identification of phytoplankton pigments in an ethanolic extract, by comparison of the Rt and accurate masses of major and diagnostic parent and fragment ions recorded in a standard pigment database. The quasi-simultaneous detection of parent and fragment ions allowed us to retrace the fragmentation pattern of each pigment. MS^E^ data were, however, insufficient to identify the structure of pigment allomers or isomers, for which additional spectrophotometric data were necessary. Analysis of the unidentified ions using the Metabolynx XS software revealed that some of them corresponded to hydroxylated pigments metabolites produced during the ethanolic extraction. Additionally, UPLC-MS^E^ analysis revealed the presence in the *Pp* ethanolic extract of ions that could correspond to previously reported metabolites, including fatty acids, glycerides, galactosylglycerides, ceramides, and peptides. Beyond its high interest for pigment identification, UPLC-MS^E^ can theoretically be used to detect and identify any ionizable and fragmentable molecules, such as marine heterocycles, oligosaccharides, lipids, or proteins. It should thus be considered as a method of choice for the study of marine drugs and toxins.

## Supplementary Files

Supplementary File 1

## Abbreviations

19′ But-fuco: 19′ Butanoyloxy-fucoxanthin
19′ Hexan-fuco: 19′ Hexanoyloxy-fucoxanthin
Allo: Alloxanthin
Asta: Astaxanthin
Chl: Chlorophyll
Chlide *a*: Chlorophyllide *a*
Diadino: Diadinoxanthin
Diato: Diatoxanthin
DV chl: Divinylchlorophyll
Fuco: Fucoxanthin
HPLC: High Performance Liquid Chromatography
MS^E^: High-resolution mass spectrometry with simultaneous acquisition of accurate mass at high and low collision energy
Perid: Peridinin
Pheide *a*: Pheophorbide *a*
Phein *a*: Pheophytin *a*
Pp: *Porphyridium purpureum*
Prasino: Prasinoxanthin
Pyropheide *a*: Pyropheophorbide *a*
UPLC: Ultra-Performance Liquid Chromatography
UV-vis: Ultraviolet-Visible
Viola: Violaxanthin
Zea: zeaxanthin
β-car: β,β-carotene
crypto: cryptoxanthin
