# Supplementary material for: UPLC-MSE Profiling of Phytoplankton Metabolites: Application to the Identification of Pigments and Structural Analysis of Metabolites in Porphyridium purpureum
Source: Mar Drugs. 2015 Apr 22;13(4):2541–58. doi: 10.3390/md13042541 (PMC4413225; doi:10.3390/md13042541)
Supplement: Supplementary File 1 [file marinedrugs-13-02541-s001.pdf]

# Supplementary Information

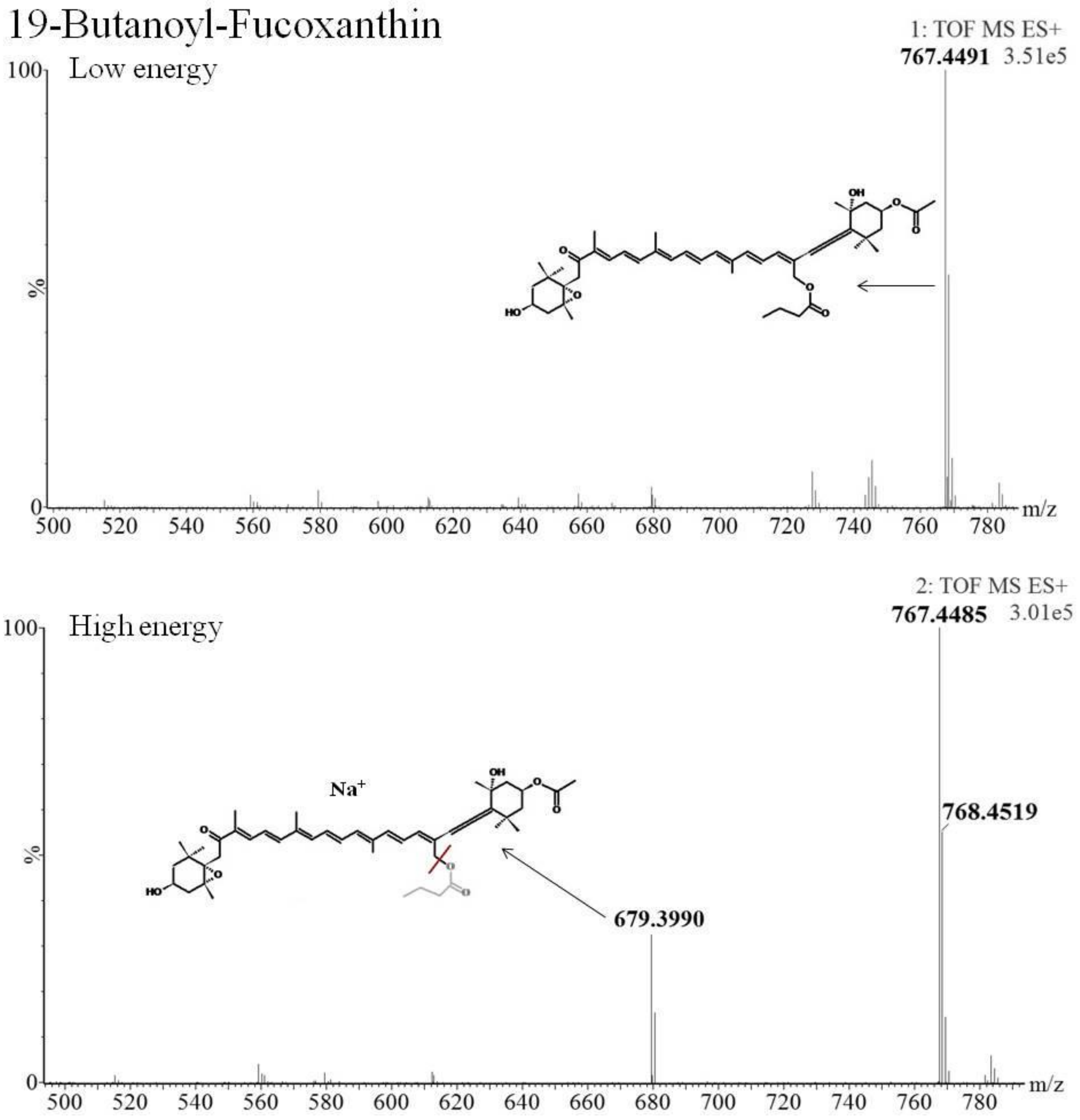


**Figure S1.** *Cont.*

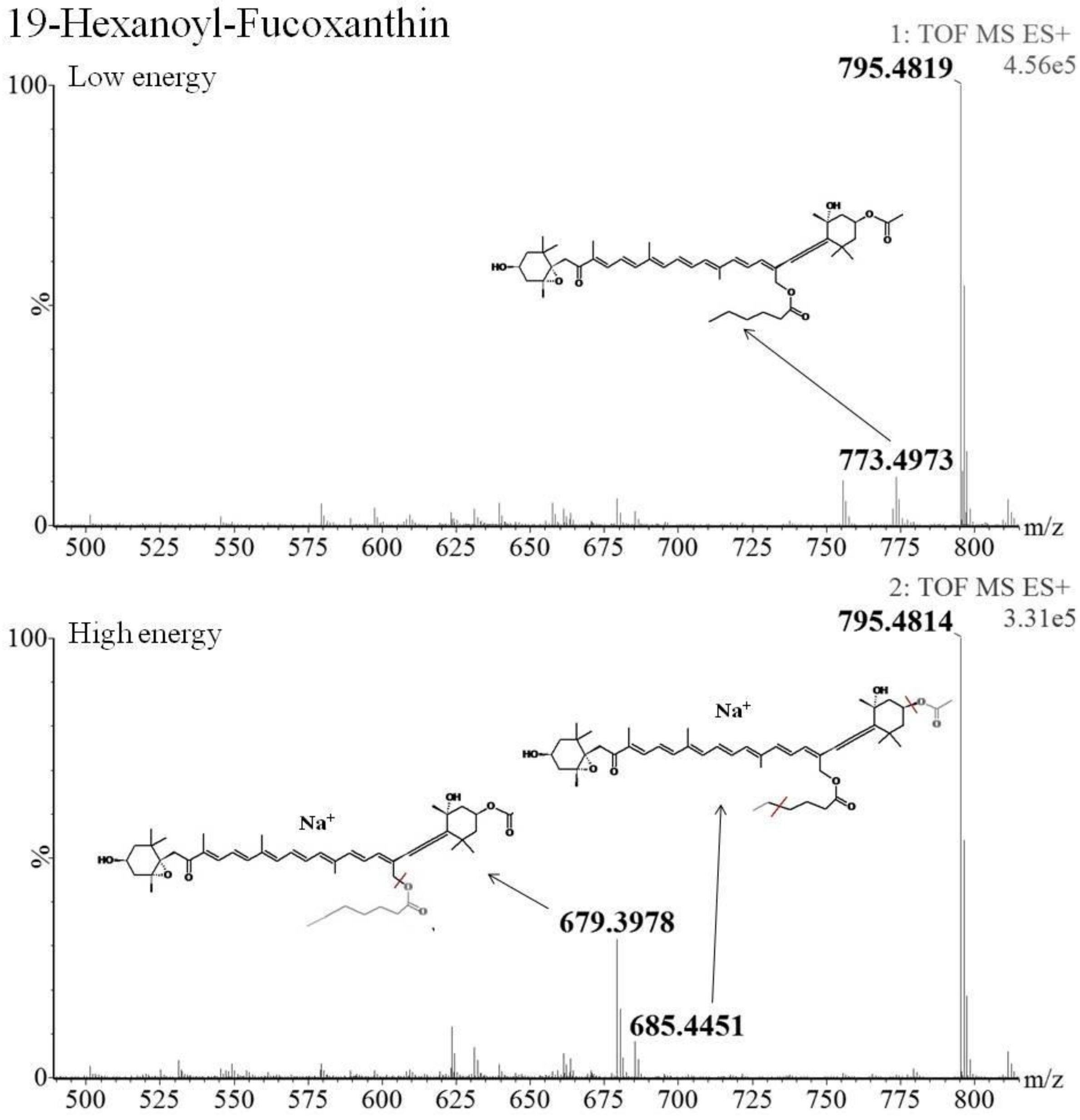


**Figure S1.** *Cont*.

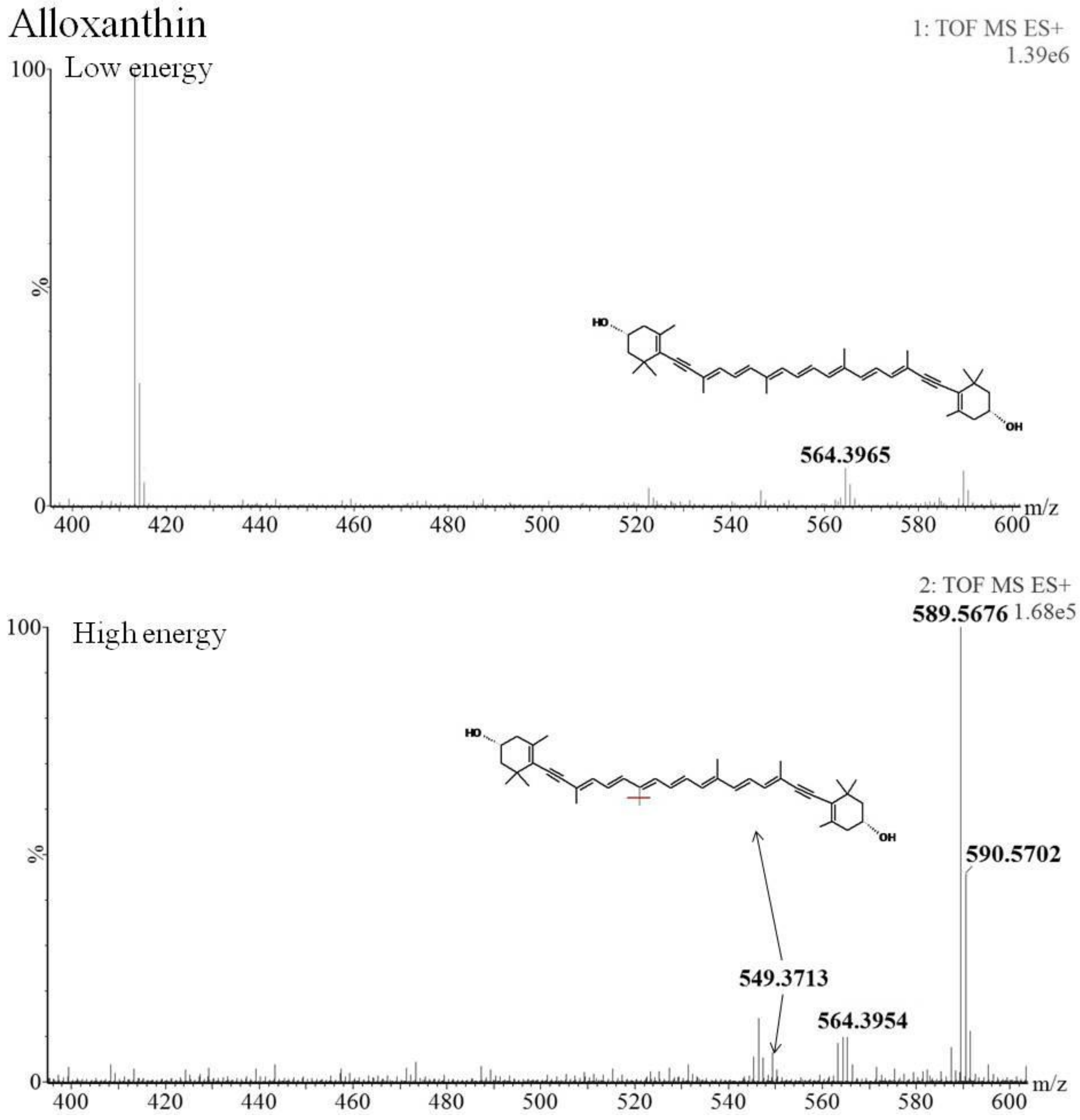


**Figure S1.** *Cont*.

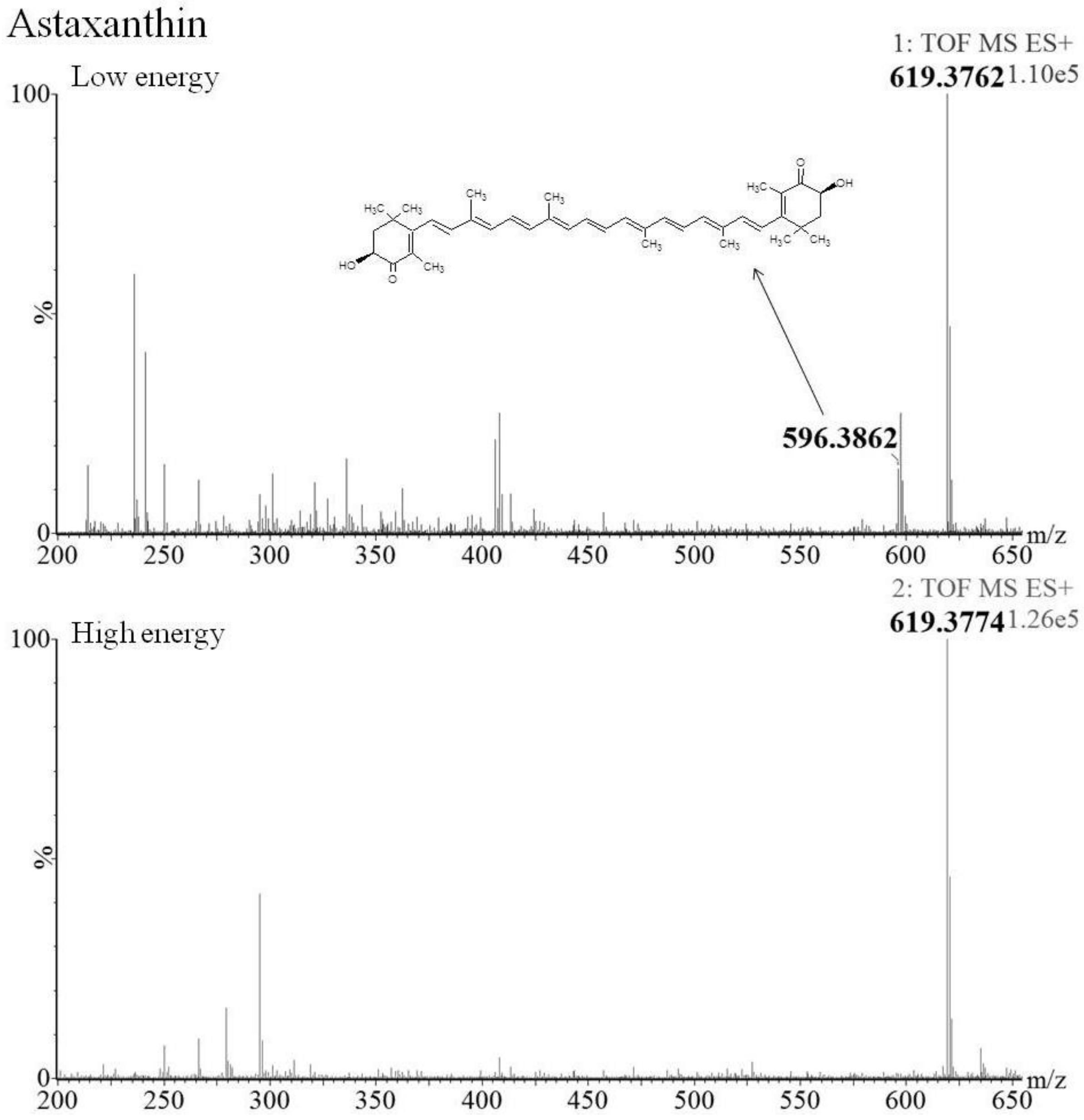


**Figure S1.** *Cont*.

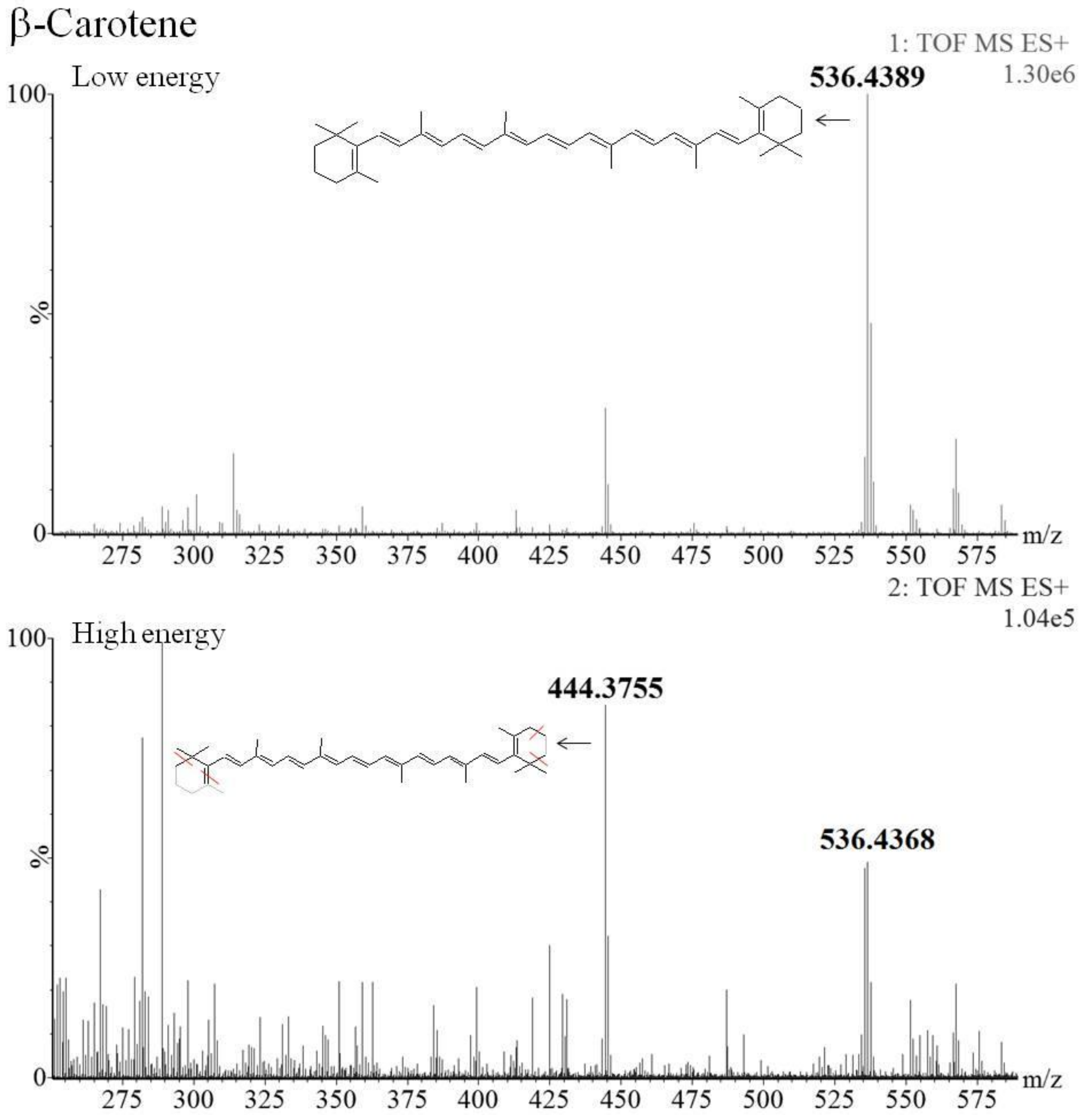


**Figure S1.** *Cont.*

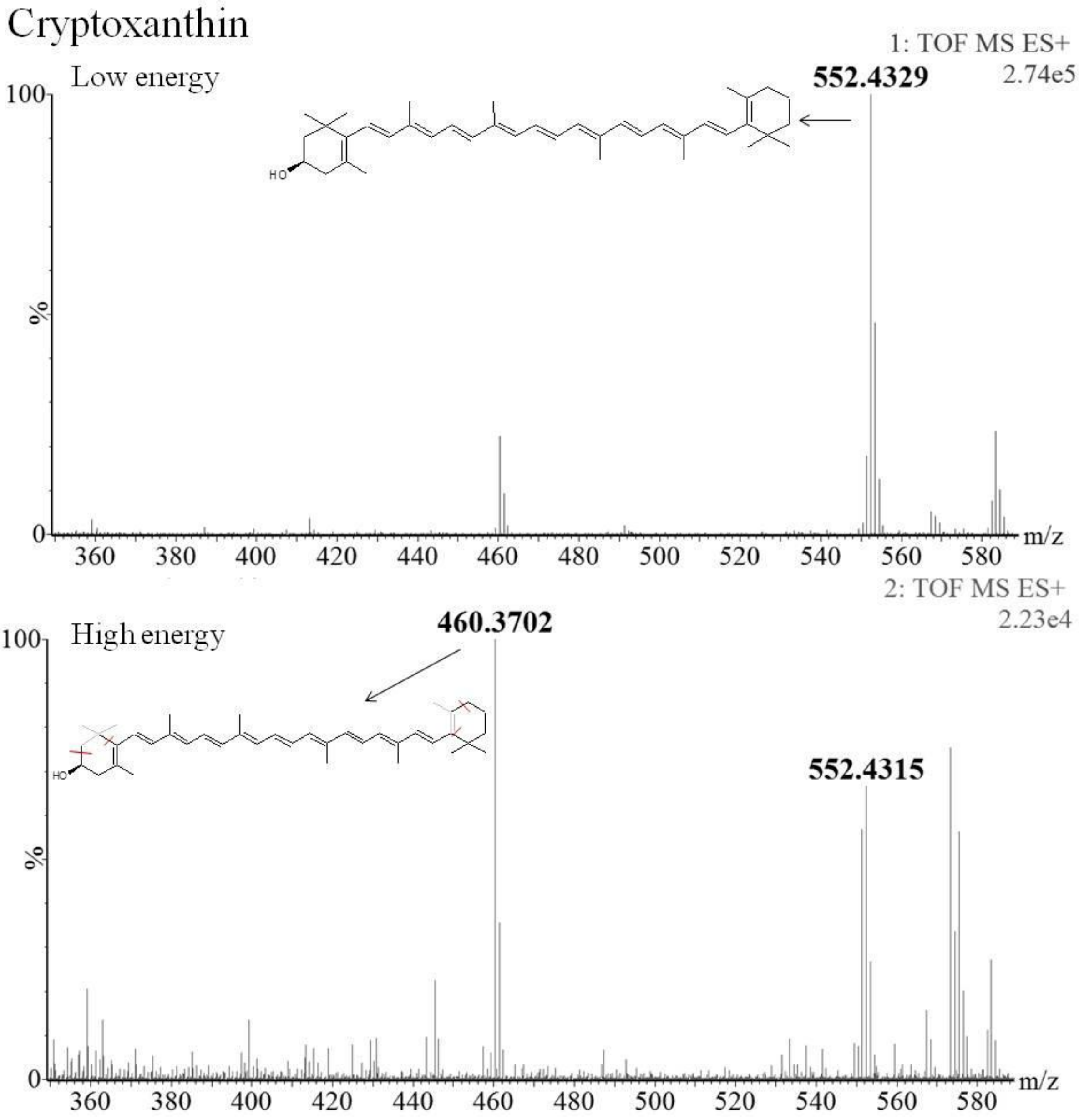


**Figure S1.** *Cont*.

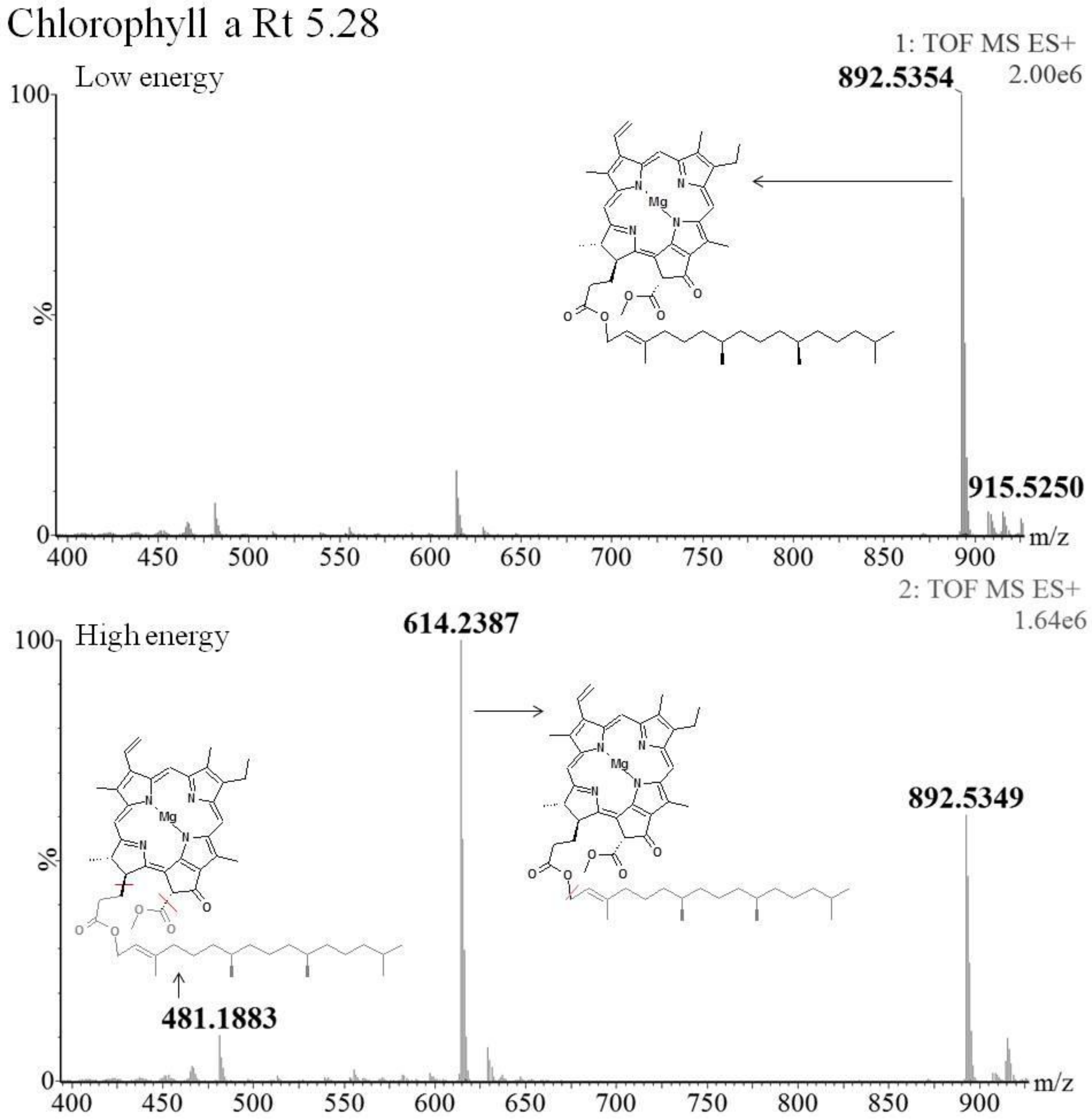


**Figure S1.** *Cont*.

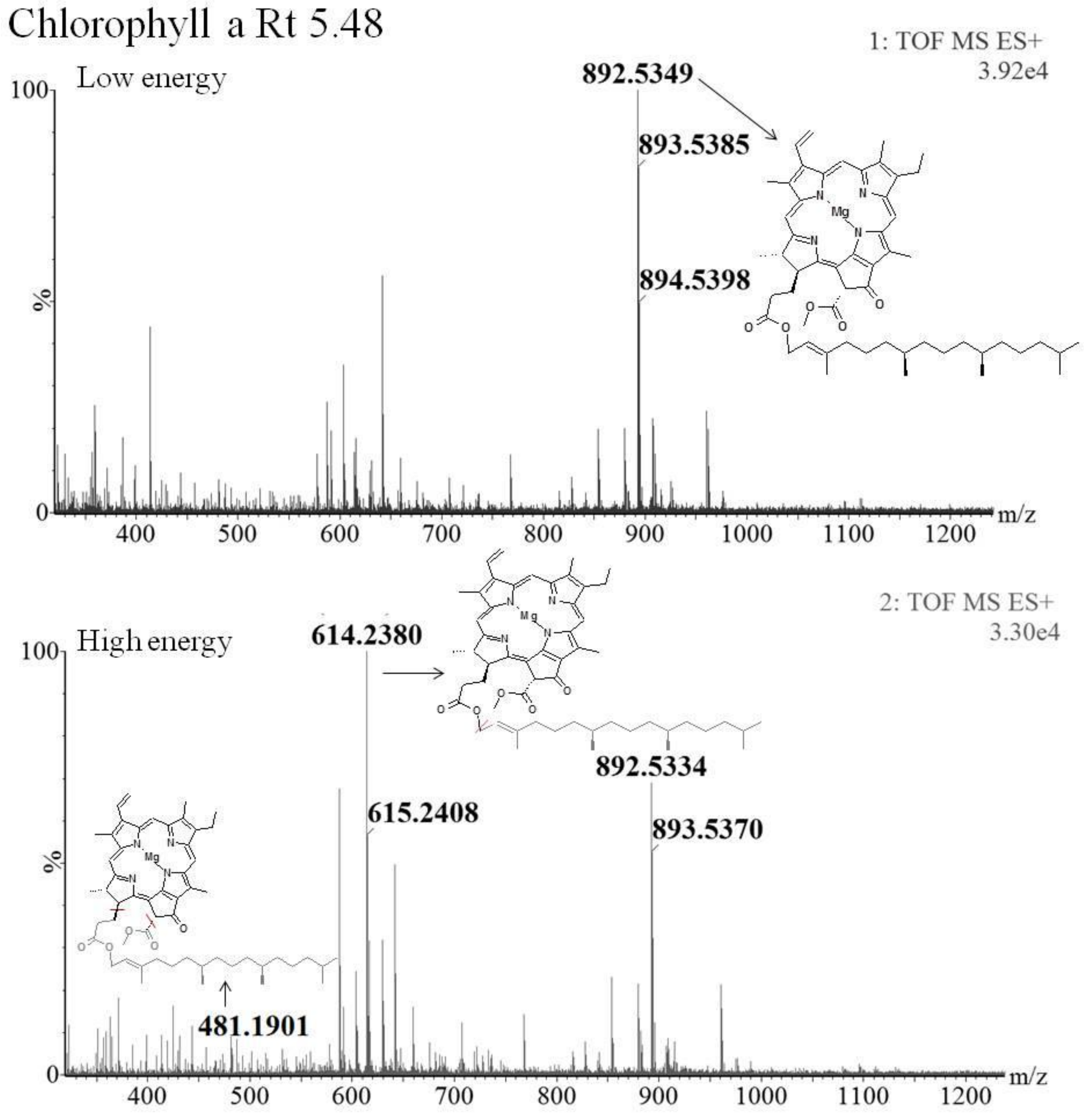


**Figure S1.** *Cont.*

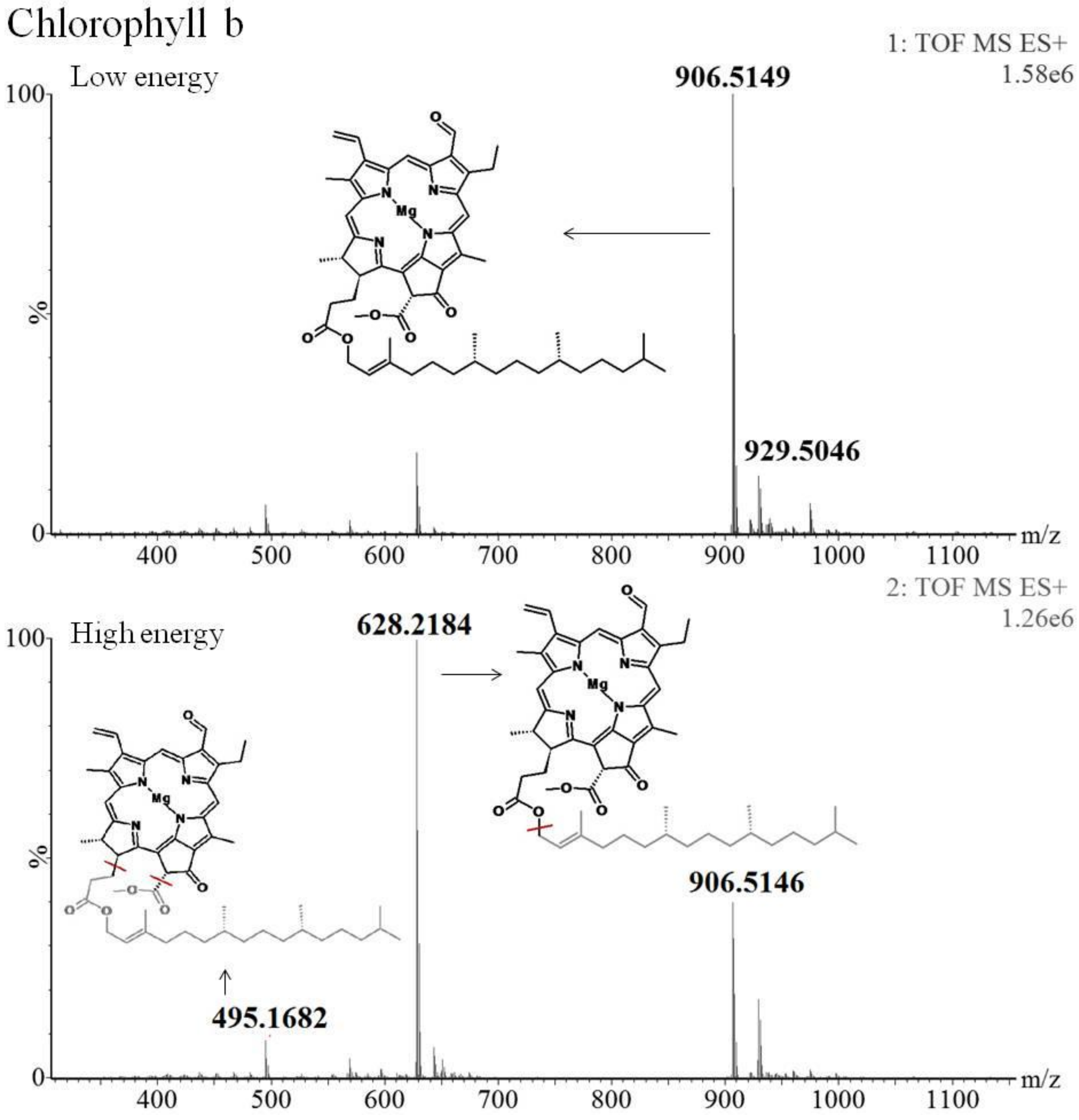


**Figure S1.** *Cont.*

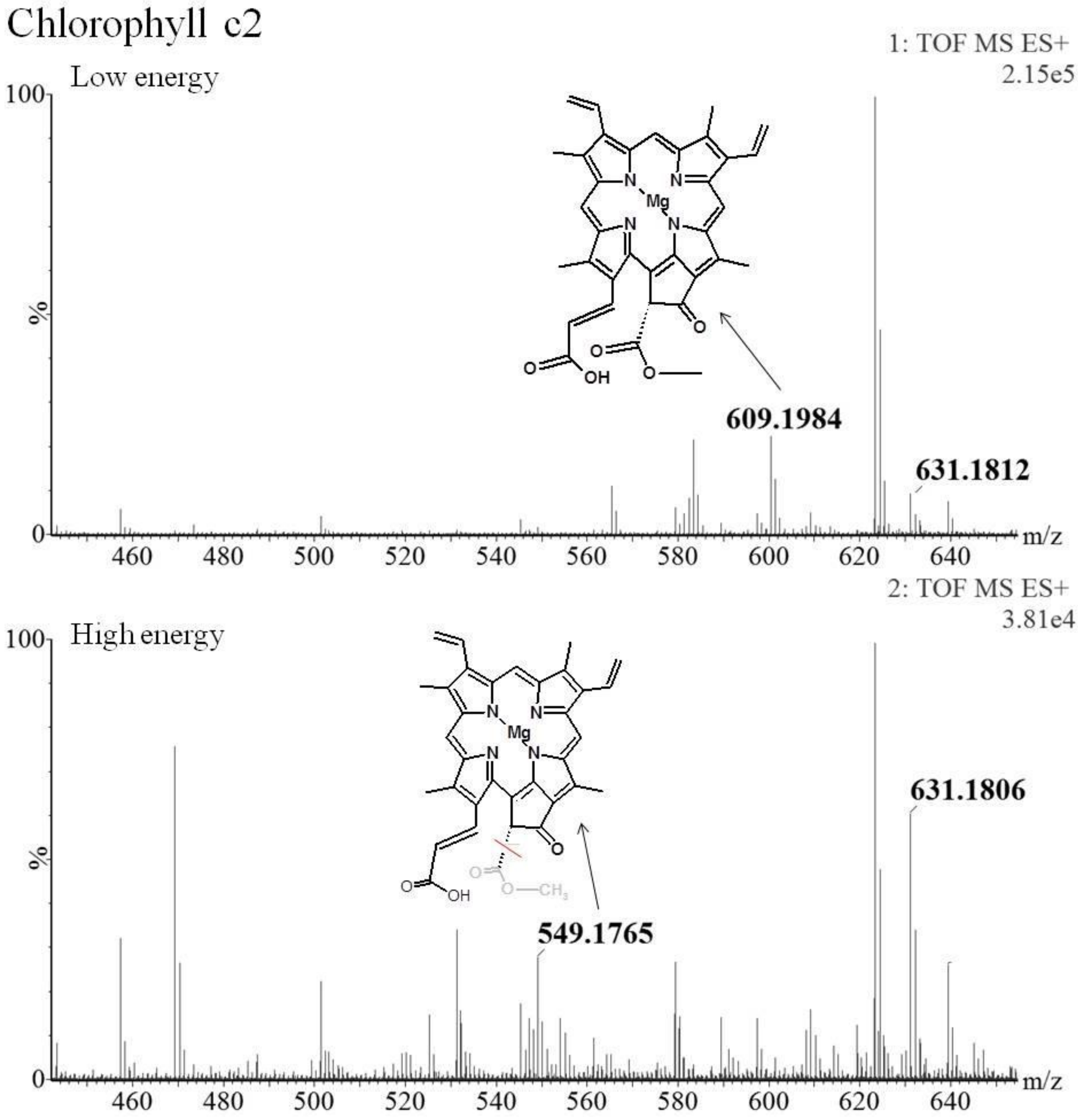


**Figure S1.** *Cont.*

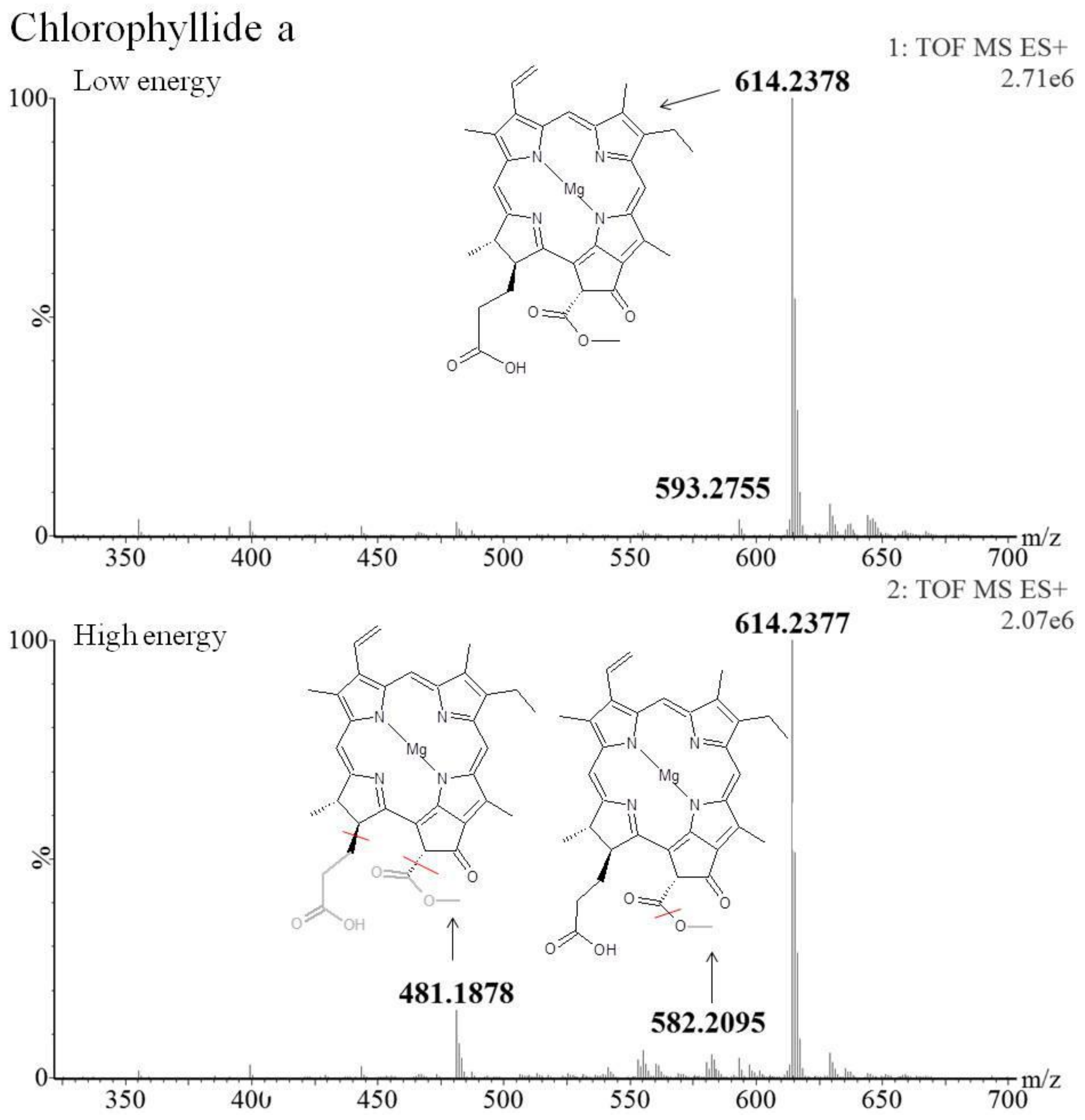


**Figure S1.** *Cont.*

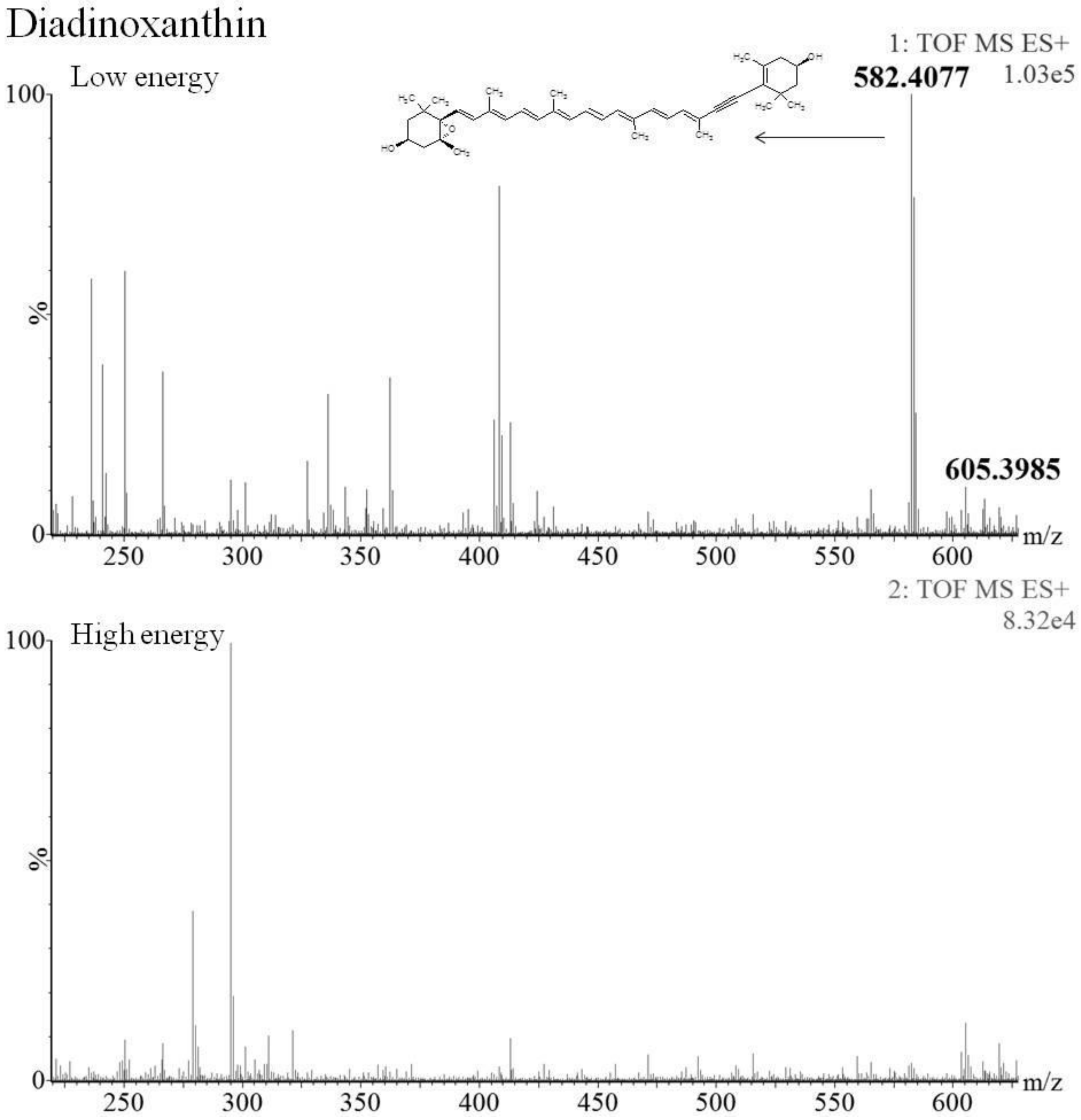


**Figure S1.** *Cont*.

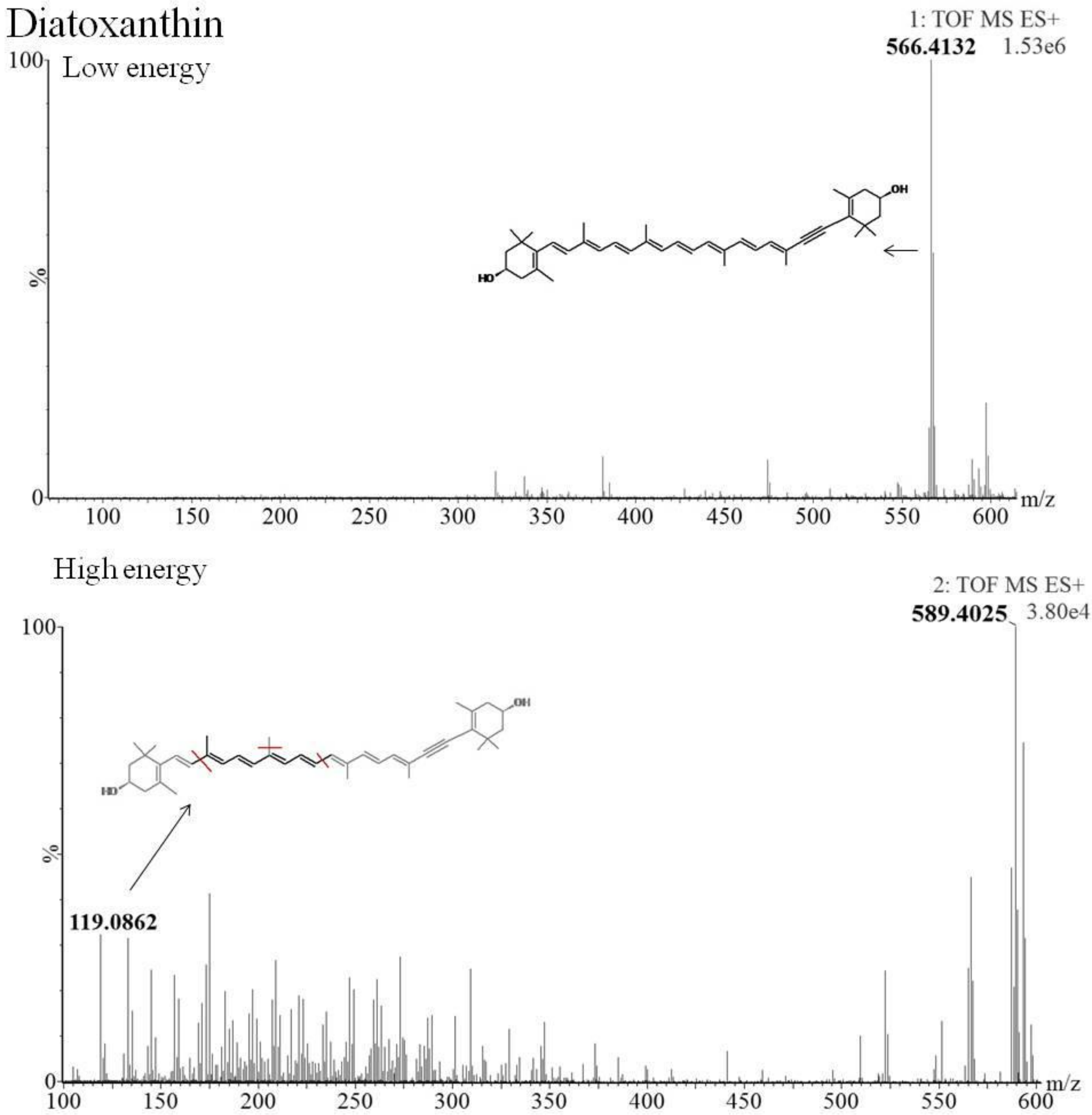


**Figure S1.** *Cont*.

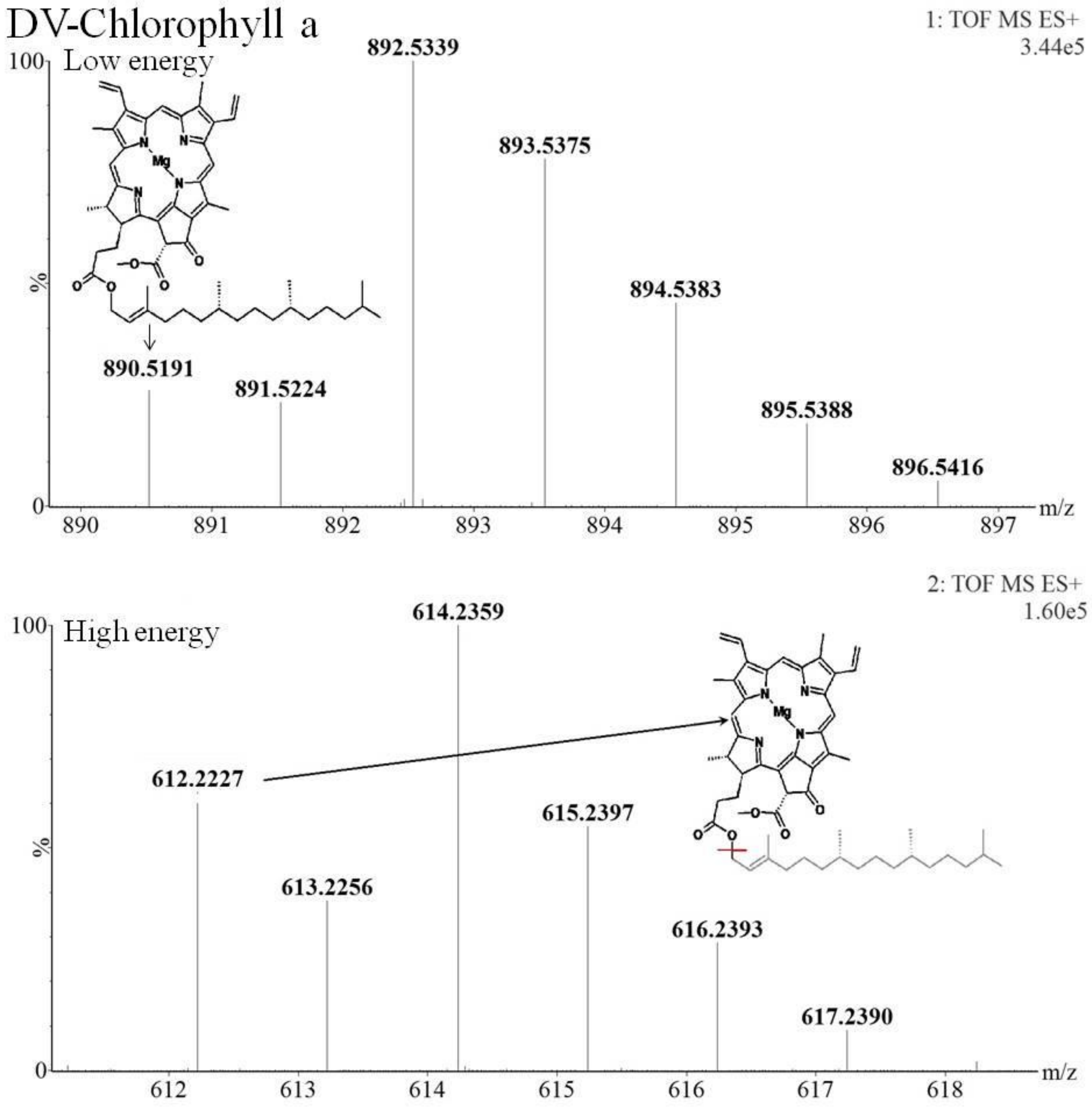


**Figure S1.** *Cont*.

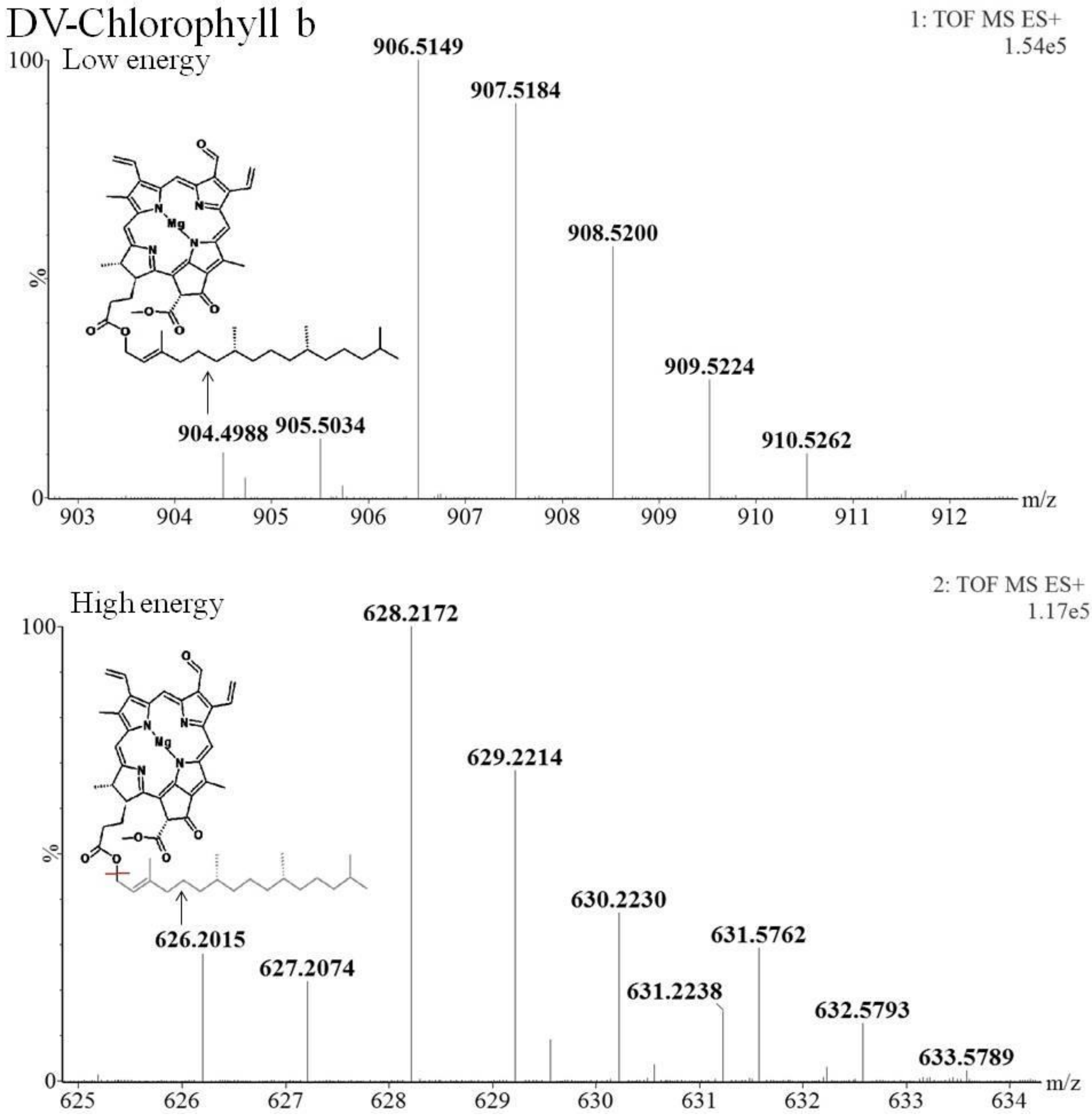


**Figure S1.** *Cont.*

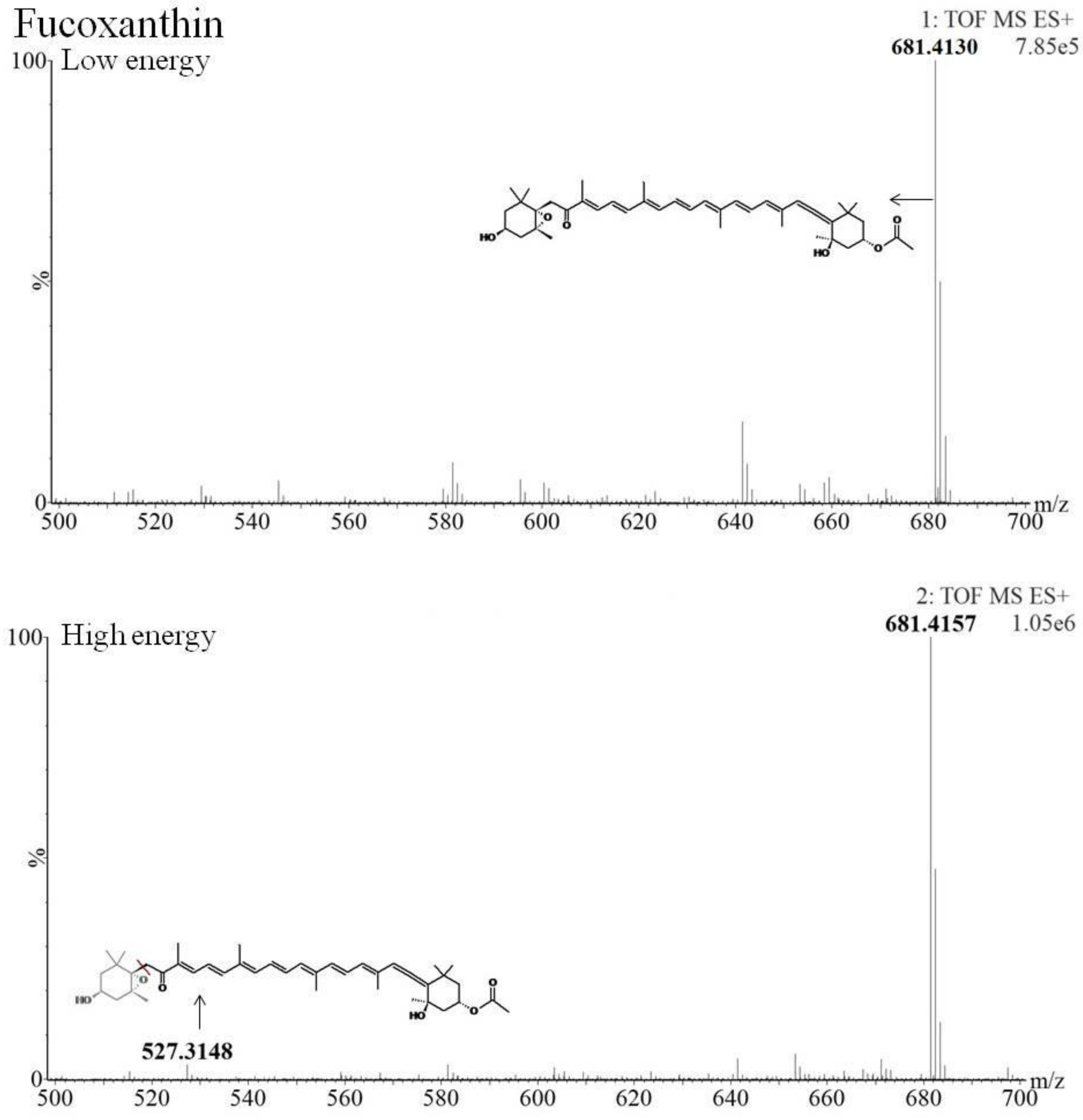


**Figure S1.** *Cont*.

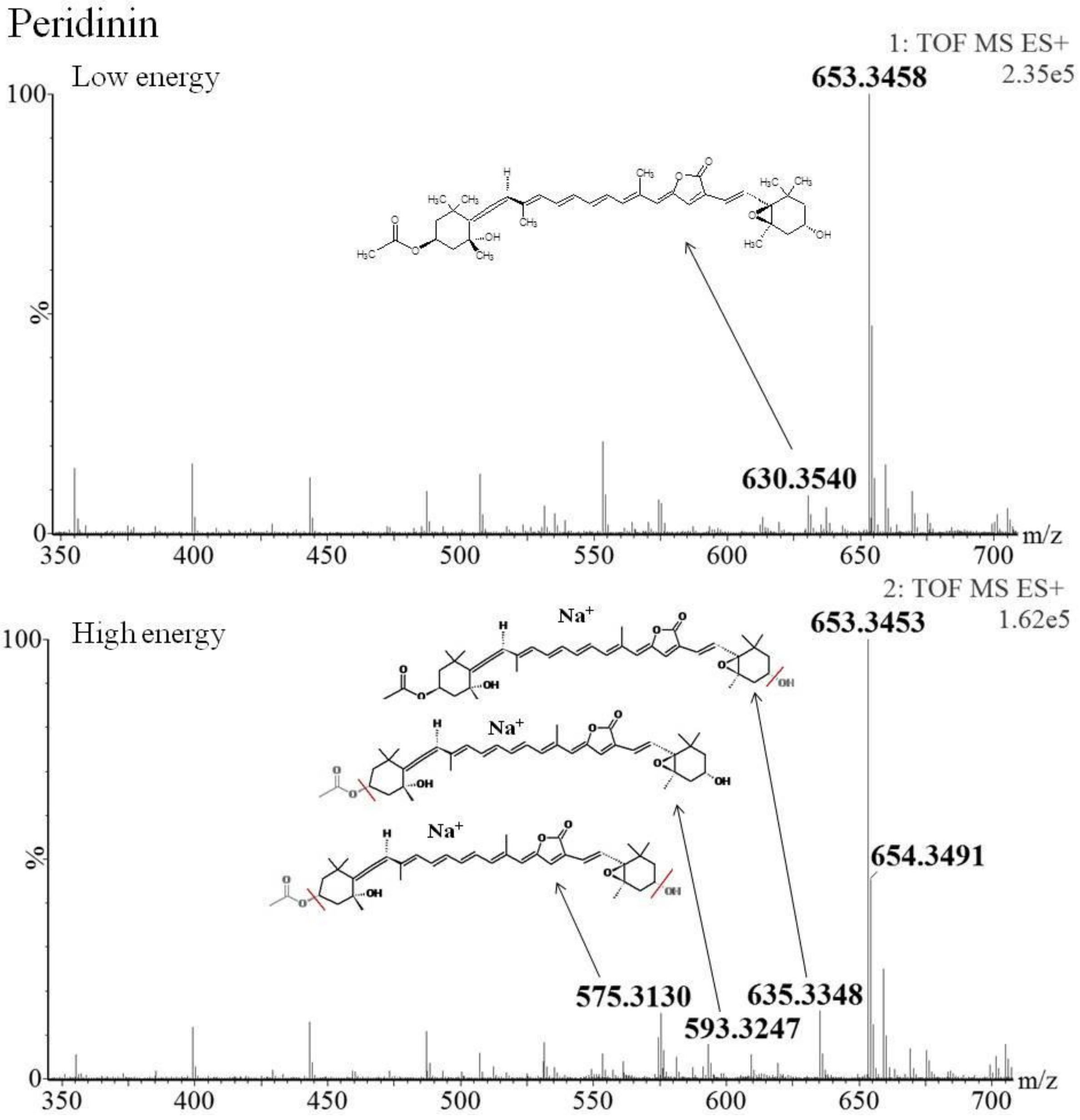


**Figure S1.** *Cont.*

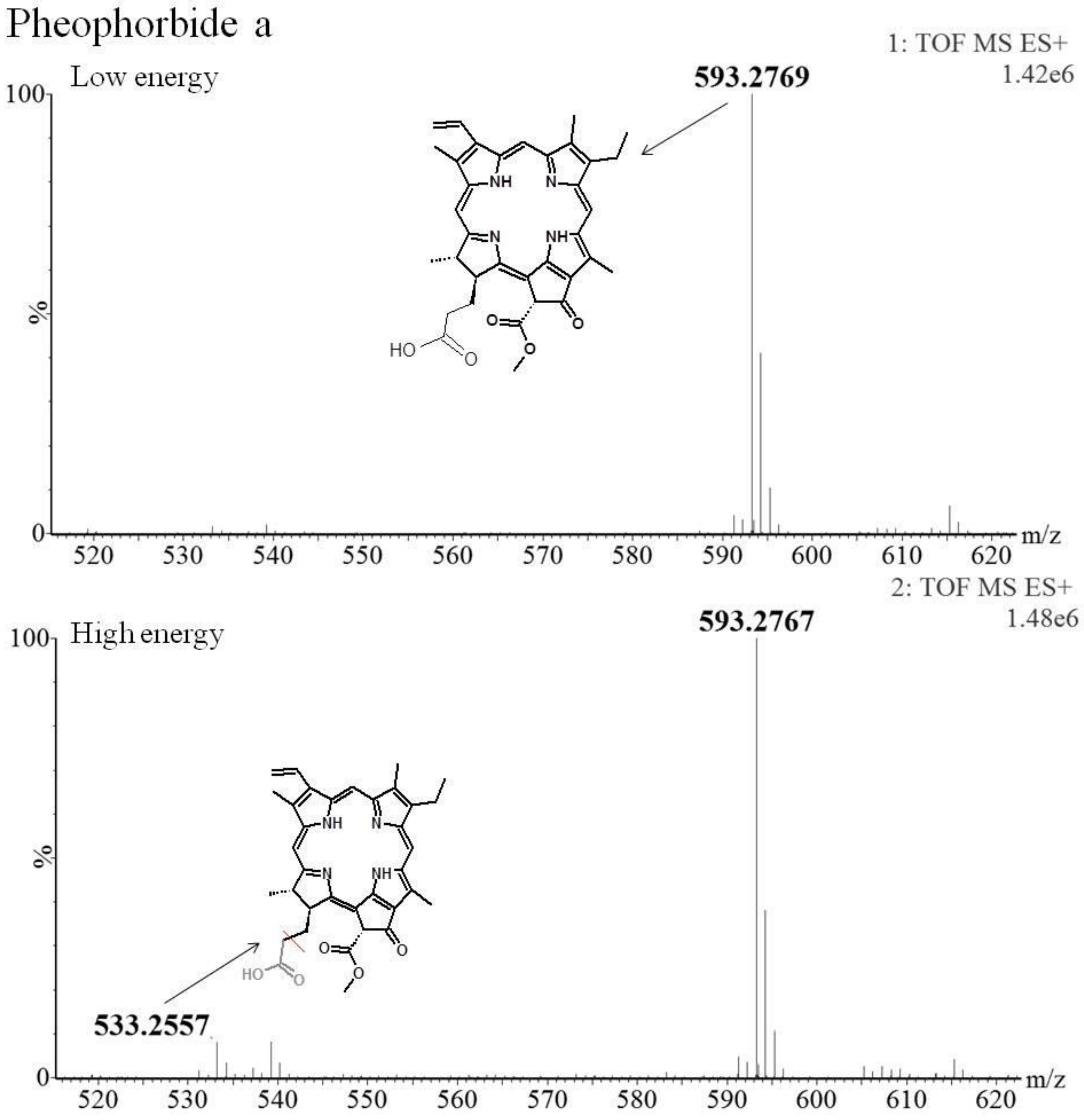


**Figure S1.** *Cont.*

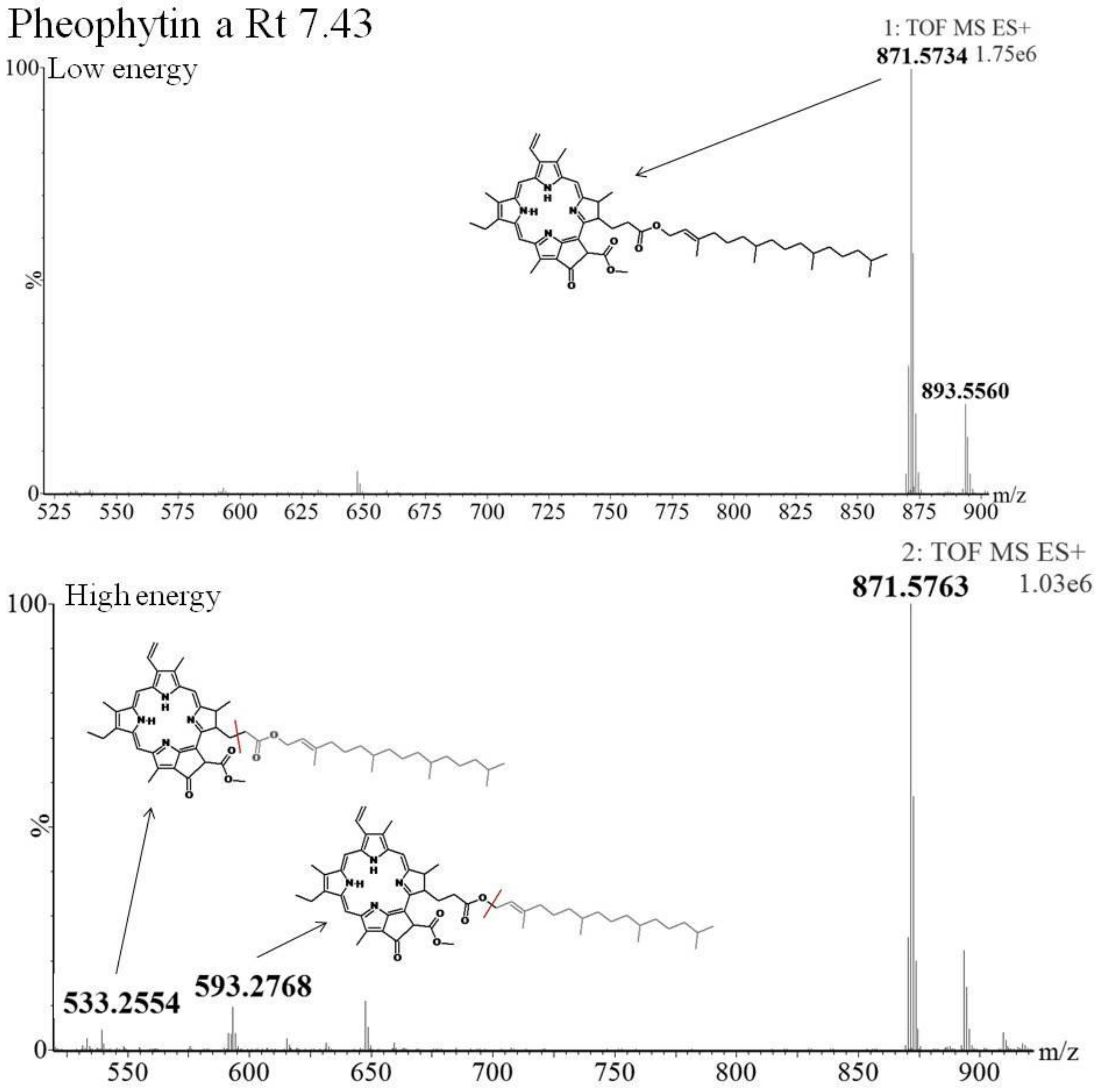


**Figure S1.** *Cont*.

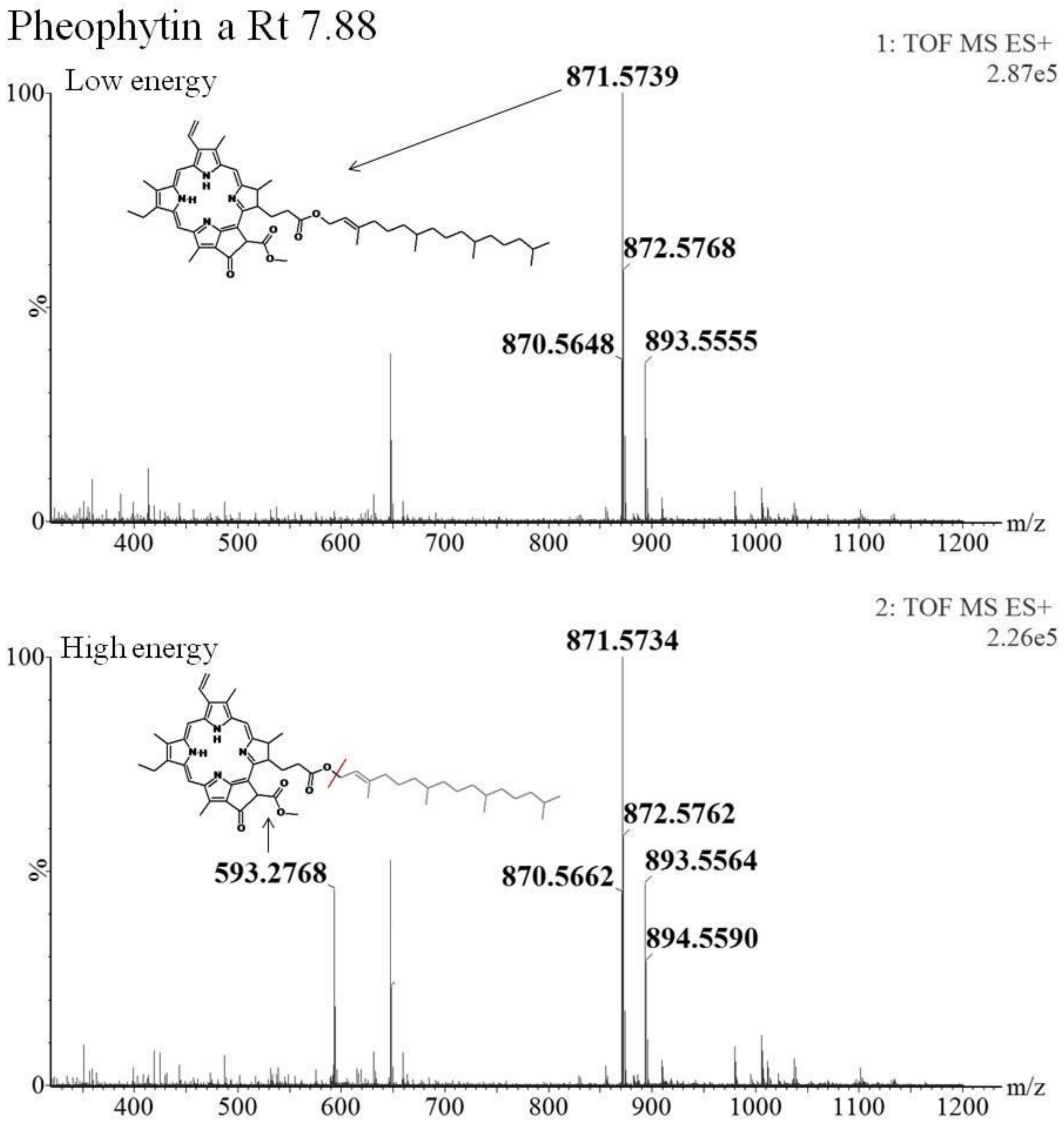


**Figure S1.** *Cont*.

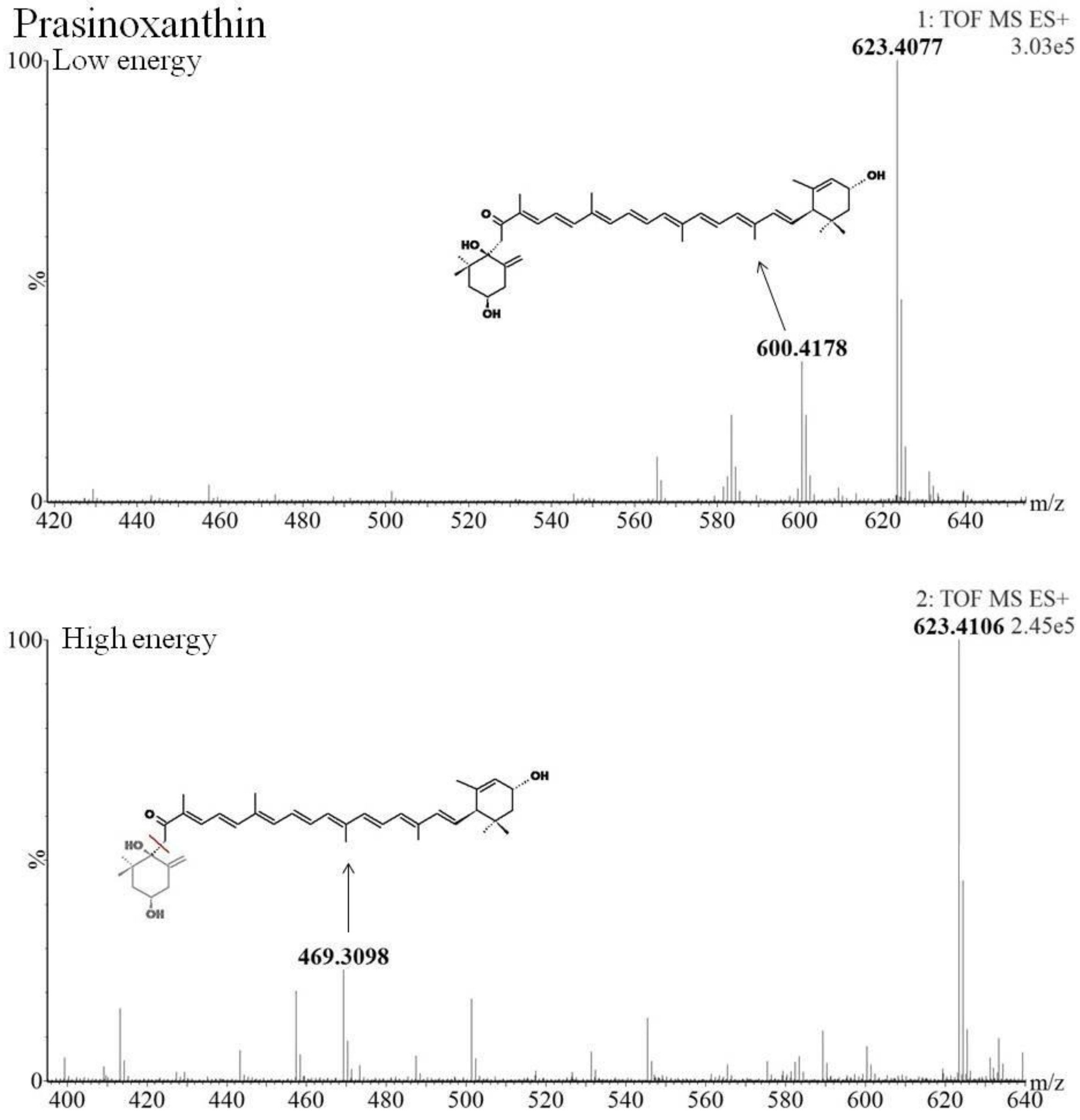


**Figure S1.** *Cont*.

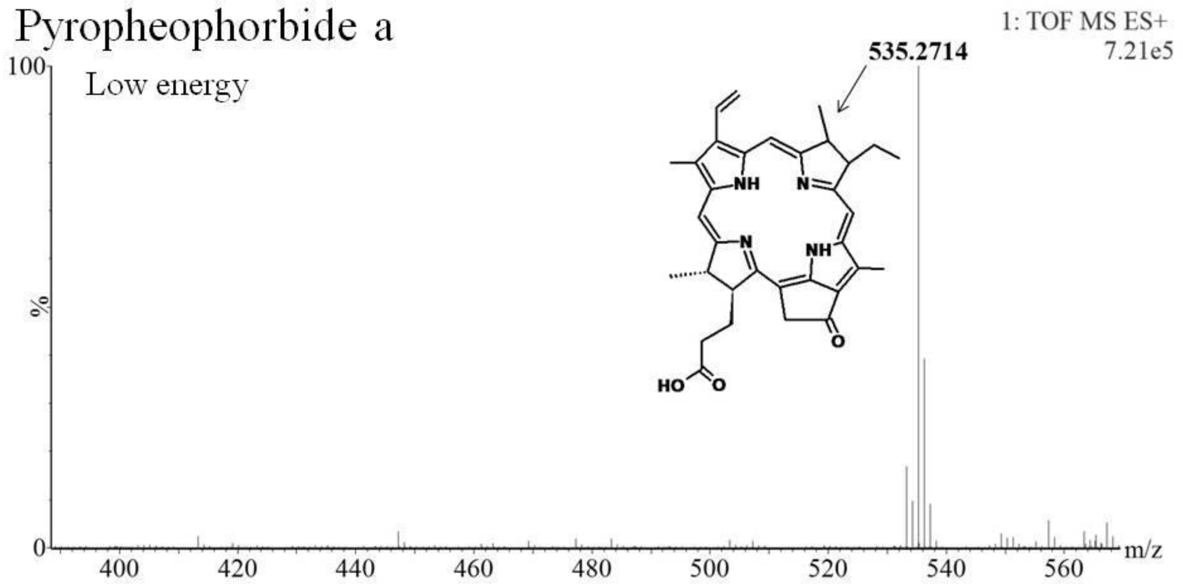


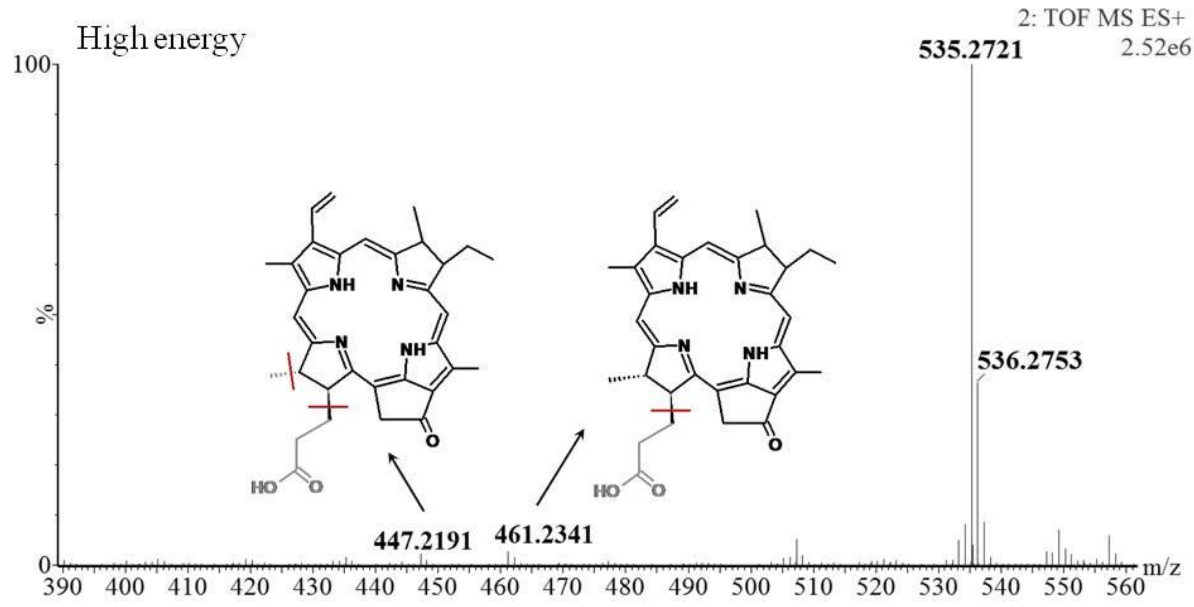


**Figure S1.** *Cont*.

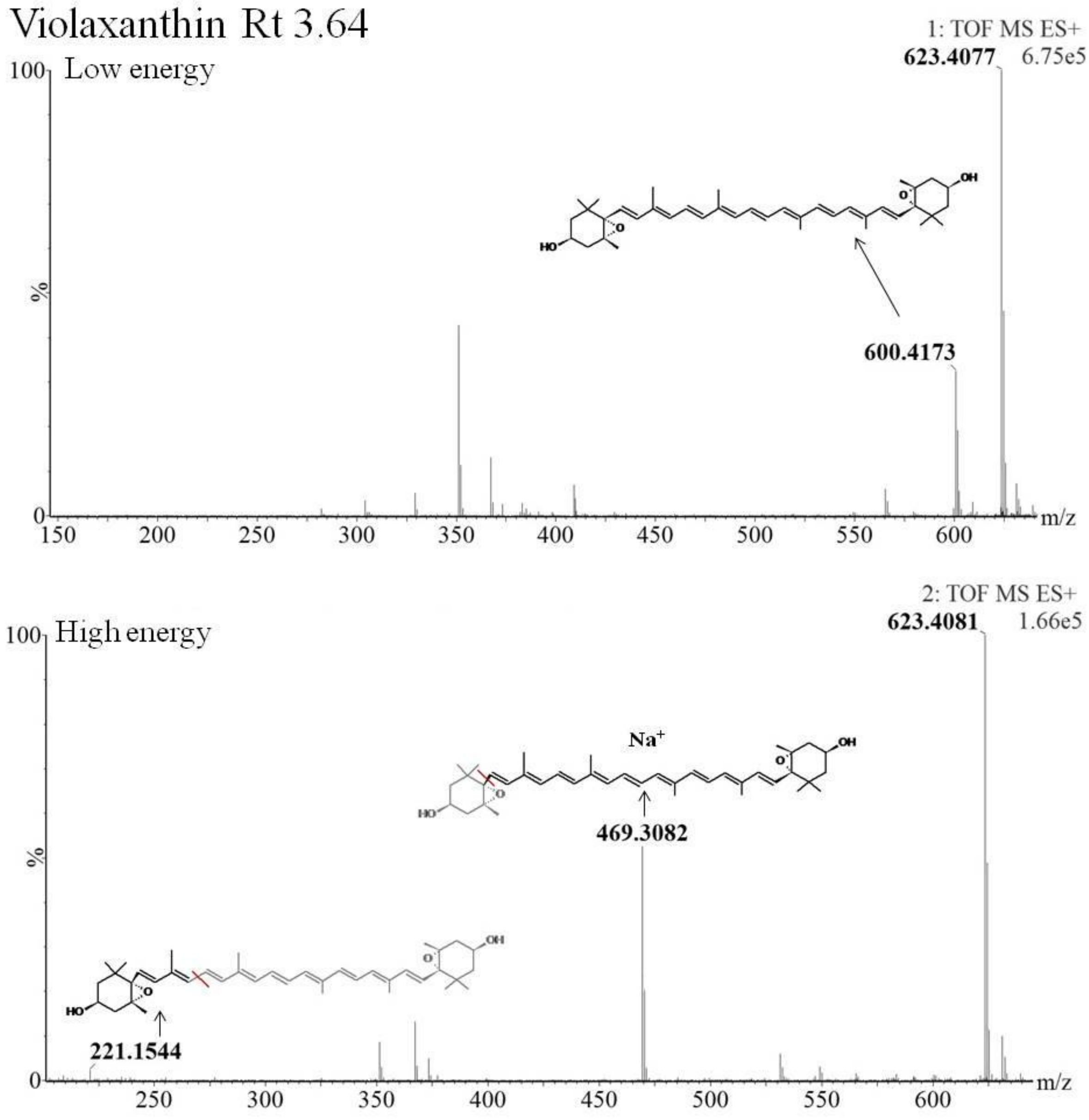


**Figure S1.** *Cont*.

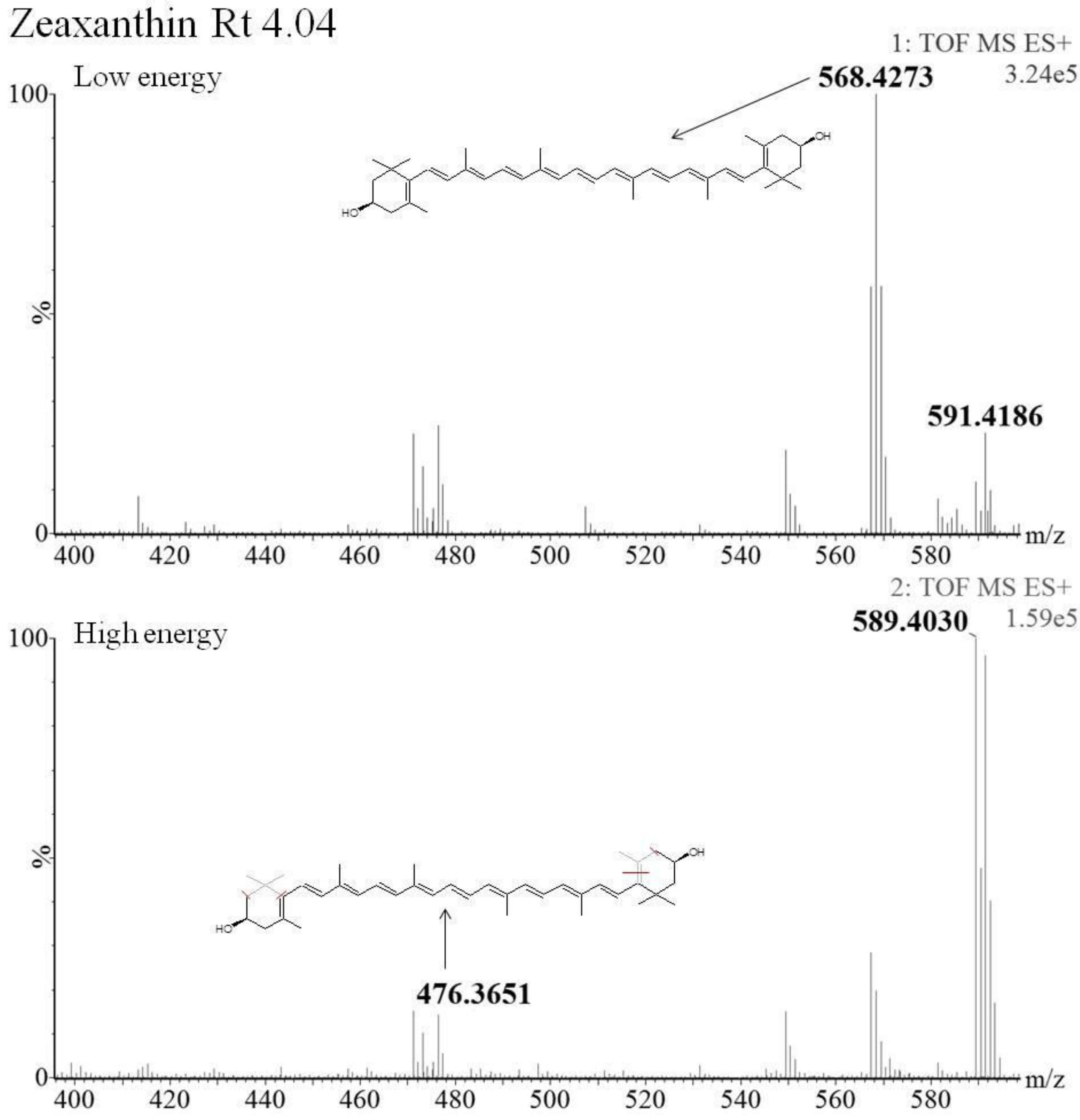


**Figure S1.** *Cont*.

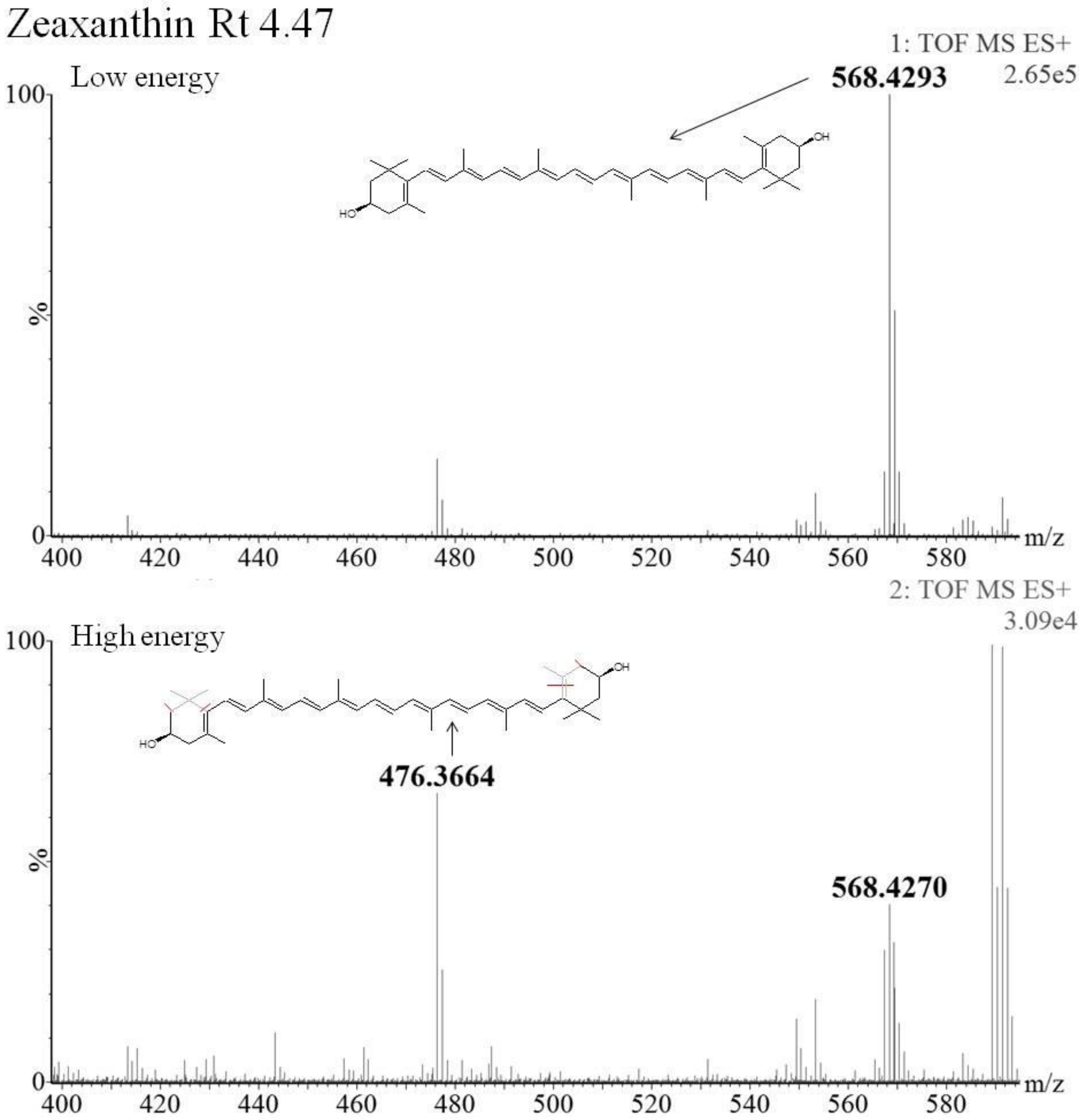


**Figure S1.** High resolution MS$^E$ spectraof standard pigments.

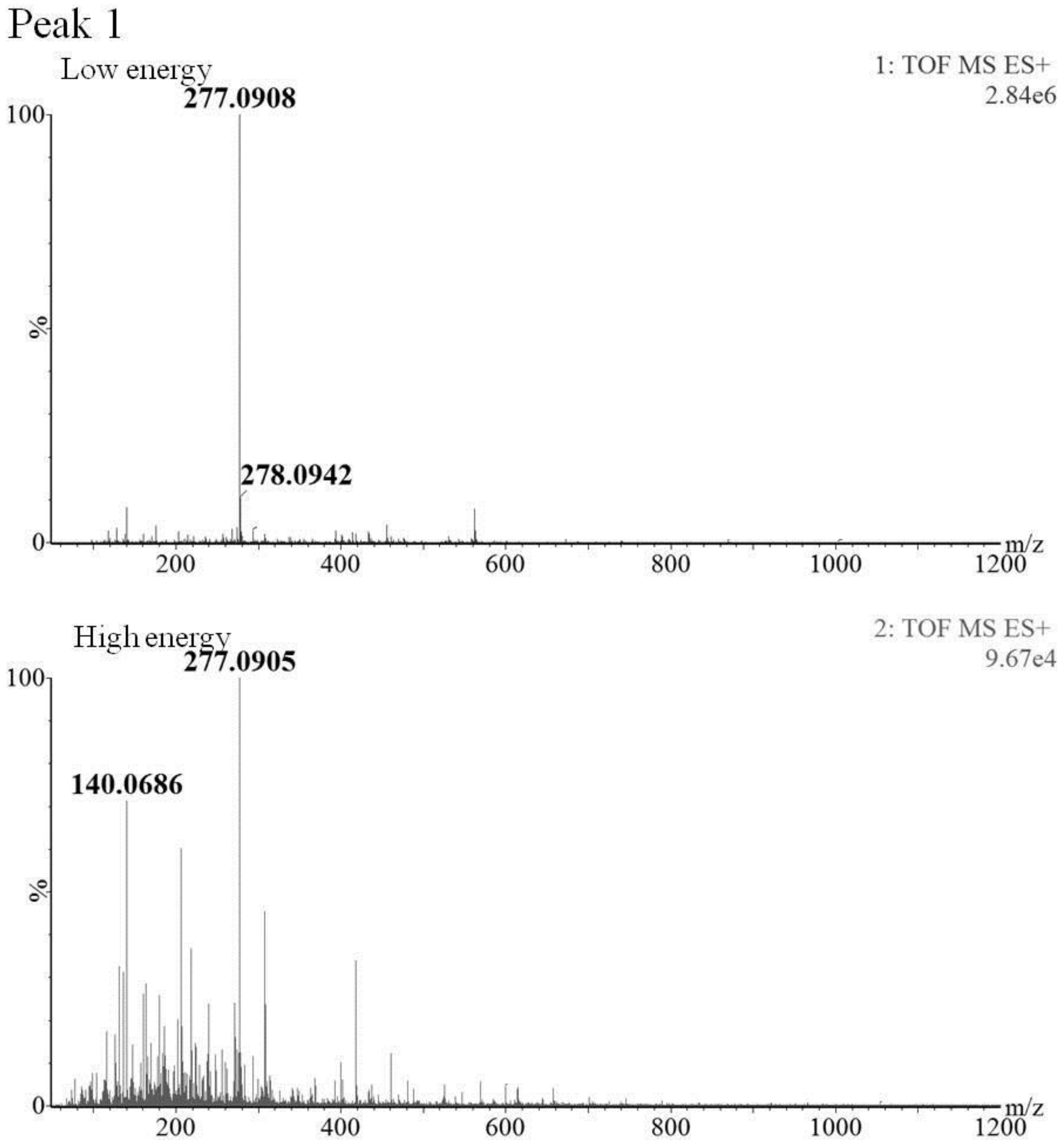


**Figure S2.** *Cont.*

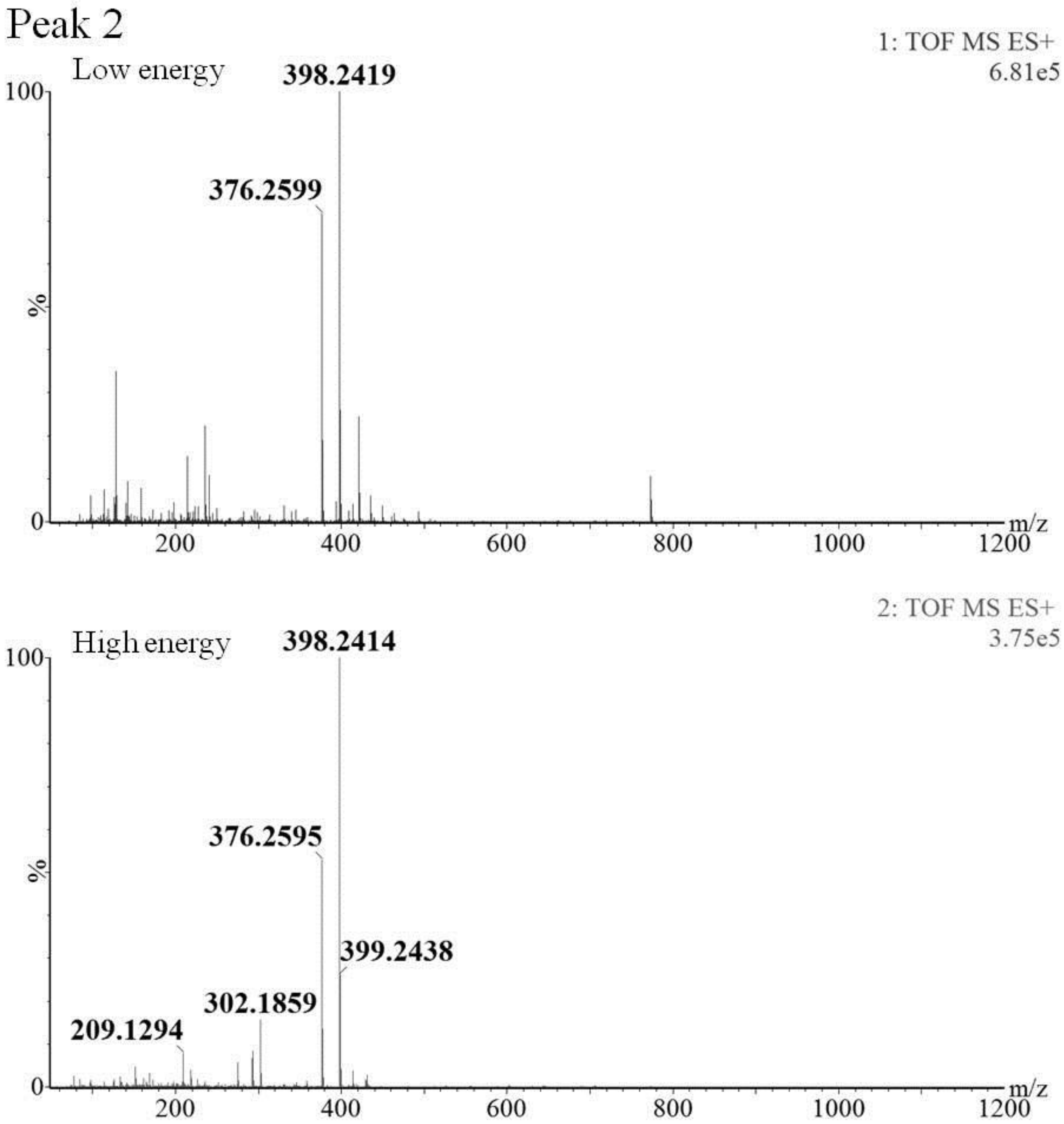


**Figure S2.** *Cont.*

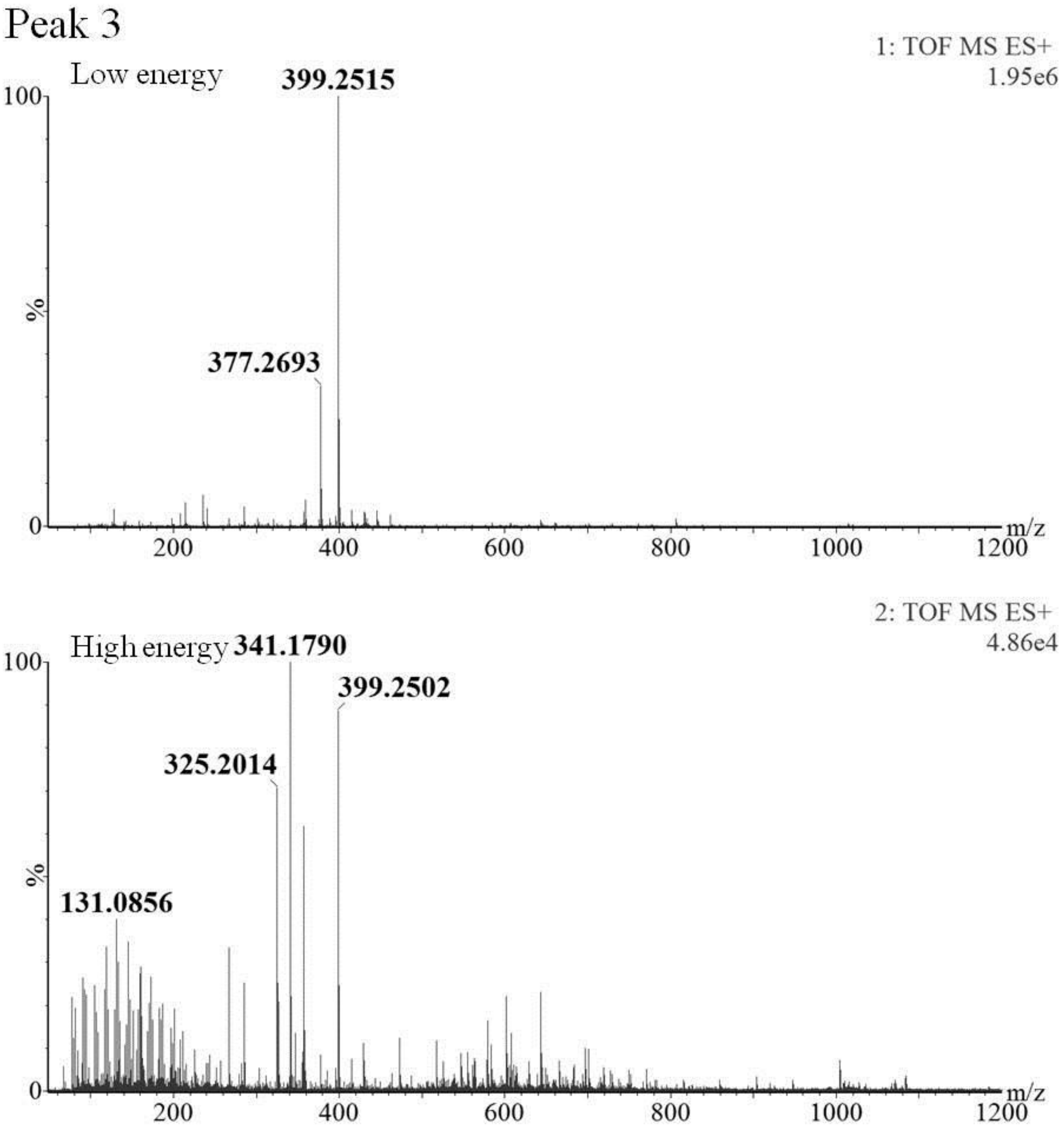


**Figure S2.** *Cont.*

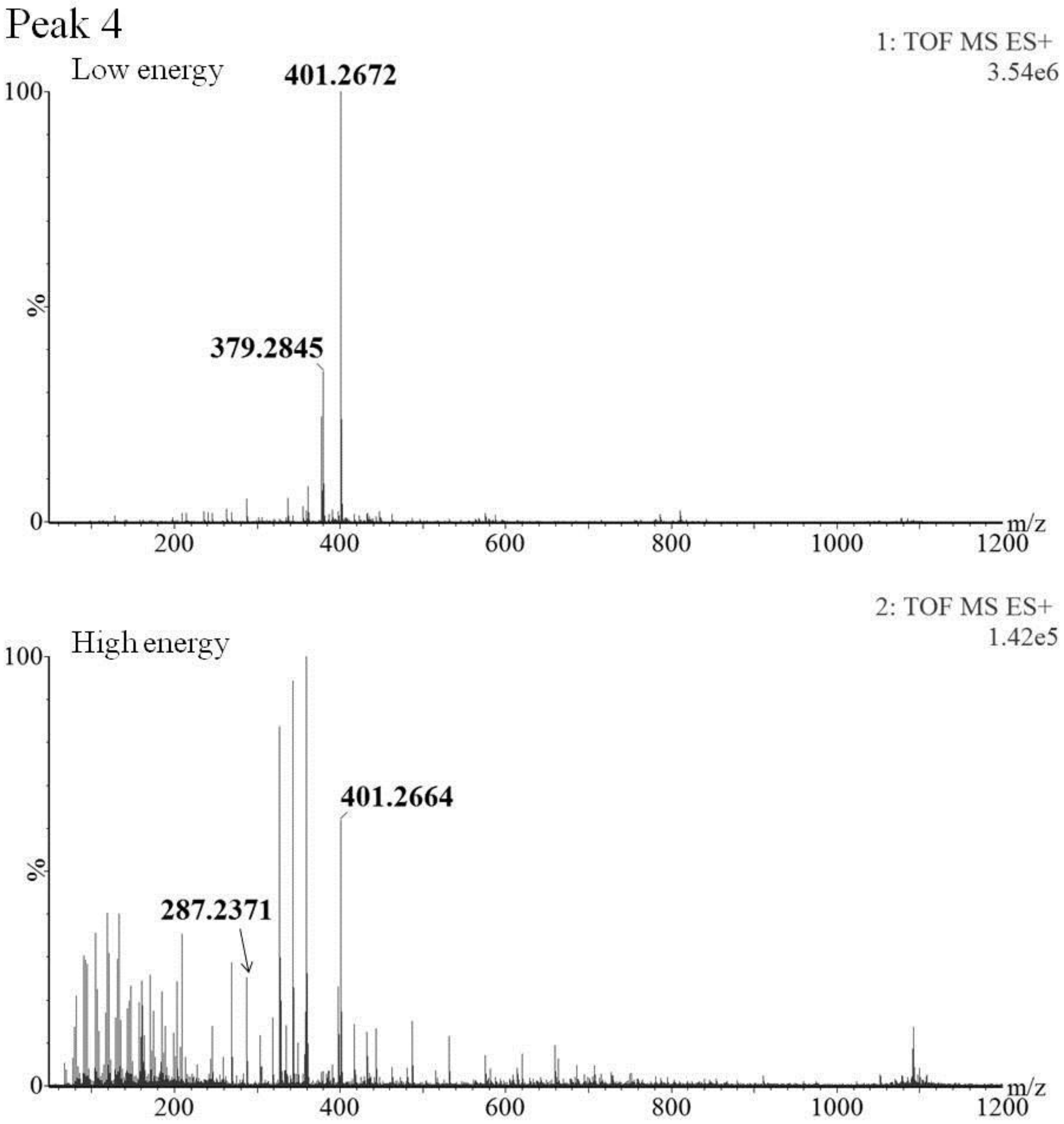


**Figure S2.** *Cont*.

Peak 5

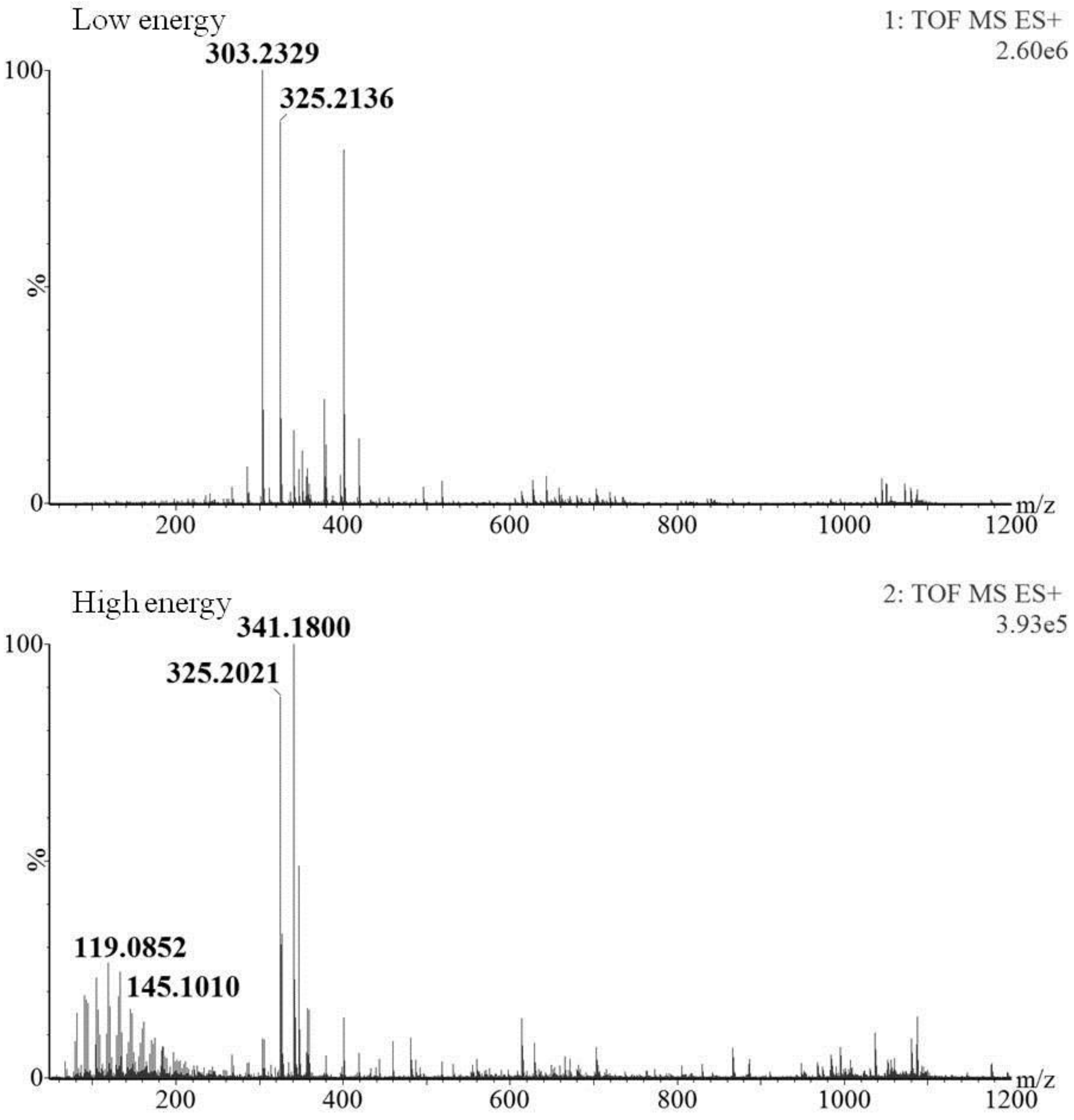


**Figure S2.** *Cont.*

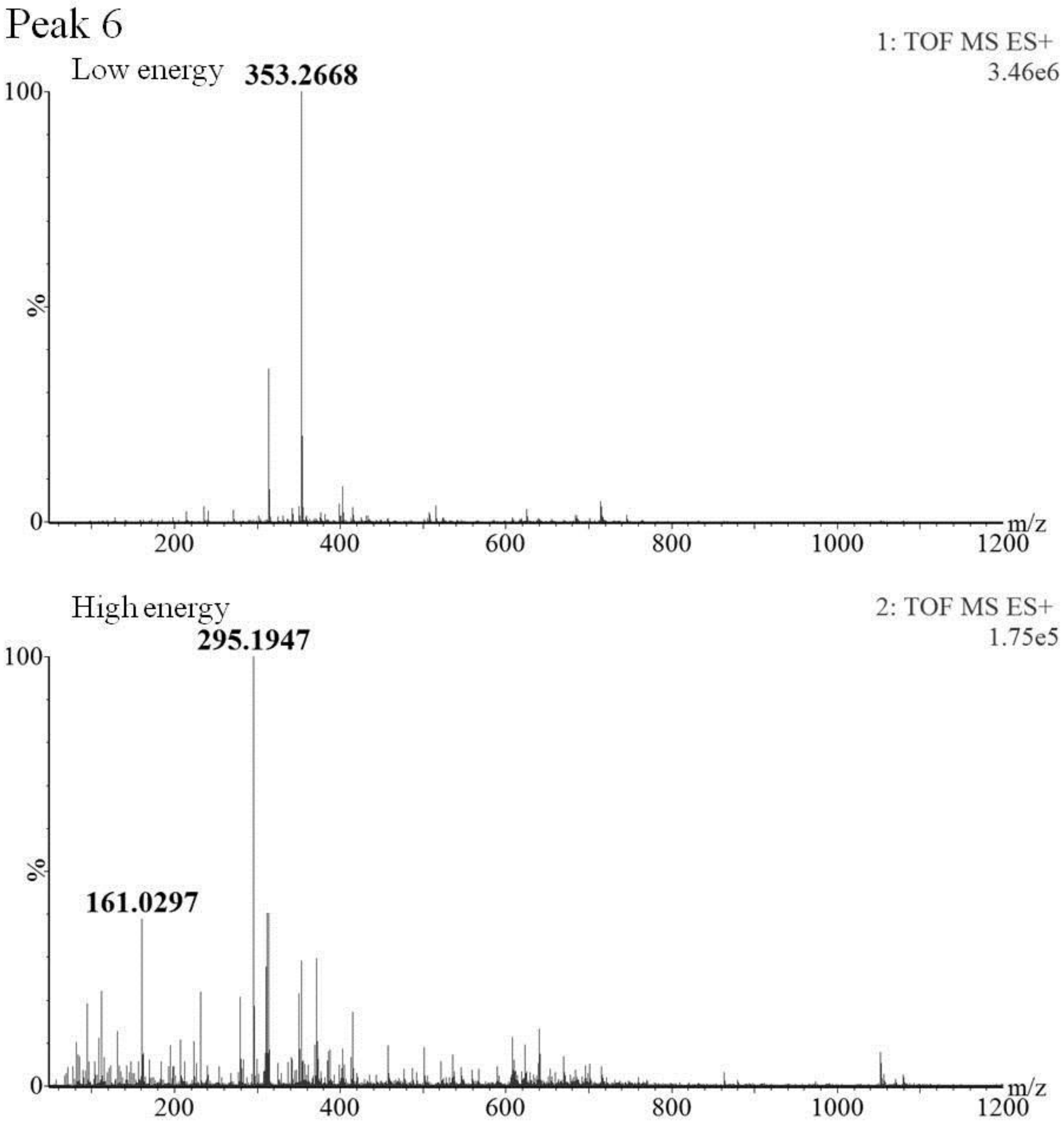


**Figure S2.** *Cont*.

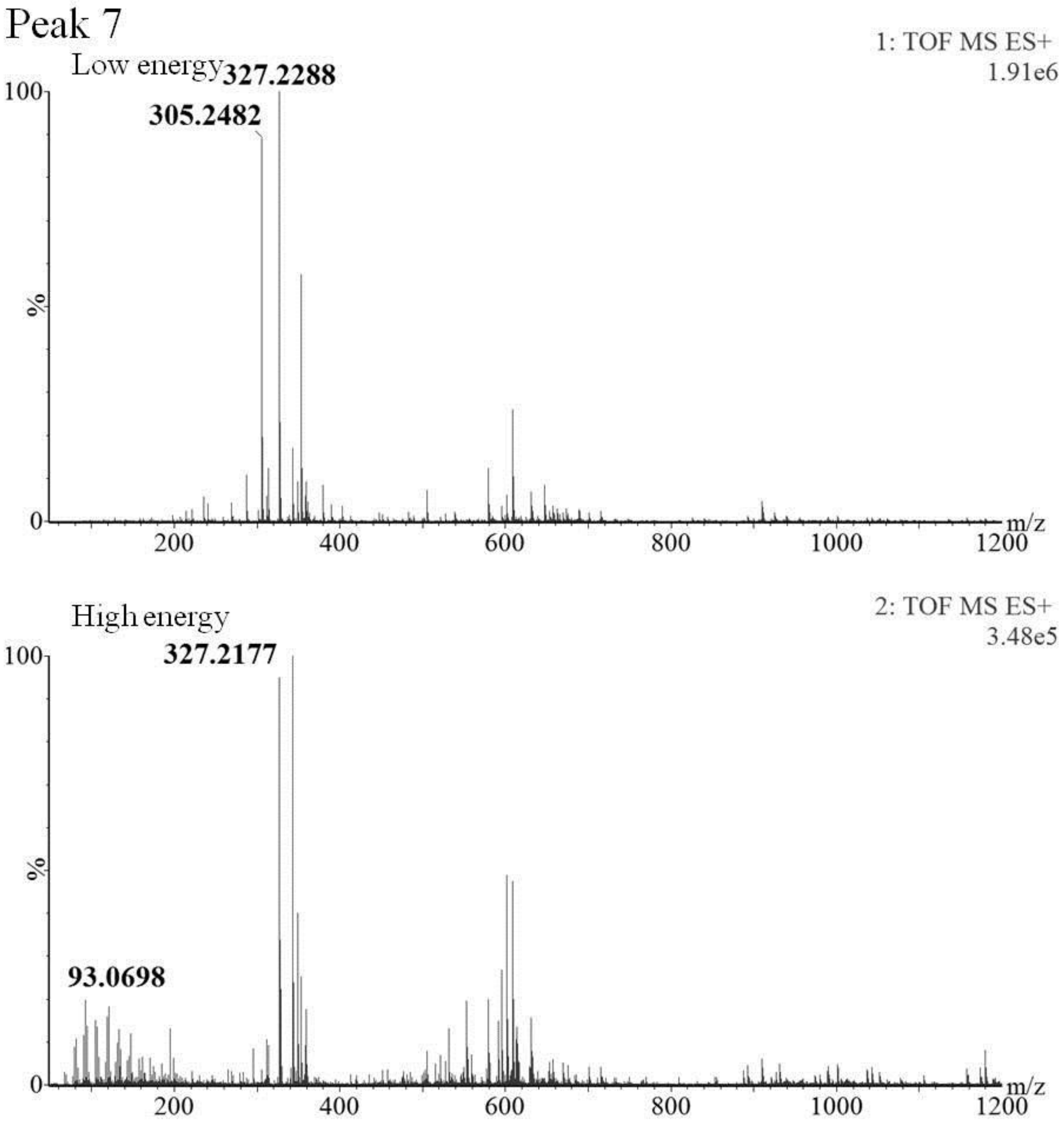


**Figure S2.** *Cont*.

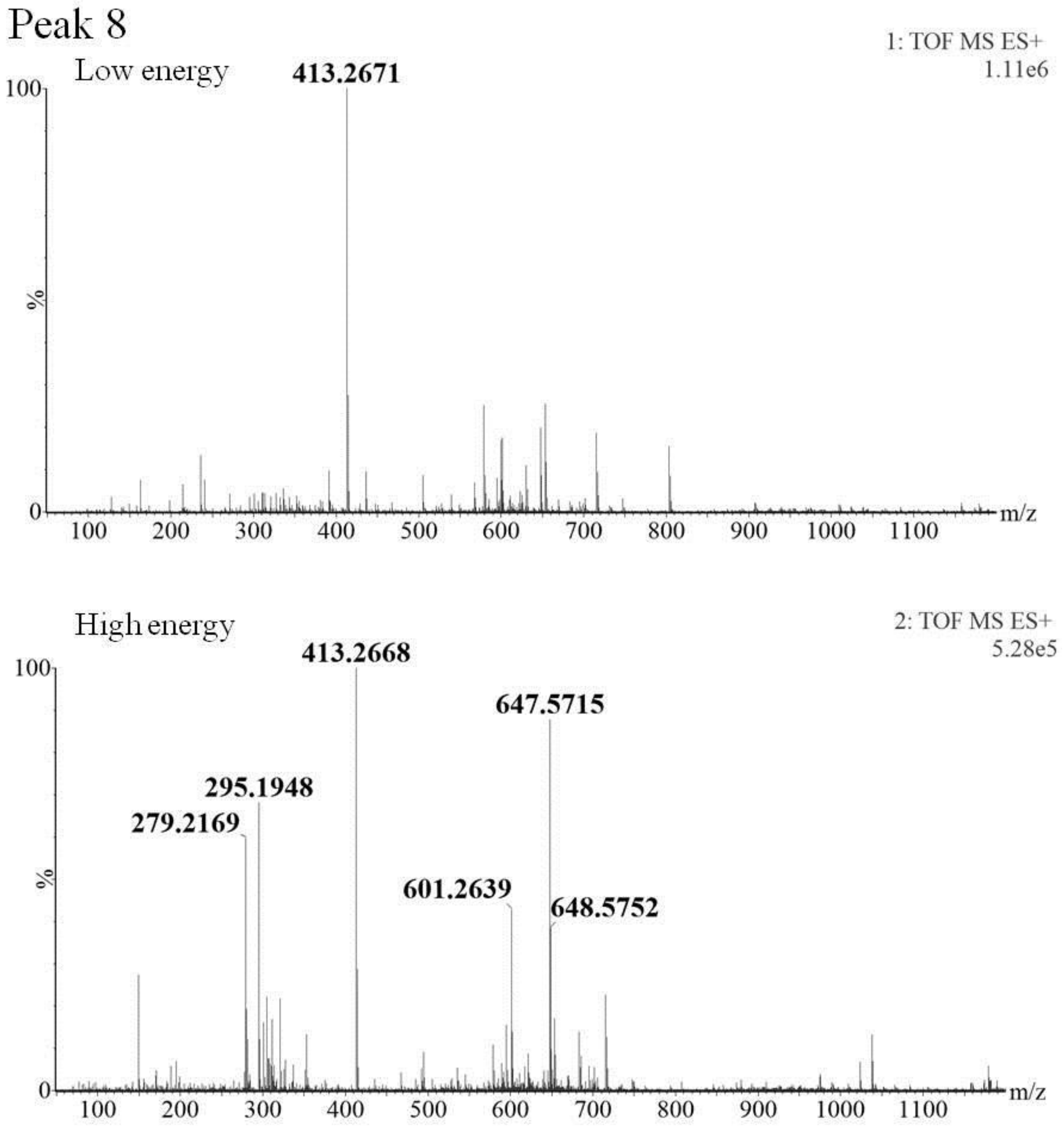


**Figure S2.** *Cont.*

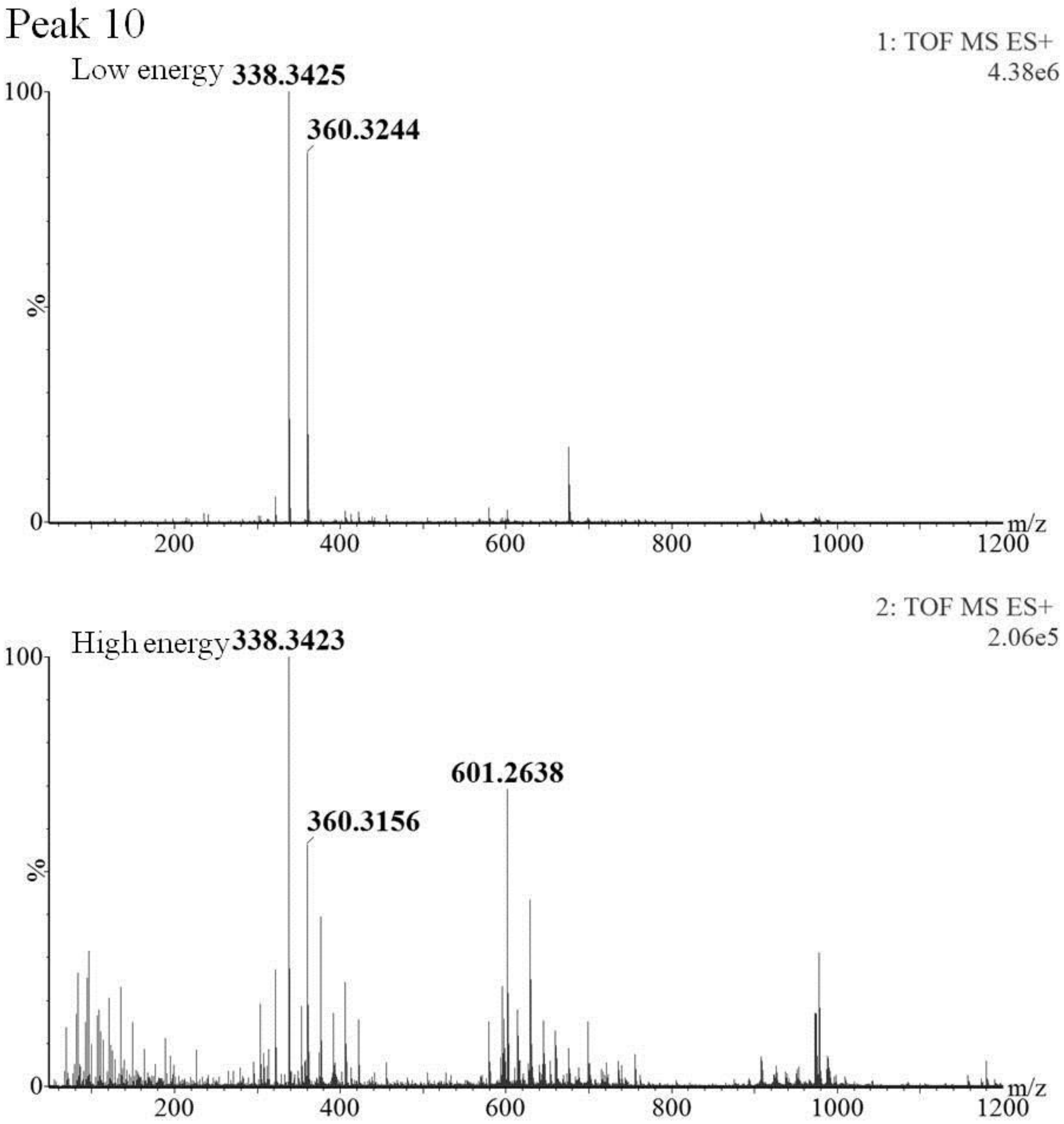


**Figure S2.** *Cont.*

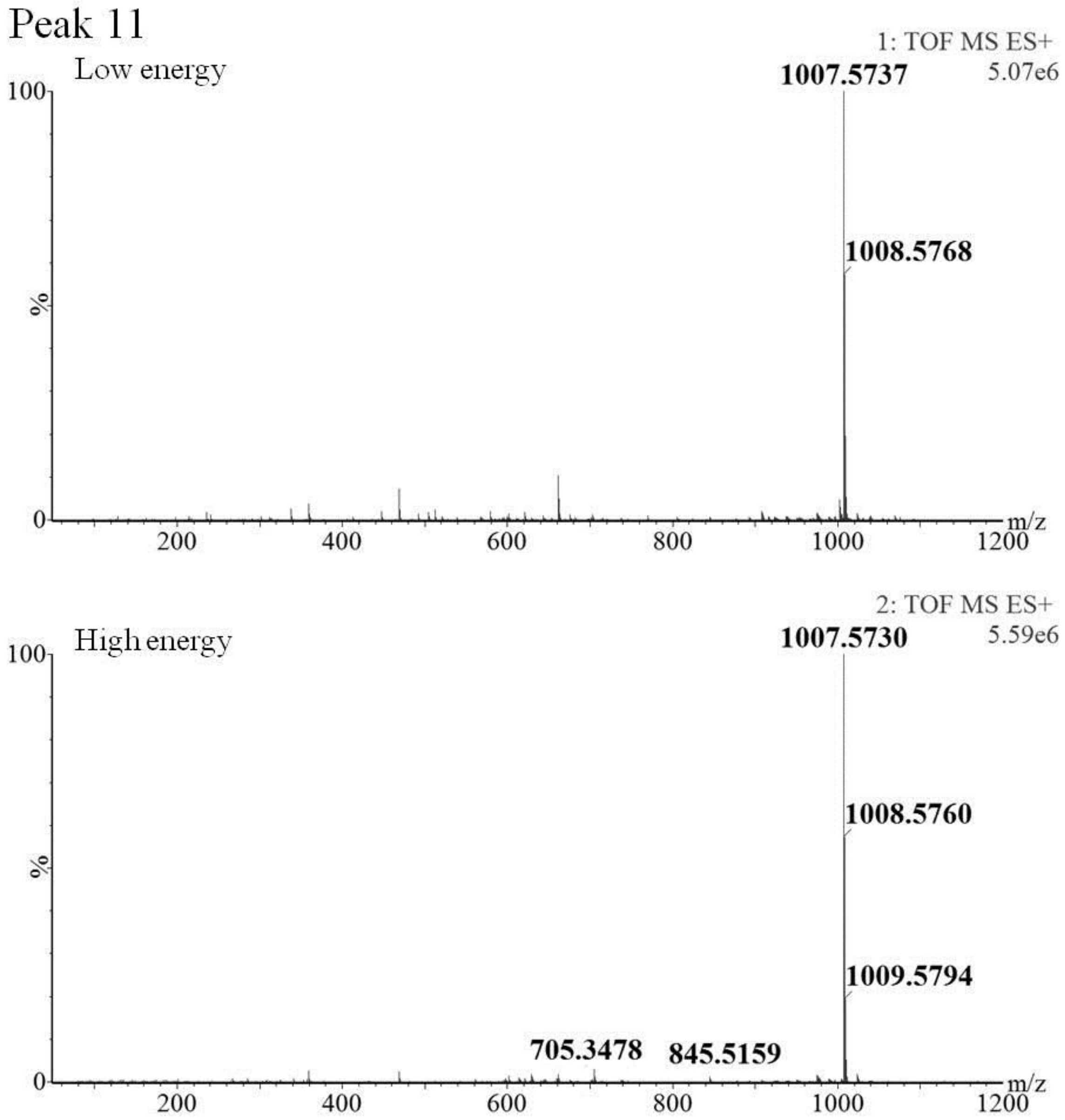


**Figure S2.** *Cont*.

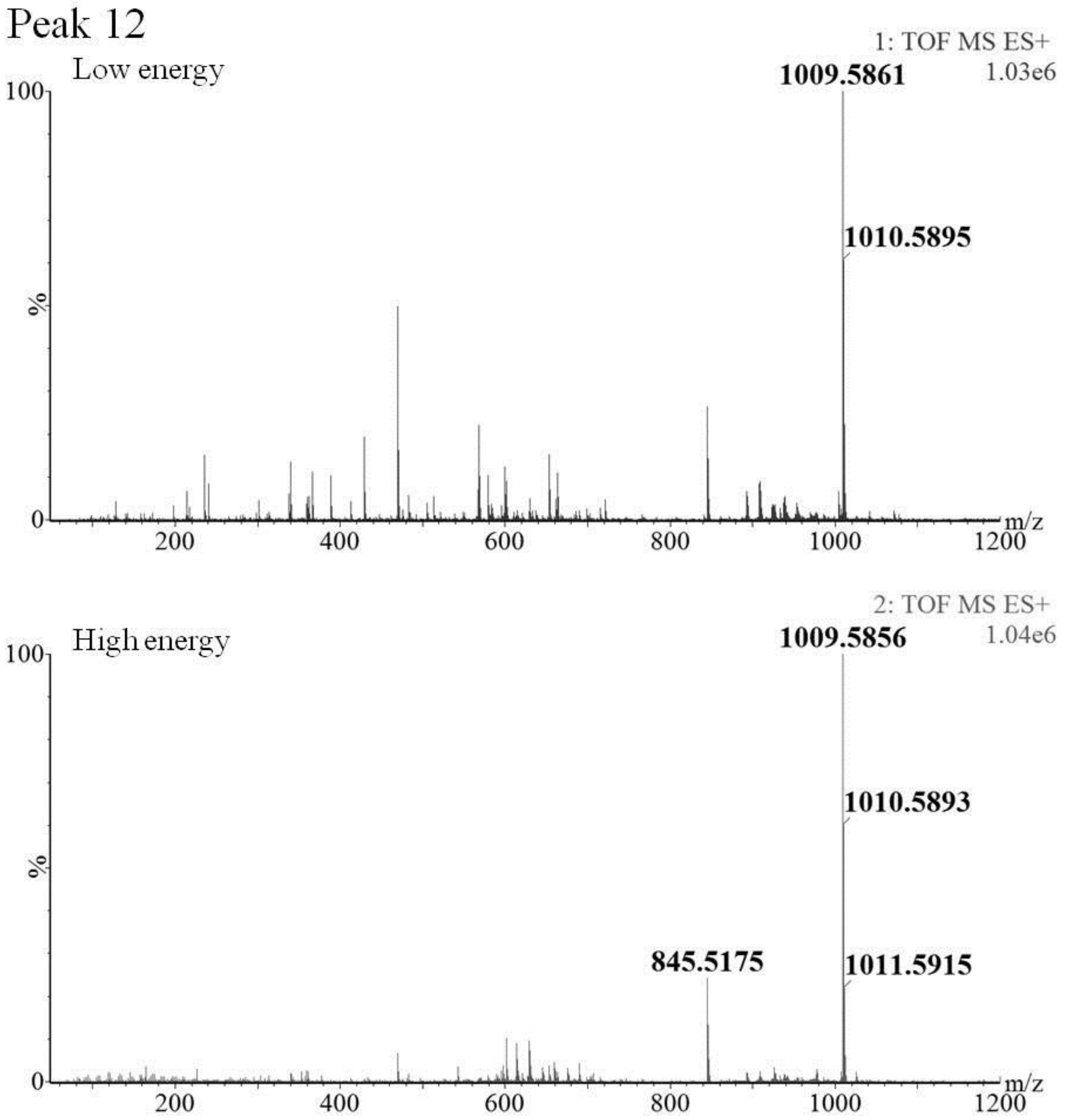


**Figure S2.** *Cont*.

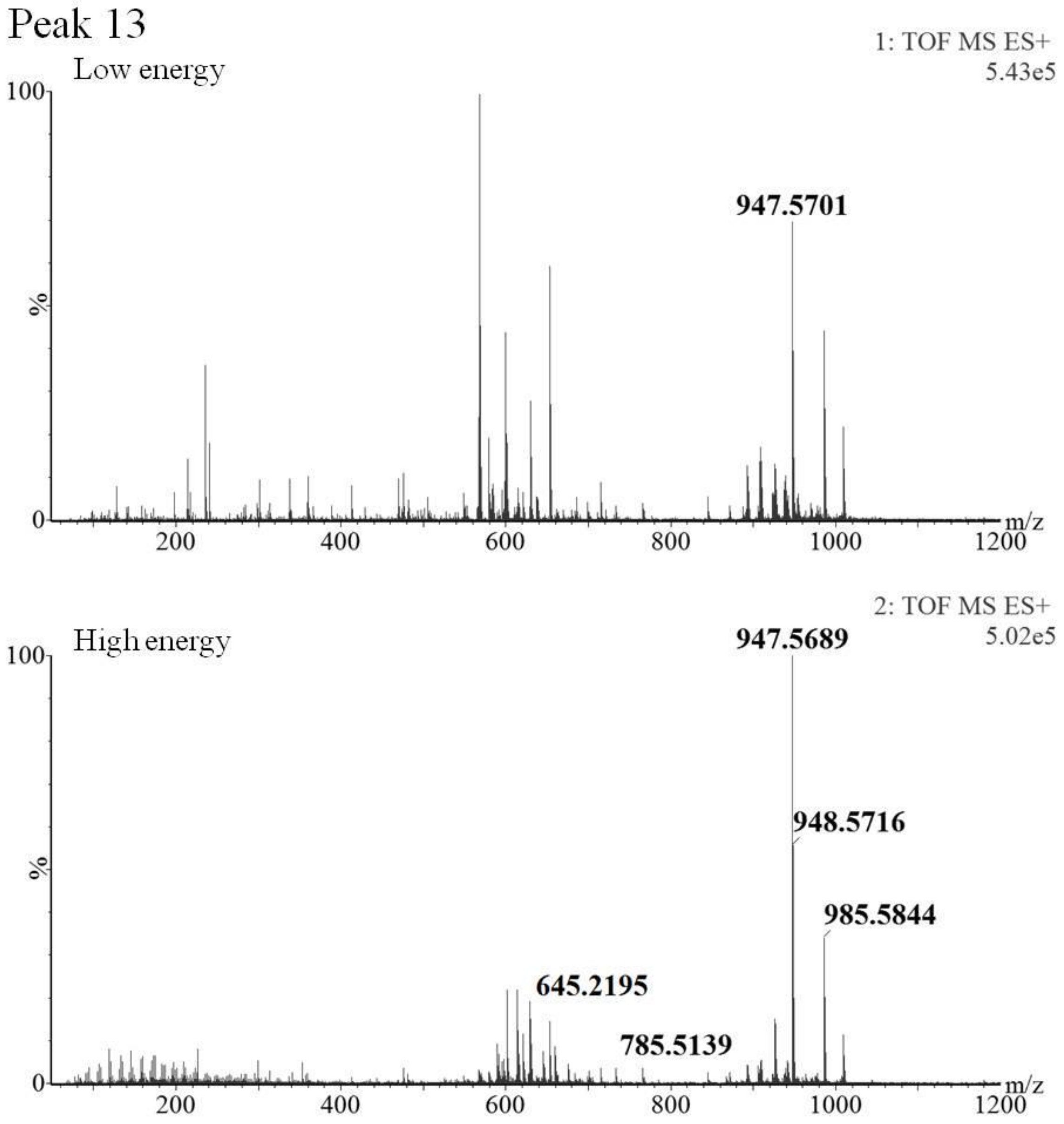


**Figure S2.** *Cont*.

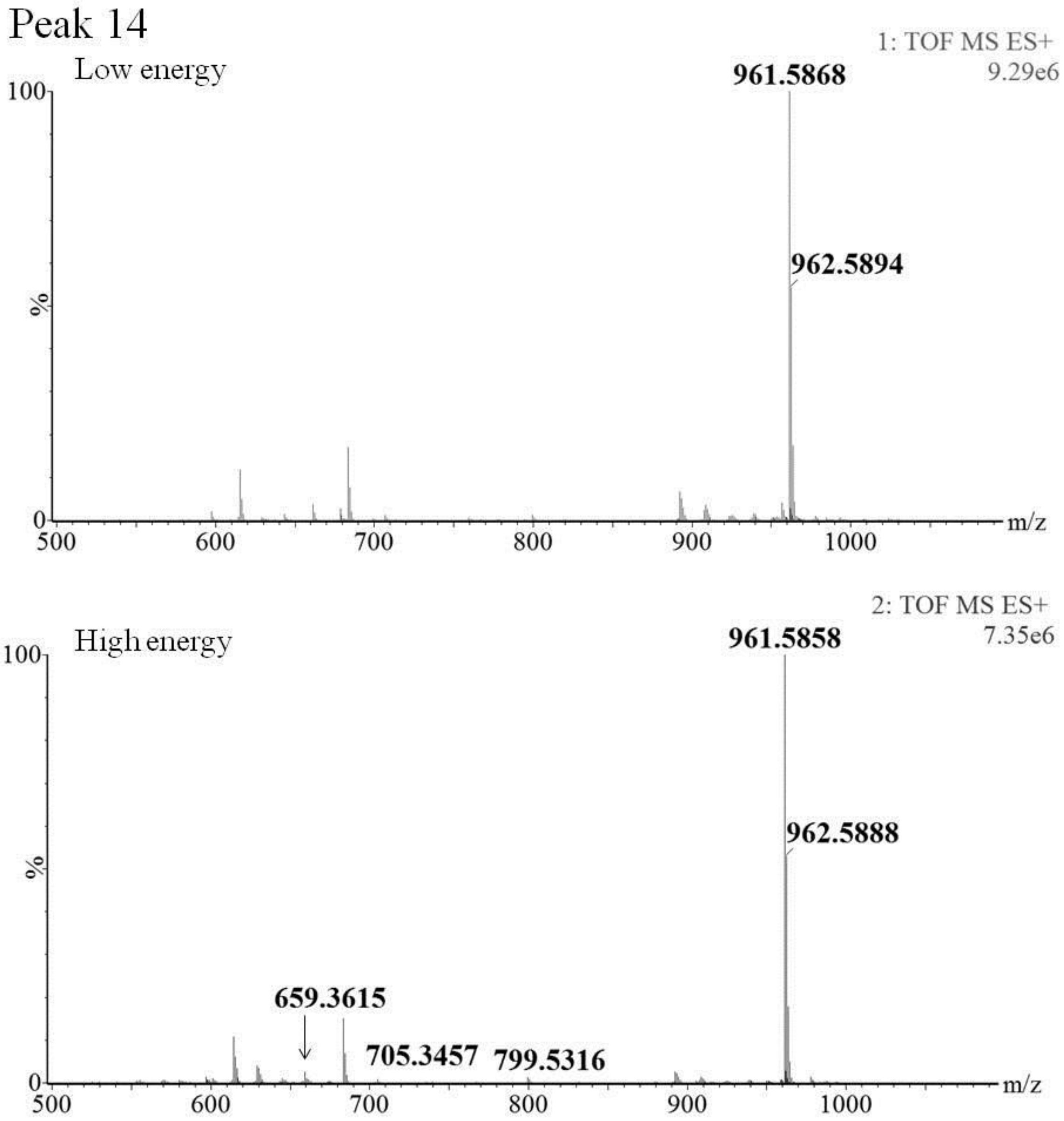


**Figure S2.** *Cont*.

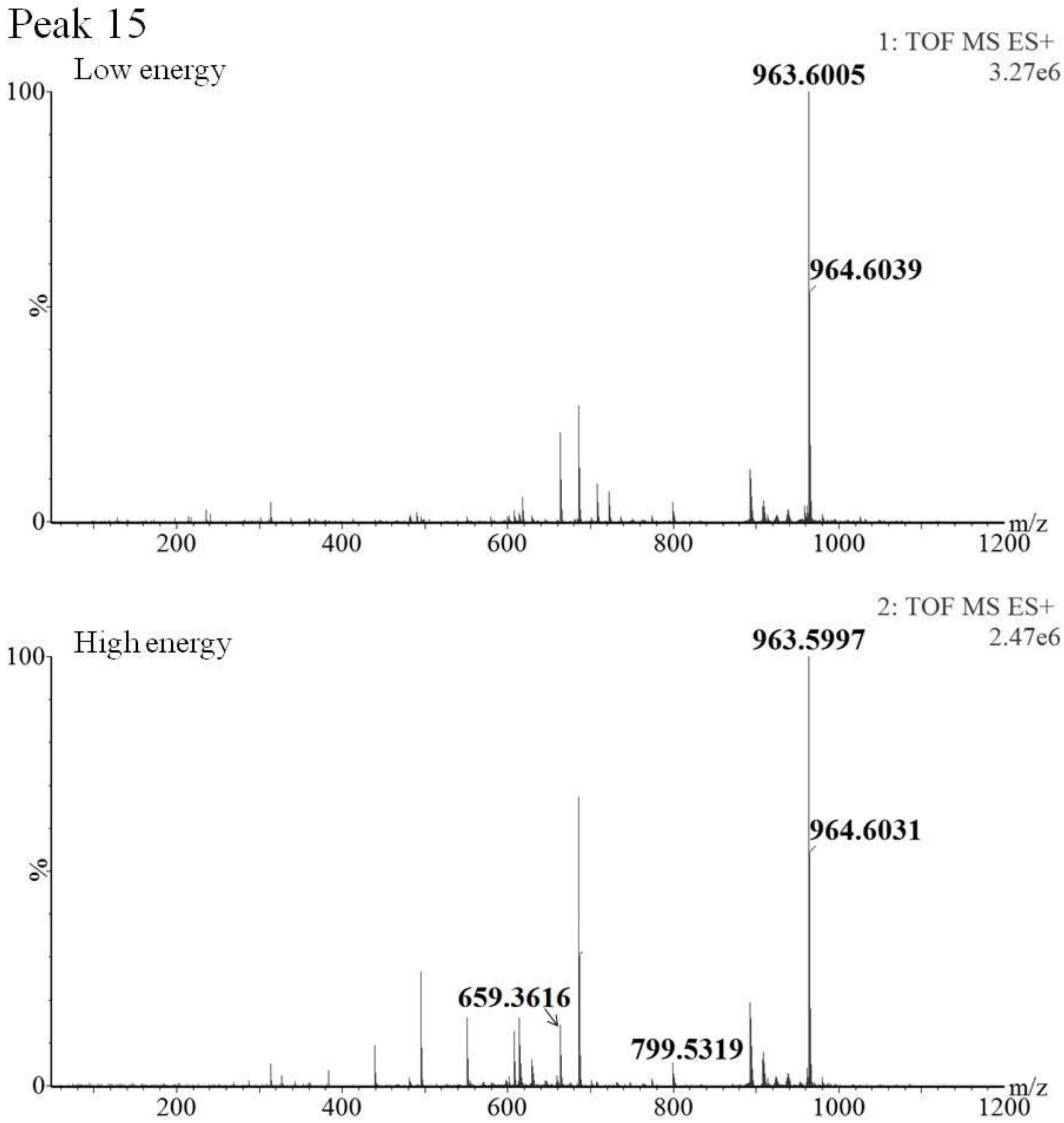


**Figure S2.** *Cont.*

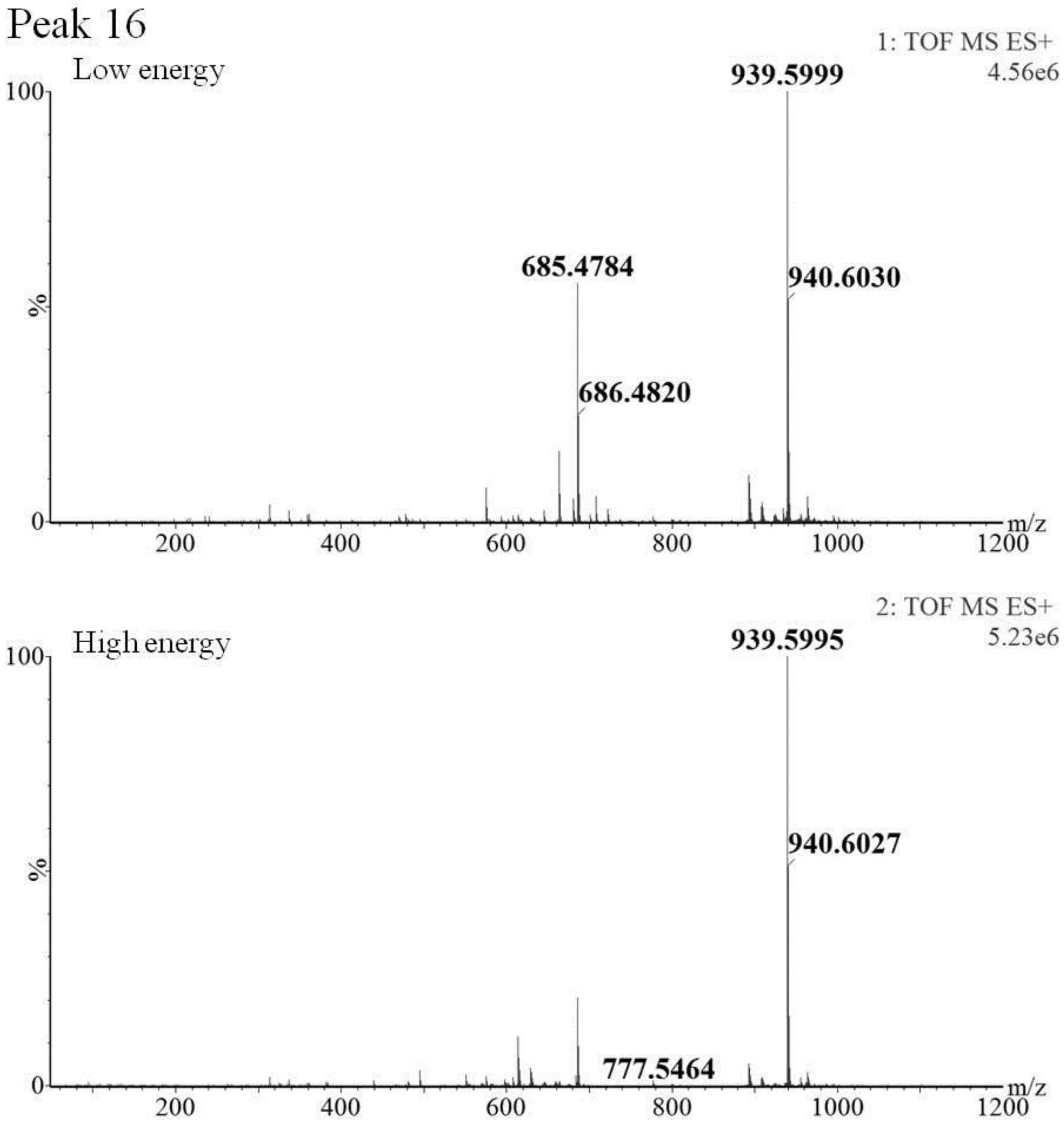


**Figure S2.** *Cont.*

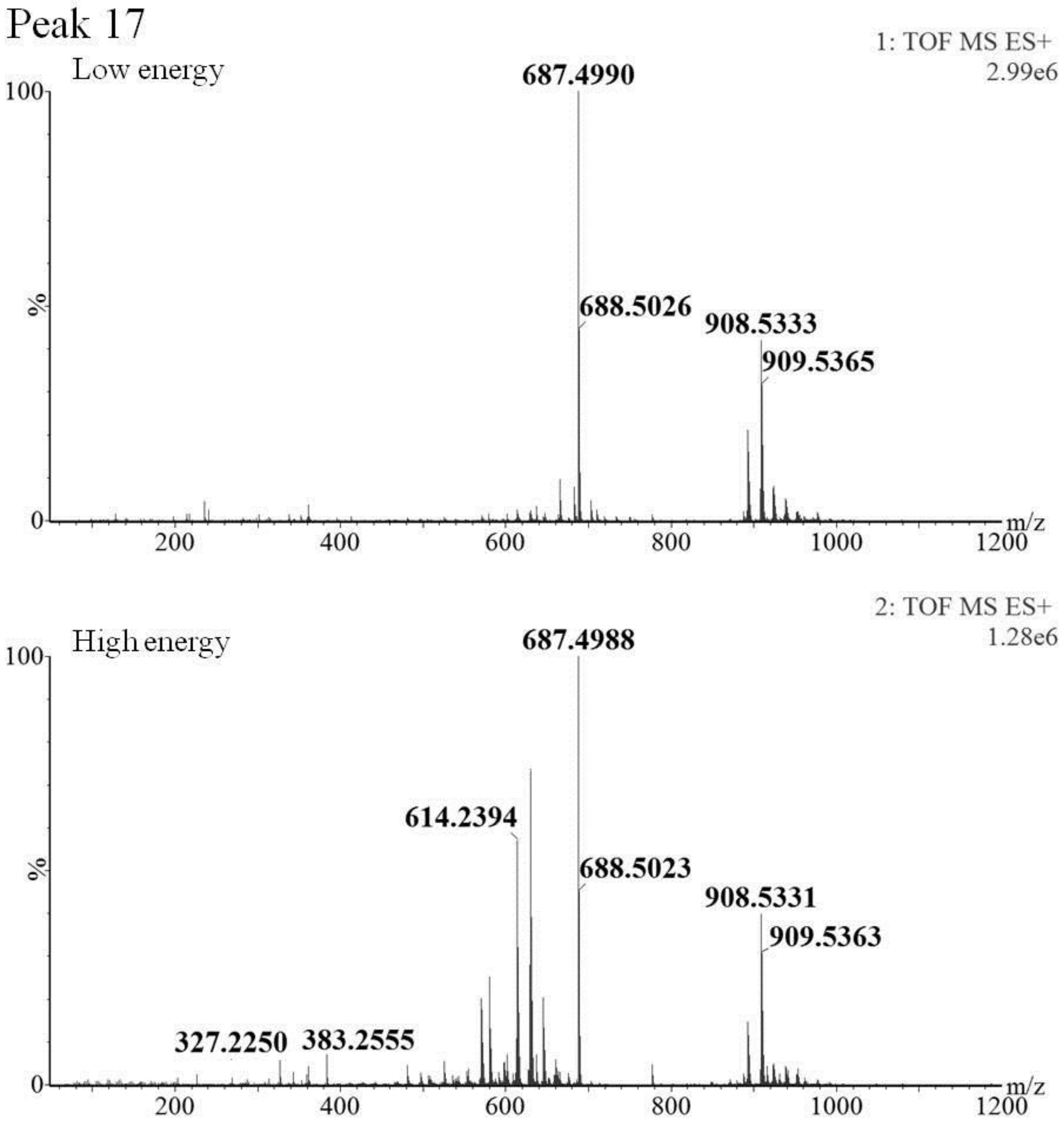


**Figure S2.** *Cont.*

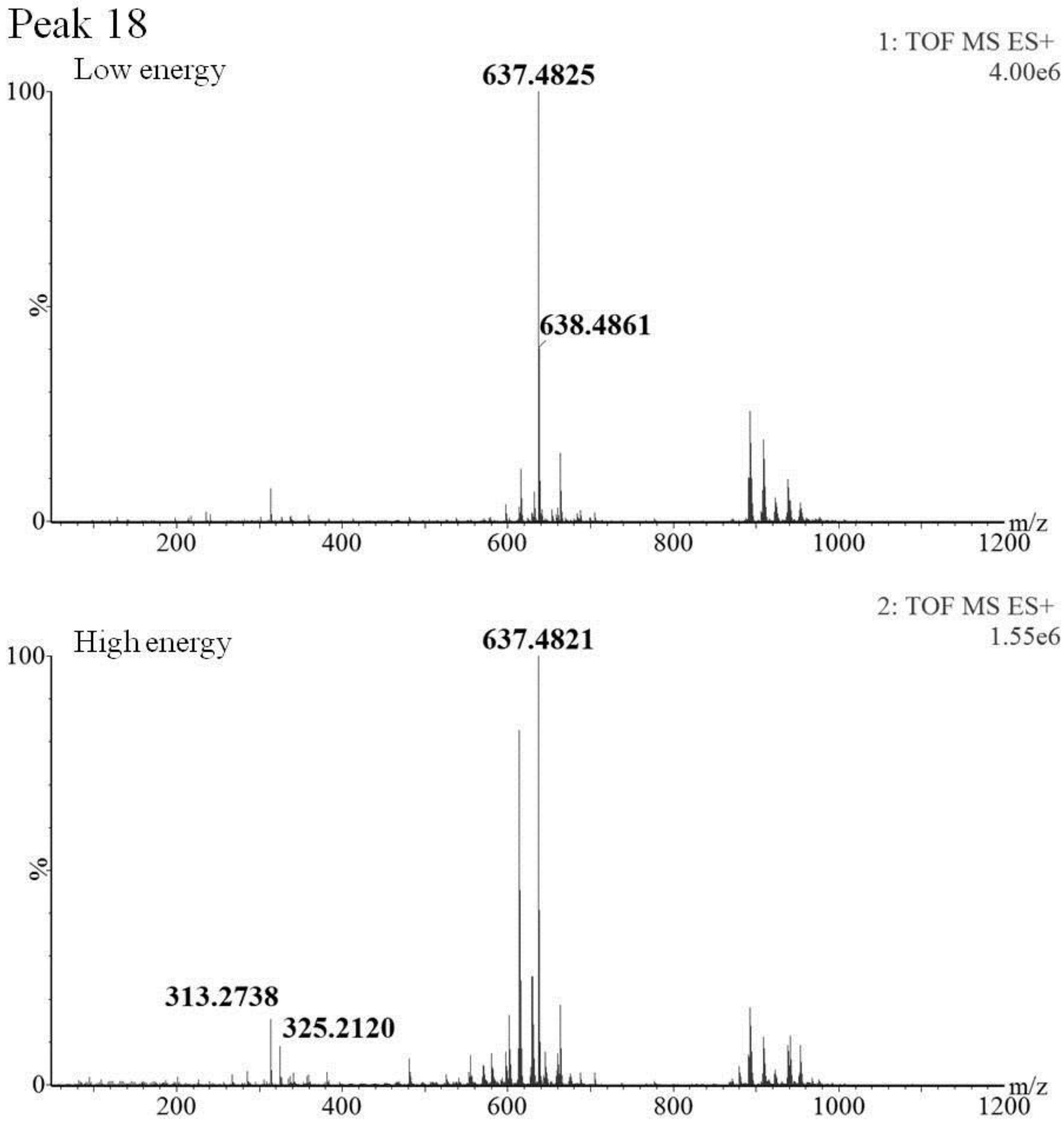


**Figure S2.** *Cont*.

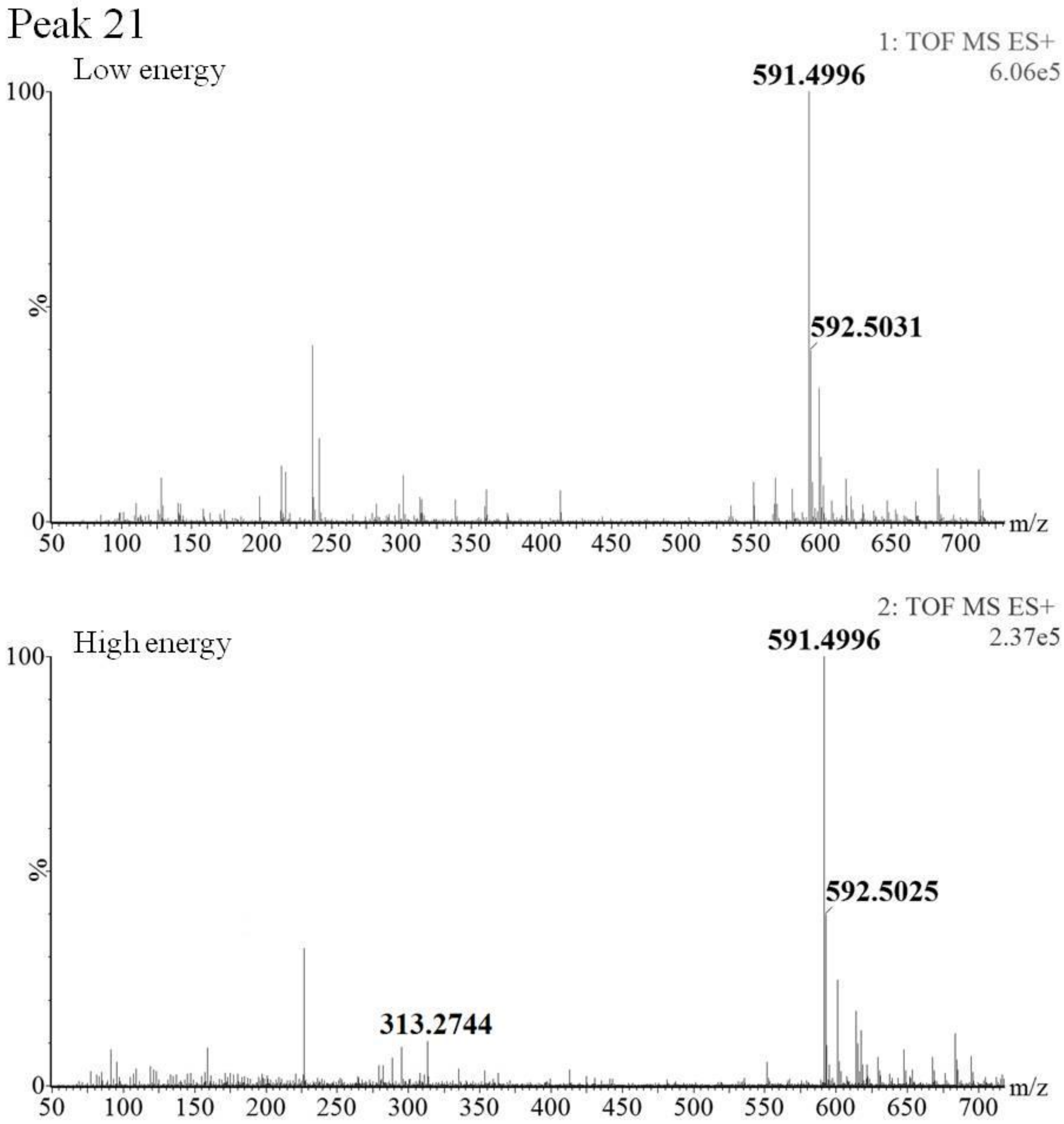


**Figure S2.** *Cont.*

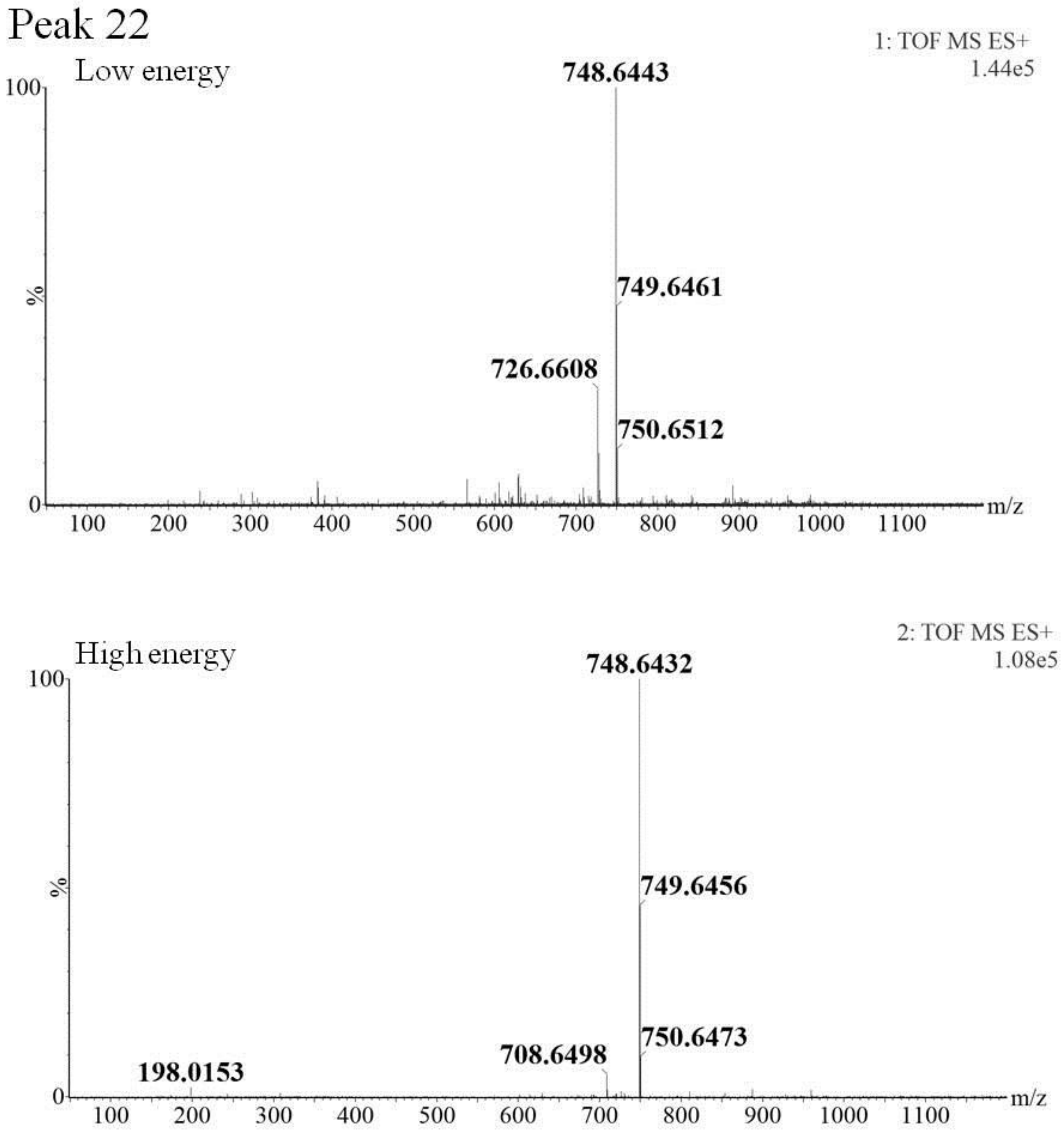


**Figure S2.** *Cont*.

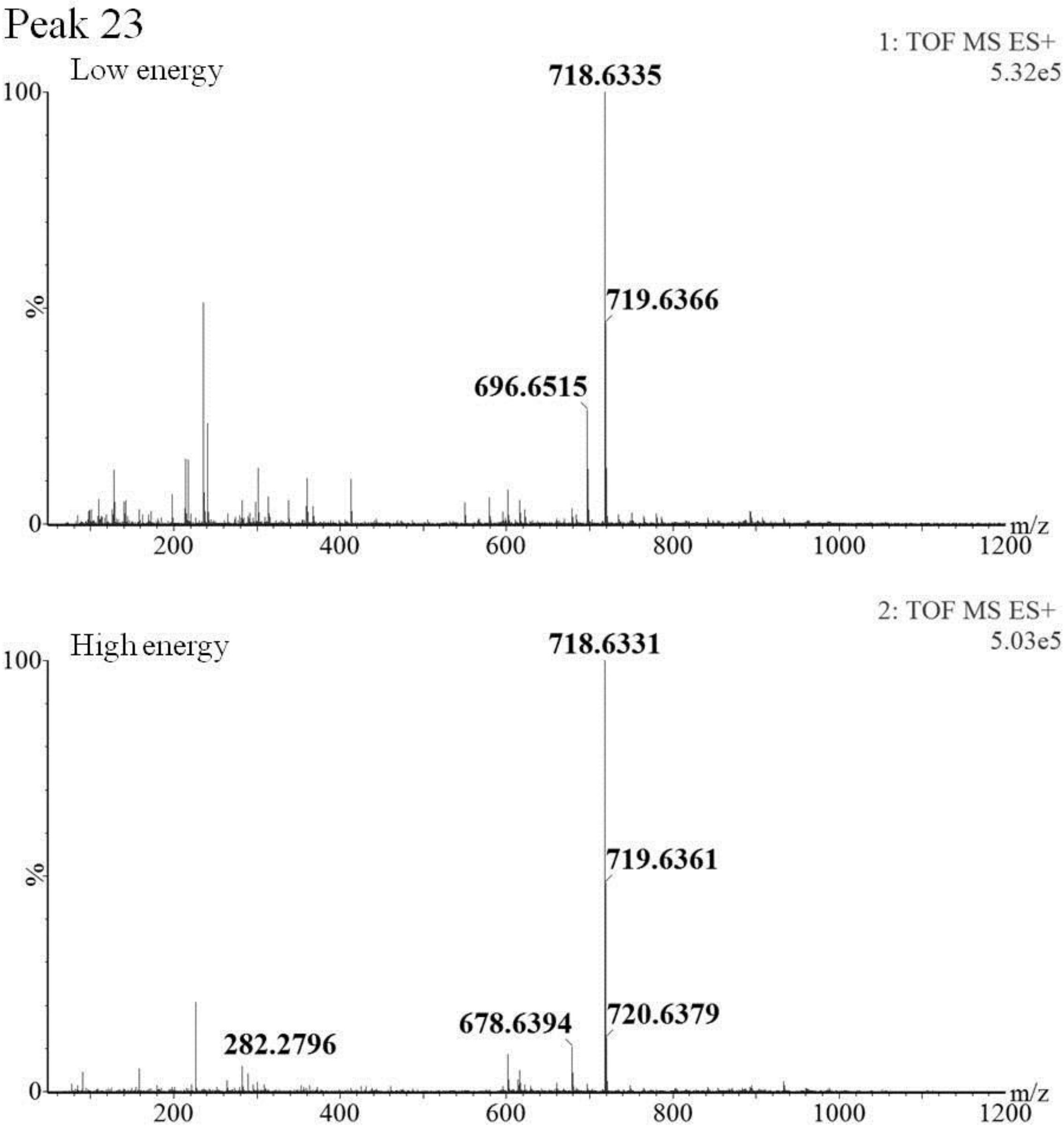


**Figure S2.** *Cont.*

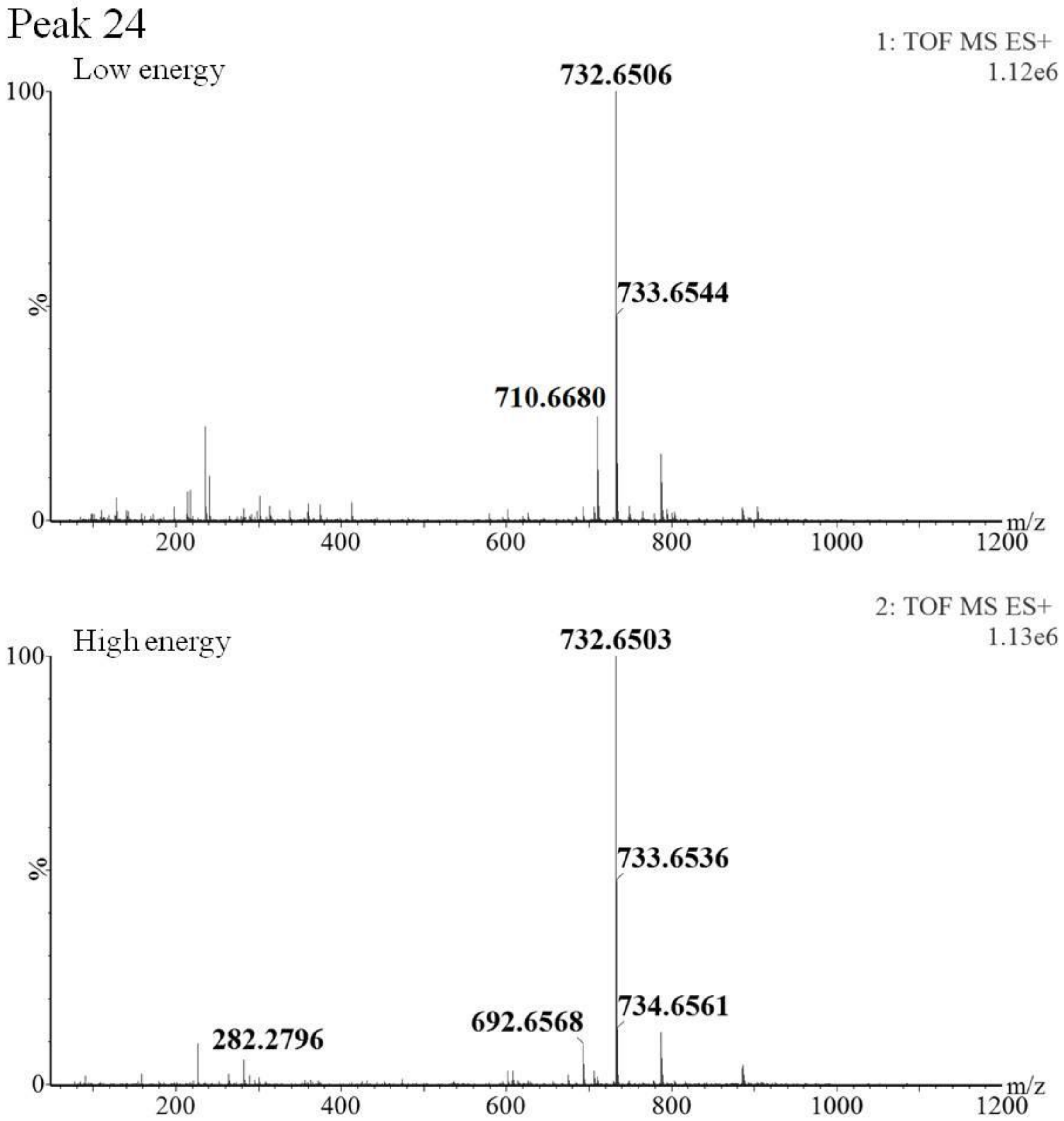


**Figure S2.** *Cont*.

Peak 28

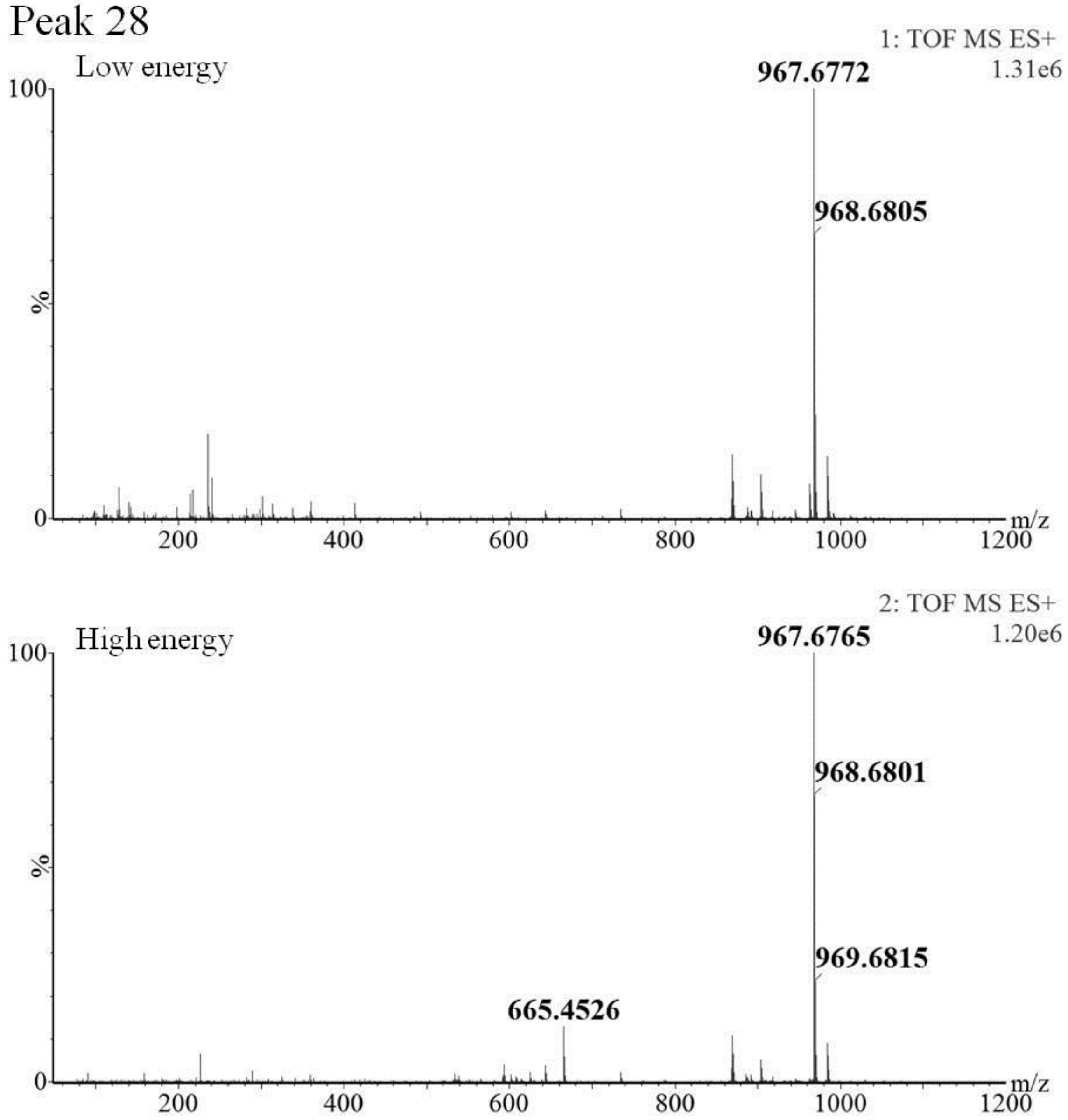


**Figure S2.** *Cont*.

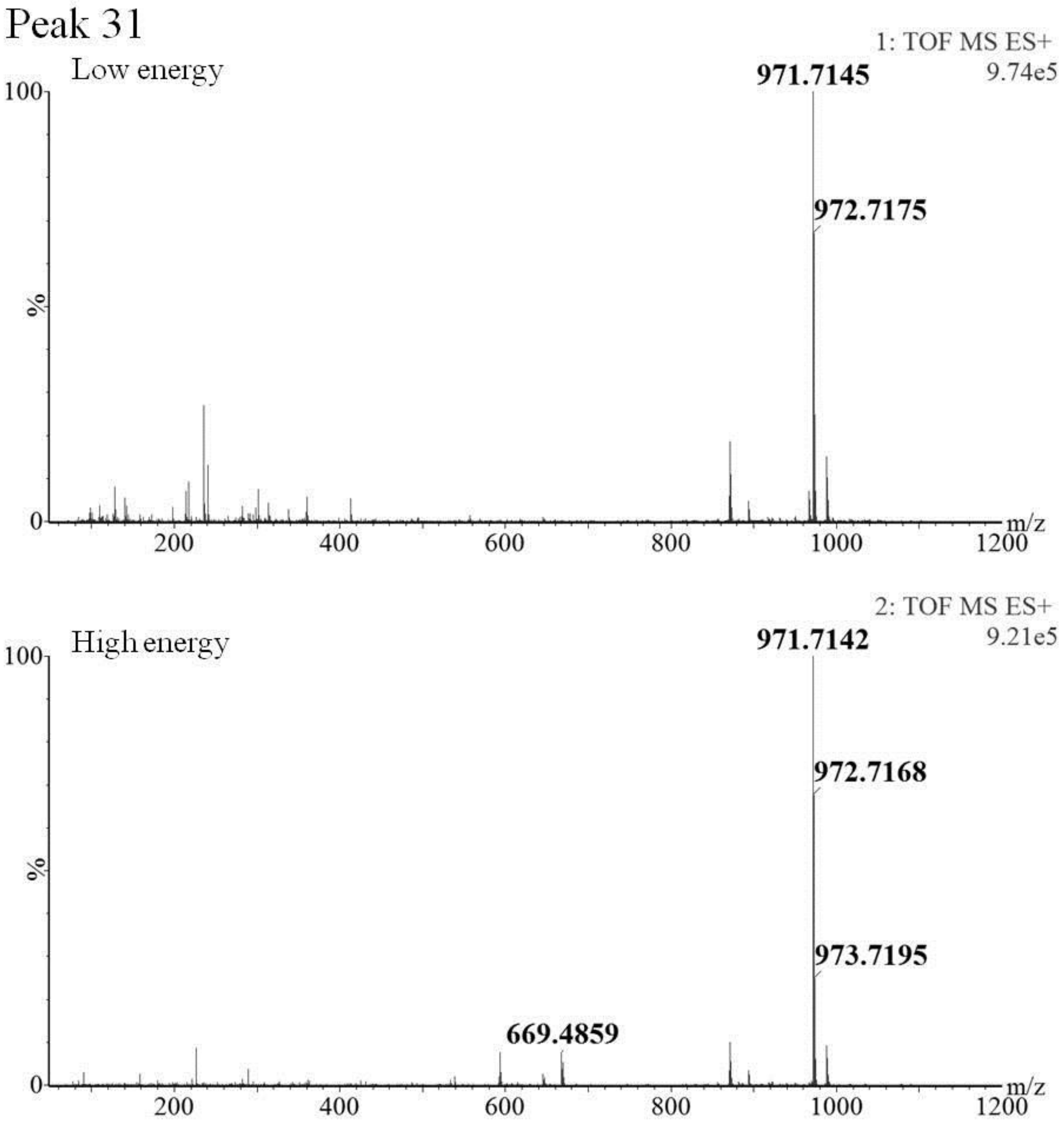


**Figure S2.** *Cont.*

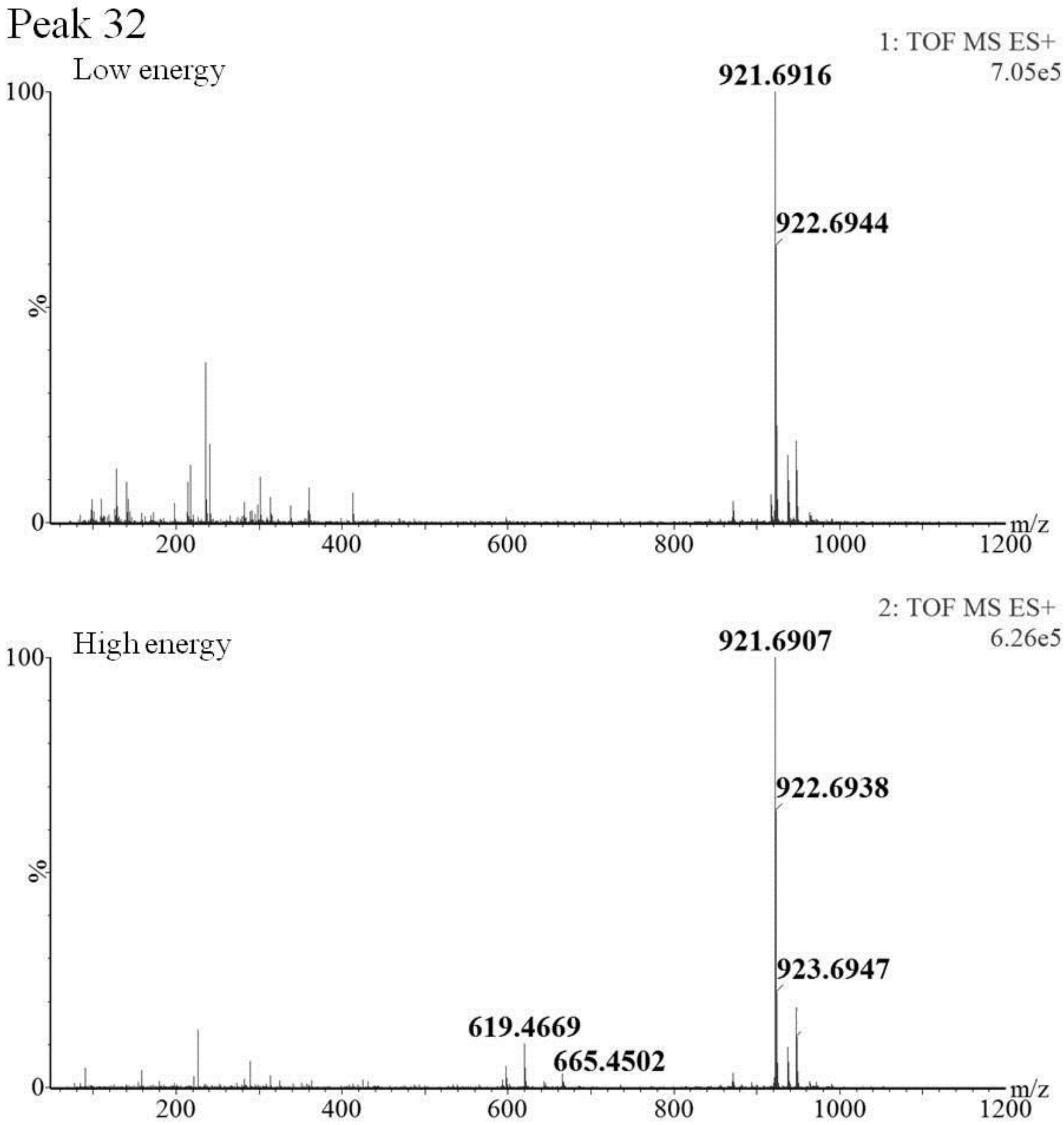


**Figure S2.** *Cont.*

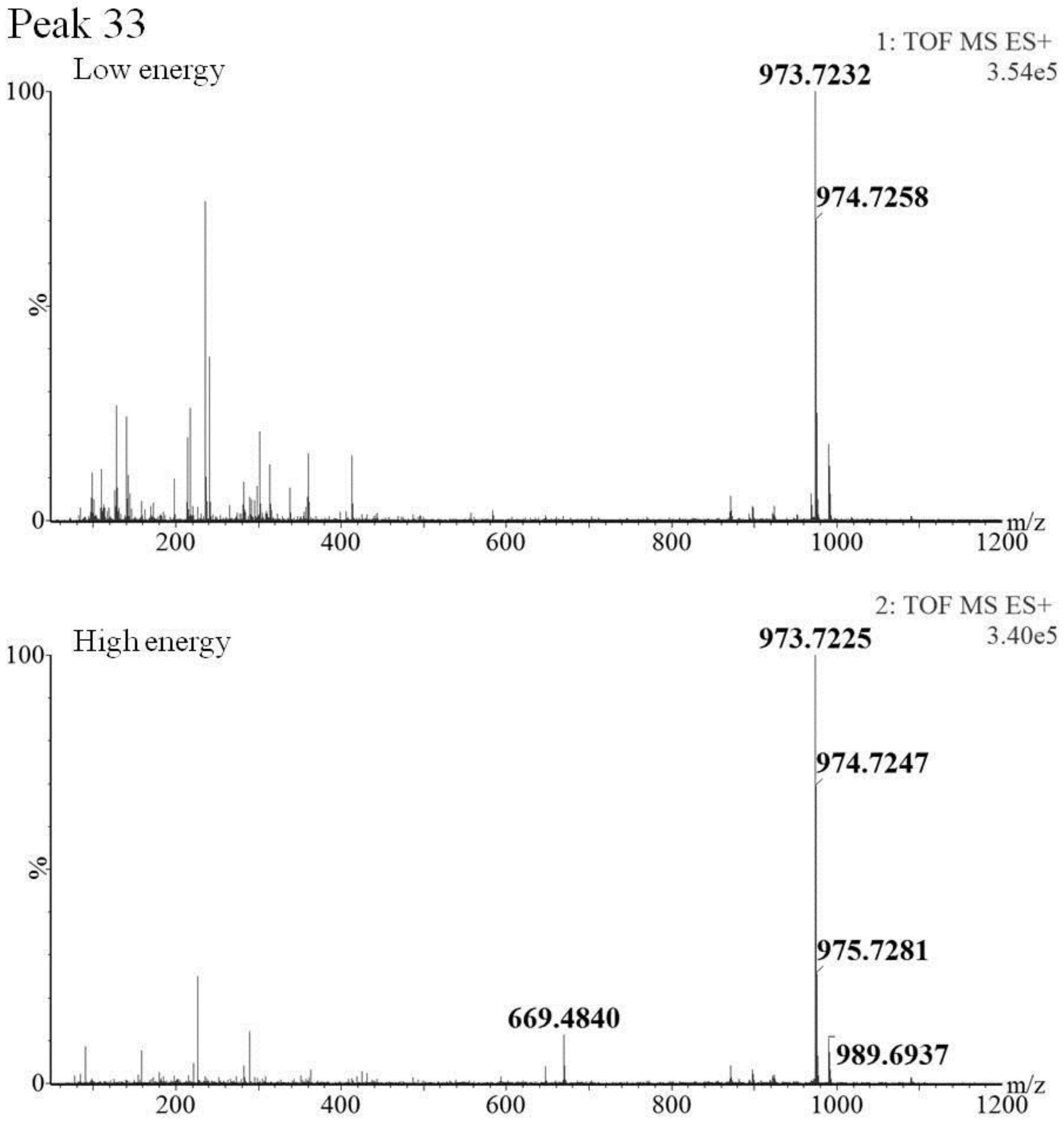


**Figure S2.** *Cont.*

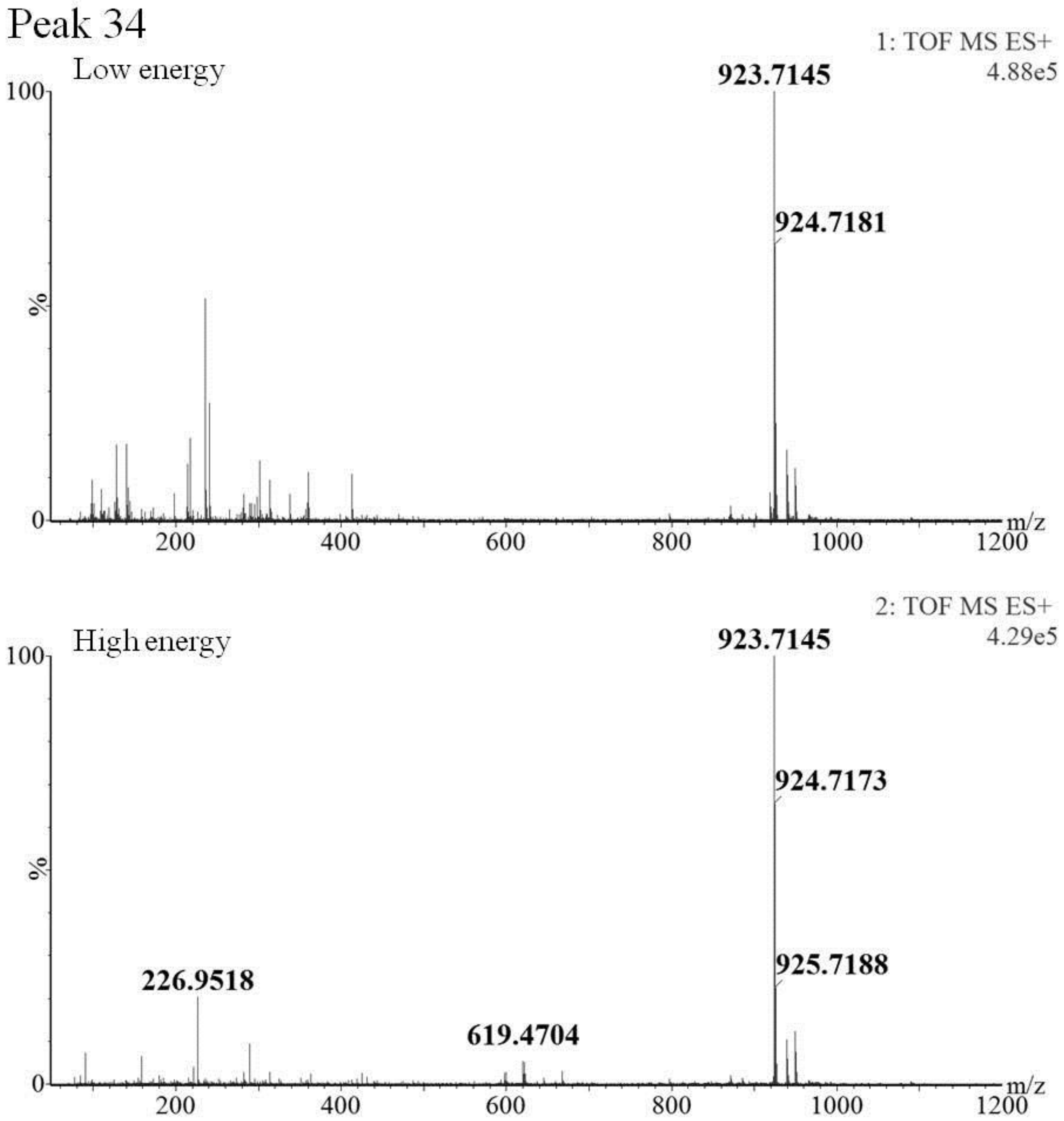


**Figure S2.** *Cont*.

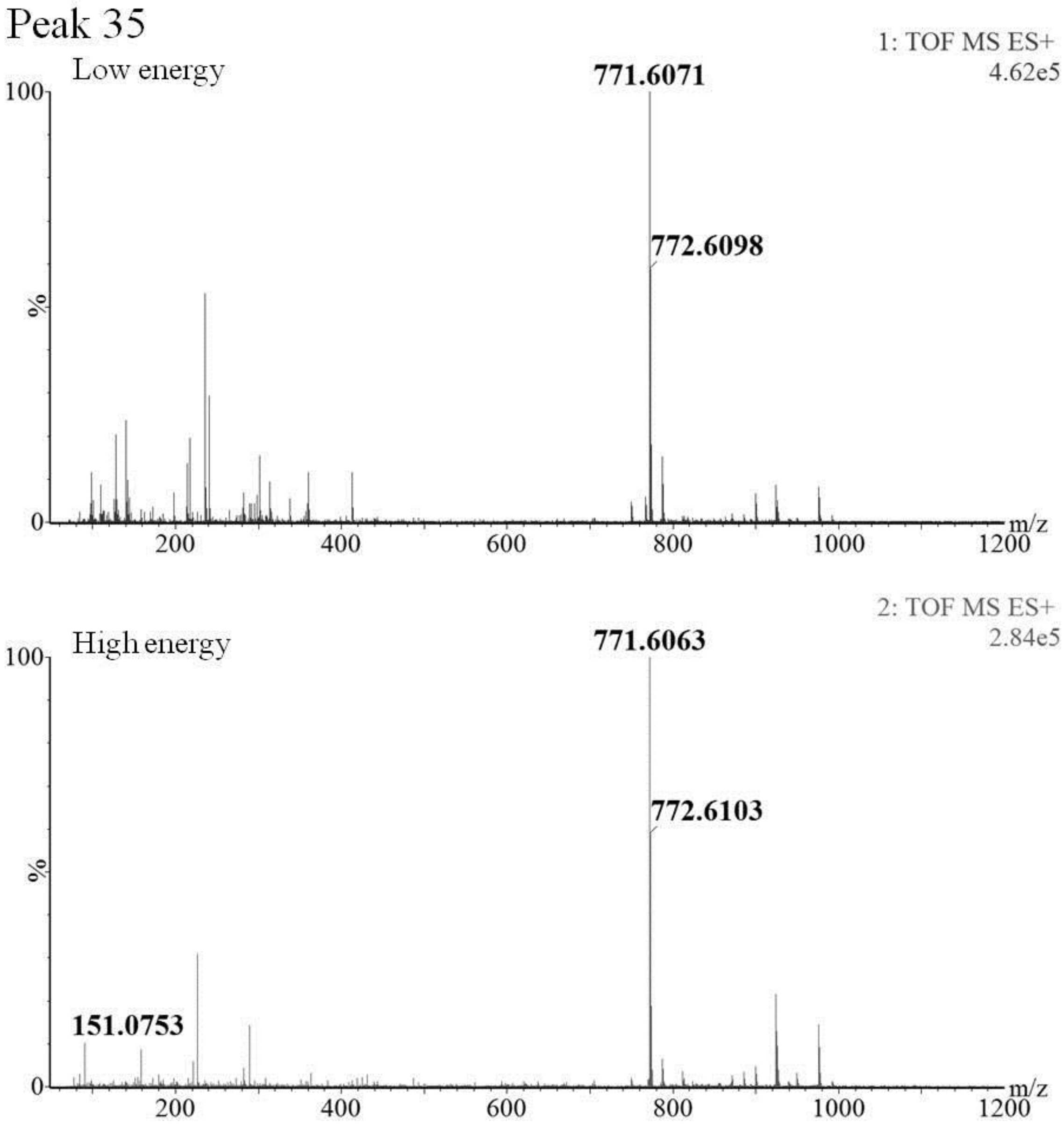


**Figure S2.** High resolution $MS^E$ spectra of unidentified ions in the *Pp* ethanol extract.

© 2015 by the authors; licensee MDPI, Basel, Switzerland. This article is an open access article distributed under the terms and conditions of the Creative Commons Attribution license (http://creativecommons.org/licenses/by/4.0/).
